# Targeting tRNA-synthetase interactions towards novel therapeutic discovery against eukaryotic pathogens

**DOI:** 10.1371/journal.pntd.0007983

**Published:** 2020-02-27

**Authors:** Paul Kelly, Fatemeh Hadi-Nezhad, Dennis Y. Liu, Travis J. Lawrence, Roger G. Linington, Michael Ibba, David H. Ardell

**Affiliations:** 1 The Ohio State University Molecular, Cellular and Developmental Biology Program, The Ohio State University, Columbus, Ohio, United States of America; 2 Center for RNA Biology, The Ohio State University, Ohio, United States of America; 3 Quantitative and Systems Biology Program, University of California, Merced, California, United States of America; 4 Department of Chemistry, Simon Fraser University, Burnaby, British Columbia, Canada; 5 Biosciences Division, Oak Ridge National Lab, Oak Ridge, Tennessee, United States of America; 6 Department of Microbiology, The Ohio State University, Columbus, Ohio, United States of America; 7 Department of Molecular & Cell Biology, University of California, Merced, California, United States of America; University of Florida, UNITED STATES

## Abstract

The development of chemotherapies against eukaryotic pathogens is especially challenging because of both the evolutionary conservation of drug targets between host and parasite, and the evolution of strain-dependent drug resistance. There is a strong need for new nontoxic drugs with broad-spectrum activity against trypanosome parasites such as *Leishmania* and *Trypanosoma*. A relatively untested approach is to target macromolecular interactions in parasites rather than small molecular interactions, under the hypothesis that the features specifying macromolecular interactions diverge more rapidly through coevolution. We computed tRNA Class-Informative Features in humans and independently in eight distinct clades of trypanosomes, identifying parasite-specific informative features, including base pairs and base mis-pairs, that are broadly conserved over approximately 250 million years of trypanosome evolution. Validating these observations, we demonstrated biochemically that tRNA:aminoacyl-tRNA synthetase (aaRS) interactions are a promising target for anti-trypanosomal drug discovery. From a marine natural products extract library, we identified several fractions with inhibitory activity toward *Leishmania major* alanyl-tRNA synthetase (AlaRS) but no activity against the human homolog. These marine natural products extracts showed cross-reactivity towards *Trypanosoma cruzi* AlaRS indicating the broad-spectrum potential of our network predictions. We also identified *Leishmania major* threonyl-tRNA synthetase (ThrRS) inhibitors from the same library. We discuss why chemotherapies targeting multiple aaRSs should be less prone to the evolution of resistance than monotherapeutic or synergistic combination chemotherapies targeting only one aaRS.

## Author summary

Trypanosome parasites pose a significant health risk worldwide. Conventional drug development strategies have proven challenging given the high conservation between humans and pathogens, with off-target toxicity being a common problem. Protein synthesis inhibitors have historically been an attractive target for antimicrobial discovery against bacteria, and more recently for eukaryotic pathogens. Here we propose that exploiting pathogen-specific tRNA-synthetase interactions offers the potential for highly targeted drug discovery. To this end, we improved tRNA gene annotations in trypanosome genomes, identified functionally informative trypanosome-specific tRNA features, and showed that these features are highly conserved over approximately 250 million years of trypanosome evolution. Highlighting the species-specific and broad-spectrum potential of our approach, we identified natural product inhibitors against the parasite translational machinery that have no effect on the homologous human enzyme.

## Introduction

Developing therapies against eukaryotic pathogens has proven challenging due to high conservation between the infectious agent drug target and their host counterpart [1]. Of particular concern is the trypanosome parasite *Leishmania* that infects upwards of 2 million individuals every year and accounts for more than 50,000 deaths annually [2]. While current treatments of amphotericin B and miltefosine are commonly prescribed to patients with leishmanial infections, they have undesired off-target cytotoxicity, leading to poor patient compliance and low-dose administration [3], and ultimately contributing to the rise of strain-dependent drug resistance [4,5]. There is a strong need for new nontoxic drugs with broad-spectrum activity against different species of *Leishmania* and other trypanosomes [6,7].

Given their essential role in protein synthesis, aminoacyl-tRNA synthetases (aaRSs) have been an attractive target for antimicrobial therapeutics [8]. AaRSs are essential enzymes found in all domains of life that are responsible for the correct pairing of free amino acids in the cell to their cognate tRNA [9]. AaRSs perform their activity in two steps: first, a free amino acid is activated by the enzyme through the hydrolysis of ATP, forming an aminoacyl-adenylate. Second, the amino acid is transferred to its corresponding tRNA before being released into the aminoacyl-tRNA pool [9]. Given the complex pool of free amino acids and uncharged tRNAs in the cell, aaRSs have co-evolved discrete mechanisms to ensure mutually exclusive amino acid activation and cognate tRNA recognition [10]. The sequence/structural determinants (or anti-determinants) that lead to accurate aaRS-tRNA recognition are also known as the tRNA identity elements. The primary tRNA identity elements that aid in cognate aminoacylation have been extensively studied for several decades [11,12]. For example, across all three domains of life, all tRNA^Ala^ isoacceptors contain a conserved G:U base pair in the acceptor stem that is recognized by alanyl-tRNA synthetase (AlaRS), leading to accurate Ala-tRNA^Ala^ synthesis in the cell [13–15].

While some aaRS inhibitors have successfully made it to the clinic, including the IleRS-targeting mupirocin [16], ProRS inhibitor halofuginone [17], and the LeuRS inhibitor tavaborole [18,19], there are likely many potential aaRS drugs still to be identified. Target-based approaches relying on structural data and sequence identity have previously been used to try and predict novel trypanosome aaRS drug targets [20–22] with some recent success [23]. While structure-based approaches have their utility, exploiting tRNA-aaRS interactions has been under-explored for its therapeutic potential. In particular, while interactions with small molecules are expected to be quite conserved across species, the evolutionary diversification of tRNA identity element interactions through coevolution with aaRSs opens the possibility of greater species-specific inhibition.

While major identity elements have been experimentally characterized for many aaRS-tRNA pairs in various model systems, much less is known about how tRNA identity elements evolve and diverge over the Tree of Life. Recent theoretical advances explain how tRNA identity elements can evolve and diverge in a phylogenetically informative way, even while under strong selective constraints [24]. In earlier work, we developed a bioinformatic method to predict tRNA identity elements [25]. Our bioinformatic predictions are called Class-Informative Features (CIFs), based on the statistic of structure-conditioned Shannon Information [26], and visualized through graphs called Function Logos [25]. In later work, we applied two other statistics, Information Difference and Kullback-Leibler Divergence, to facilitate pairwise comparisons of CIFs between two taxa, in two new visualizations called Information Difference (ID) logos and Kullback-Leibler Divergence (KLD) logos, respectively [27]. ID logos visualize gains and losses of CIFs, which in this work we call gains and losses of information, while KLD logos visualize the functional conversion of CIFs from one functional type of tRNA to another, which in this work we call change of functional information. In the present work, we integrate together all three statistics (structure-conditioned information about function, ID, and KLD) and apply it to the problem of identifying parasite-specific tRNA identity elements. Our approach visualizes functionally informative features in parasite tRNAs that have either gained or retained functional information relative to humans, altered functional associations, or both, since divergence from their common ancestor with humans.

Our modeling approach integrates genomic tRNA sequence variation across multiple tRNA gene families of different functions, revealing potentially useful information about the specification of substrate identity for all aaRSs simultaneously. The multiplicity of aaRSs in cells provides multiple potential targets for inhibition of essential parasite enzymes, opening the door to improved combination chemotherapies. Advances in systems biology and chemogenomics have fueled interest in combination chemotherapies to benefit from synergistic drug interactions [28–31] and combat the evolution of resistance [32]. Combination chemotherapies are naturally effective, for example, in the pathogenic defenses of arthropods [33] and have yielded exciting antifungal [34] and antihelminthic [35] therapies. Additionally, artemisinin-based combination therapies (ACTs) are the primary treatment plan for *Plasmodium falciparum* malaria infections [36,37].

Here, we report our improved annotation of TriTrypDB genomes and new methodologies for predicting conserved identity elements across biological domains. As proof of principle, we screened for identity element divergence between trypanosomes and humans to search for new therapeutic targets for these eukaryotic pathogens. Validating our computational approaches, we found several natural product fractions that inhibit *Leishmania major* AlaRS activity but have no effect on the homologous human enzyme. The fractions we identified also have inhibitory activity against *Trypanosoma cruzi* AlaRS, showing that our approach holds promise towards identifying new broad-spectrum anti-trypanosomal therapies.

## Methods

### Annotation, clustering and filtering of tRNA genes in TriTrypDB genomes

We downloaded data for 46 genome assemblies from TriTrypDB version 41 released December 5^th^, 2018. We ran tRNAscan-SE v.2.0.0 installed via BioConda in February, 2019 [38] and Aragorn v.1.2.38 [39] using option “-i116” (implying a maximum intron length in search targets of 116 base-pairs) on this data. We unified gene records from the two finders if they overlapped by at least one base-pair, had consistent strand-orientations and end-displacements less than or equal to 4 bp. To independently identify initiator tRNA genes, we computed edit distances [40] of CAT-anticodon-containing genes implemented in the function stringdist() from its R package v. 0.9.5.5 and clustered them agglomeratively using Ward’s minimum variance method [41] implemented in the function hclust() with method ward.D2 from the base R stats package, examining clusters for the initiator-distinguishing features described in [42]. All statistical analyses and sequence processing for annotation and clustering were carried out in R v.3.6 [43].

To further investigate these gene records, we examined their genetic clustering in TriTrypDB genomes as defined by co-occurrence within a distance of 1000 bp on either strand. We computed gene function content distances of tRNA gene-clusters as pairwise Jaccard distances considering gene clusters as sets of functions using stringdist() and clustered them with Ward’s method using function hclust() with method ward.D2. We finalized our annotation union gene-set by retaining 3616 genes that had an Aragorn score above 106 bits or a tRNAscan-SE2 score above 49 bits, and reannotating sequences as described in the Results.

### Prediction of divergent tRNA Class-Informative Features (CIFs) in humans and parasites

To compare CIFs between TriTrypDB genomes and humans and to have sufficient data to estimate trypanosome CIFs, we defined eight phylogenetic clades for 39 of the 46 trypanosome genomes as shown in Table 1. These clades were based on a composite of phylogenetic results in the literature [44–47]. CIFs were subsequently estimated for each clade independently, by pooling tRNA genes within clades. We removed two incomplete genomes from analysis, *T*. *rangeli* SC58 and *T*. *cruzi* CL-Brenner that had fewer than half the number of tRNA genes identified in any other genome and missed more than two functional classes. We filtered the gene annotation union gene set of 3616 genes, removing selenocysteine genes, pseudogenes, truncated genes, and genes of ambiguous function, leaving 3488 high-confidence functionally annotated gene records from 44 genomes in TriTrypDB v.41 for alignment. To this set we added 431 high-confidence human tRNA gene records downloaded from GtRNADB [48] on May 15, 2019 (in the file “hg38-tRNAs.fas”), excluding two human selenocysteine tRNA genes, to yield a grand total of 3919 tRNA genes from 45 genomes for structural alignment. We aligned this alignment gene-set of 3919 genes using COVEA v.2.4.4 [49] to the eukaryotic tRNA covariance model supplied with tRNAscan-SE v.1 [50]. The output alignment was manually edited in SEAVIEW [51] to correct the misalignment of 595 human and trypanosome tRNA genes (almost exclusively of type tRNA^Leu^ and tRNA^Ser^) at Sprinzl coordinates 45 and 47 and exclude majority-gap/insertion and variable-arm-containing sites (Sprinzl coordinates are a standardized coordinate system that encodes both the consensus universal secondary structure of tRNAs, and conserved, more functionally-specific structures like the long variable arms of tRNA^Leu^ and tRNA^Ser^ [52]). Sequences were further processed with the FAST toolkit to partition genes into clades [53]. After excluding an additional 464 genes from five genomes not included in our defined clades, 3455 aligned trypanosome and human genes remained. More statistics on the CIF estimation gene sets by clade are shown in Table 1 and additional notes and code to reproduce the data workflow are provided in Supplementary Online Materials (Code and Data).

**Table 1:** Clades and genomes analyzed, with statistics on CIF estimation gene sets.

Clade	Genome Assemblies	Total Genes	Mean Genes/ Genome (Std. Dev.)	Grand Mean %G	Grand Mean %C	Grand Mean %T
**Humans**	Hg38	431	431	32.5	25.7	23.0
***L*. *major*** **Clade (n = 8)**	1. *L*. *major* Friedlin 2. *L major* LV39c5 3. *L*. *major* SD75 4. *L*. *tropica* L590 5. *L*. *aethiopica* L147 6. *L*. *gerbilli* LEM452 7. *L*. *turanica* LEM423 8. *L*. *arabica* LEM1108	664	83 (0.6)	31.9	26.1	23.4
***L*. *infantum*** **Clade (n = 3)**	1. *L*, *donovani* BHU1220, 2. *L*. *donovani* BPK282A1 3. *L*. *infantum* JPCM5	250	83.33 (0.2)	31.9	26.1	23.3
***L*. *mexicana*** **Clade (n = 2)**	1. *L*. *amazonensis* MHOMBR71973M2269 2. *L*. *mexicana* MHOMGT2001U1103	148	74 (4.7)	31.8	25.9	23.4
**Viannia** **Subclade (n = 4)**	1. *L*. *braziliensis* MHOMBR75M2904 2. *L*. *braziliensis* MHOMBR75M2903 3. *L*. *panamensis* MHOMPA94PSC1 4. *L*. *panamensis* MHOMCOL81L13	327	81.75 (2.5)	31.8	26.1	23.4
***L*. *enriettii*** **Clade (n = 2)**	1. *Leishmania* sp MARLEM2494 2. *Leishmania enriettii* LEM3045	160	80 (0.5)	31.9	25.9	23.4
***Leptomonas/*** ***Crithidia* Clade (n = 3)**	1. *Crithidia fasciculata* CfCl 2. *Leptomonas seymouri* ATCC30220 3. *Leptomonas pyrrhocoris* H10	300	100 (2.4)	31.8	26.0	23.3
**American *Trypanosoma*** **(n = 11)**	1. *T*. *grayi* ANR4 2. *T*. *cruzi* CL Brener Esmeraldo-like 3. *T*. *cruzi* CL Brener Non-Esmeraldo-like 4. *T*. *cruzi cruzi* Dm28c 5. *T*. *cruzi* Dm28c 6. *T*. *cruzi* Esmeraldo 7. *T*. *cruzi* JRcl4 8. *T*. *cruzi* marinkelleiB7 9. *T*. *cruzi* SylvioX10-1 10. *T*. *cruzi* SylvioX10-1-2012 11. *T*. *cruzi* Tulacl2	773	70.27 (5.7)	32.0	26.3	23.0
**African *Trypanosoma*** **(n = 6)**	1.*T*. *bruceigambiense* DAL972 2.*T*. *brucei* Lister427 3.*T*. *brucei* TREU927 4.*T*. *evansi* STIB805 5.*T*. *congolense* IL3000, 6. *T*.*vivax* Y486	402	67 (2.1)	32.2	26.1	23.1

For each clade independently, we computed function logos [25], Information Difference logos and Kullback-Leibler Divergence logos [27] with a newly written Python 3 program tSFM (tRNA Structure-Function Mapper) v0.9.14 available on github (https://github.com/tlawrence3/tsfm), which we describe briefly here, and more fully in a forthcoming publication. tSFM provides a command-line user interface for estimating function, ID, and KLD logos using our published methods. tSFM additionally calculates tRNA CIFs for secondary-structure feature pairs, in addition to single-site features. Class-Informative Feature Pairs are elements of the Cartesian product set *C = f × f × BP*, where f = {*A,C,G,U,*−} is the set of single-site features we consider and *BP* is the set of structurally-paired Sprinzl Coordinates involved in potential base-pairing interactions along the four arms of the planar clover-leaf consensus secondary structure of tRNAs [52]. We ran tSFM with option “-x 1” corresponding to computing exact expected entropies for samples of size one by the method of [54] or by the Bayesian Nemenman–Shafee–Bialek (NSB) entropy estimator [55] otherwise.

Briefly, we computed the gain-of-information of a CIF in a particular functional class and trypanosome clade as its information difference in bits, with that clade as foreground and humans as background, multiplied by the normalized ratio of posterior-to-prior odds of the CIF in that functional class in trypanosomes and humans, corresponding to letter heights in ID logos, and measured in bits. We computed change-of-function of a CIF in a particular functional class and trypanosome clade as its Kullback-Liebler Divergence in bits, with that clade as foreground and humans as background, multiplied by the normalized ratio of posterior-to-prior odds of the CIF in that functional class, corresponding to letter heights in KLD logos and measured in bits. To avoid division by zero when calculating KLD, we added pseudocounts to either the background or the foreground posterior distributions when one or more of the 21 functional classes was not observed. When calculating the normalized ratio of posterior-to-prior odds for a specific functional class, we only added pseudocounts to the background posterior distribution. Furthermore, to avoid inaccuracies, we defined the KLD of a feature to be zero when its frequency in the background is less than or equal to five.

We wrote a custom script in R 3.6 to visualize CIFs within each cluster for each functional class of tRNA in a structural context, and color the parasite CIFs according to whether those CIFs have gained information or changed functional information relative to human since divergence from their common ancestor. All data and scripts are provided as supplementary data.

### AaRS cloning and protein purification

*Leishmania major* (*Lm*) AlaRS and *Lm* ThrRS-encoding genes were codon optimized, synthesized, and sub-cloned into pUC57 (GenScript). Engineered flanking NdeI and SmaI restriction sites were used to clone the aaRS genes into pTYB2, creating in-frame C-terminal intein fusions. The resulting expression vectors were transformed into the *E*. *coli* expression strain BL21 (DE3). The gene encoding *Trypanasoma cruzi* (*Tc*) AlaRS was codon optimized, synthesized, and directly cloned into NdeI and XhoI cut sites in the pET21b expression vector (GenScript). The resulting plasmid expressed *Tc* AlaRS under T7 control and was in-frame with a C-terminal 6x-His tag. The pET21b-*Tc* AlaRS vector was transformed into the *E*. *coli* expression strain XJb (DE3) (Zymo Research).

Both *Lm* AlaRS and ThrRS were purified by growing cells to an OD600 of ~0.5 and cooling on ice for 30 minutes. Protein induction was initiated by the addition of IPTG to a final concentration of 500 μM and cells continued to grow at 16°C for 16 hours. Cells were harvested by centrifugation and lysed by sonication in Buffer A (25 mM HEPES pH 7.2, 500 mM NaCl, 3 mM DTT) with cOmplete mini protease inhibitor (Sigma) added. Clarified lysate was added to a chitin resin column (NEB) and washed with Buffer A. The intein tag was cleaved by the incubation of Buffer B (25 mM HEPES pH 7.2, 100 mM NaCl, and 100 mM DTT) on the resin bed overnight at 4°C. Protein was dialyzed in two stages in Buffer C (25 mM HEPES pH 7.2, 30 mM NaCl, 6 mM BME, and 10% - 50% glycerol).

*Trypanasoma cruzi* (*Tc*) AlaRS-expressing cells were grown to an OD600 ~0.3 and then cooled to 18°C and induced with 500 μM IPTG. Cells were grown for an additional 16 hours at 18°C before harvesting by centrifugation. Cell pellets were re-suspended in lysis buffer [Buffer I (500 mM Tris-HCl pH 8.0, 300 mM NaCl, and 10 mM imidazole) with cOmplete mini protease inhibitor (Sigma)], sonicated, clarified, and cell lysate passed over a TALON metal affinity column (Takara). After washing the column with Buffer I, protein was eluted with Buffer II (Buffer I with 250 mM imidazole). Protein was dialyzed in two stages to remove imidazole and to store the enzyme in 50% glycerol.

Human AlaRS was expressed in *E*. *coli* Rosetta (DE3) (Novagen) from pET21a which encodes the human AlaRS gene in-frame with a C-terminal 6x-His tag (expression plasmid provided by Karin Musier-Forsyth, Ohio State University). Cells were grown to an OD600 of ~0.5 and cooled on ice for 30’ before inducing expression with 500 μM IPTG. Upon induction, cells grew for an additional 16 hours at 20°C before harvesting. Human AlaRS was purified as described above with the addition of 5 mM β-mercaptoethanol to both Buffer I and Buffer II. All enzyme concentrations were determined by active site titration [56,57] using [^14^C]-alanine (Perkin Elmer) and [^14^C]-threonine (American Radiochemicals).

### Preparation of *in vitro* transcribed tRNA

*Lm* tRNA^Ala^ (chr11. trna1-AlaCGC), *Lm* tRNA^Thr^ (chr23. trna6-ThrTGT), and *Tc* tRNA^Ala^ (TctRNA-Ala.03) DNA sequences were cloned into EcoRI and XbaI restriction sites in pUC18 by slow cooling complementary synthetic DNA oligos and ligation as previously described [58]. PCR was used to amplify 50 μg DNA template from the pUC18-tRNA plasmids to be used for T7 runoff transcription. *In vitro* transcription was performed with 40 mM Tris-HCl pH 8, 2 mM spermidine, 22 mM MgCl_2_, 5 mM DTT, 50 μg/mL BSA, 4 mM NTPs, 20 mM 5’GMP, 20 U Protector RNase Inhibitor, 2 U pyrophosphatase, DNA template, and T7 RNAP at 42°C for 16 hours. Transcription products were purified on a Diethylaminoethyl (DEAE) Sephacel (GE Healthcare) column in 20 mM Tris-HCl pH 8.0, 5 mM MgCl_2_, and 250 mM NaCl. tRNA was eluted from the resin with 1 M NaCl. The RNA was precipitated overnight at -20°C in 1/10^th^ volume sodium acetate and 3x volume ethanol and re-suspended in RNase-free H_2_O.

### Marine natural product library

The marine natural products screening library comprises 5,304 fractions from organic extracts of marine-derived Actinobacterial fermentations (1 litre culture, following our standard protocol [59]). All fractions are stored as concentrated stock solutions in DMSO in standard 96-well format. The library is comprised of extracts of marine sediment-derived bacterial strains, containing a cross section of gram-positive genera and enriched in Actinobacterial strains, hand-collected from over 70 discrete dive sites on the West coast of the United States from the Channel Islands of Southern California to the San Juan Islands in Northern Washington.

Crude extracts were fractionated in to six sub-fractions on Seppak C_18_ cartridges using a stepwise elution profile (20, 40, 60, 80, 100% MeOH/ H_2_O, 100% EtOAc). The resulting fractions were solubilized in DMSO (1 mL per fraction), 4 μL aliquots diluted 1:5 in DMSO, and arrayed in 384 well format (17 x 384 well plates). The MNP library screened in this assay consisted of a focused group of bacterial extract pre-fractions that had already demonstrated activity against *Leishmania* in a prior whole cell assay against *L*. *donovani* amastigotes. The MNP library was also counter-screened in a mammalian system against HeLa cells [59]. Fractions with acute cell cytotoxicity were removed from the screening library, resulting in a set of test extracts with demonstrated activity against *L*. *donovani* and low/ no cytotoxicity against HeLa cells. Following primary screening against *L*. *donovani* amastigotes, 120 active fractions were arrayed as serial dilutions (8 x 2-fold dilutions; 50–0.4 μM) in 96 well format for aaRS screening.

### Screen for aminoacylation inhibitors

Serial dilutions from the marine natural product (MNP) library were screened using the following protocol. Aminoacylation reactions were performed at room temperature using 10 mM DTT, 8 mM ATP, 5 μM tRNA, 60–80 μM [^14^C]-Ala or [^14^C]-Thr, 100–500 nM aaRS, and DMSO or MNP samples. After incubating the reaction for either 15 or 20 minutes, 1 μL of the reaction was spotted on 5% pre-soaked TCA 3 MM Whatman filter paper. The precipitated tRNA-bound filter paper was washed 3x with 5% TCA, washed once with ethanol, and dried. The dried filter paper was exposed overnight on a phosphor imager screen and imaged the following day. Qualitatively, the phosphor image screen was examined for a change in signal intensity relative to the DMSO control; a decrease in phosphor image intensity indicates partial or full inhibition of the reaction in the presence of the inhibitor. While active concentrations were unknown for each of the MNP mixes, the serial dilution helped prevent false-positive identification. All lead candidates from the preliminary screen were confirmed using similar reaction conditions; the reactions were monitored over a time course and placed at 37°C. Samples were quantified using a scintillation counter.

### Pyrophosphate exchange

Amino acid activation was monitored using ATP/PP_i_ exchange as previously described [60]. Reactions were performed at 37°C in 100 mM HEPES pH 7.2, 30 mM KCl, 10 mM MgCl_2_, 2 mM NaF, 2 mM ATP, 2 mM [^32^P]-PP_i_ (Perkin Elmer), 90 μM alanine, 160 nM AlaRS, and DMSO or aaRS inhibitor. At increasing time points, aliquots of the reaction mixture were quenched in a charcoal solution containing 1% activated charcoal, 5.6% HClO_4_, and 75 mM PP_i_. Quenched reactions were vacuum filtered on to 3MM Whatman filter discs, washed three times with 5 mL of water and once with 5 mL of ethanol. After drying the filter discs, charcoal-bound radiolabeled ATP was quantified on a scintillation counter. Relative endpoint amino acid activation was determined by comparing the inhibitor-treated enzymes to their respective DMSO control samples.

## Results

### Custom annotation of tRNA genes and gene clusters in TriTrypDB genomes

We obtained 4381 unified gene records from the raw output of two tRNA gene-finders, Aragorn and tRNAscan-SE v.2.0, to TriTrypDB v.41. Of these, 3597 were found by both gene-finders, 750 were found by Aragorn only, and 34 were found by tRNAscan-SE 2.0.0 only. We identified the same 76 genes as initiator tRNA genes, using either tRNAscan-SE 2.0.0’s profile-based predictions or our own edit-distance-based clustering approach, by finding the unique set of genes carrying conserved initiator tRNA features as described in [42].

**Fig 1:**
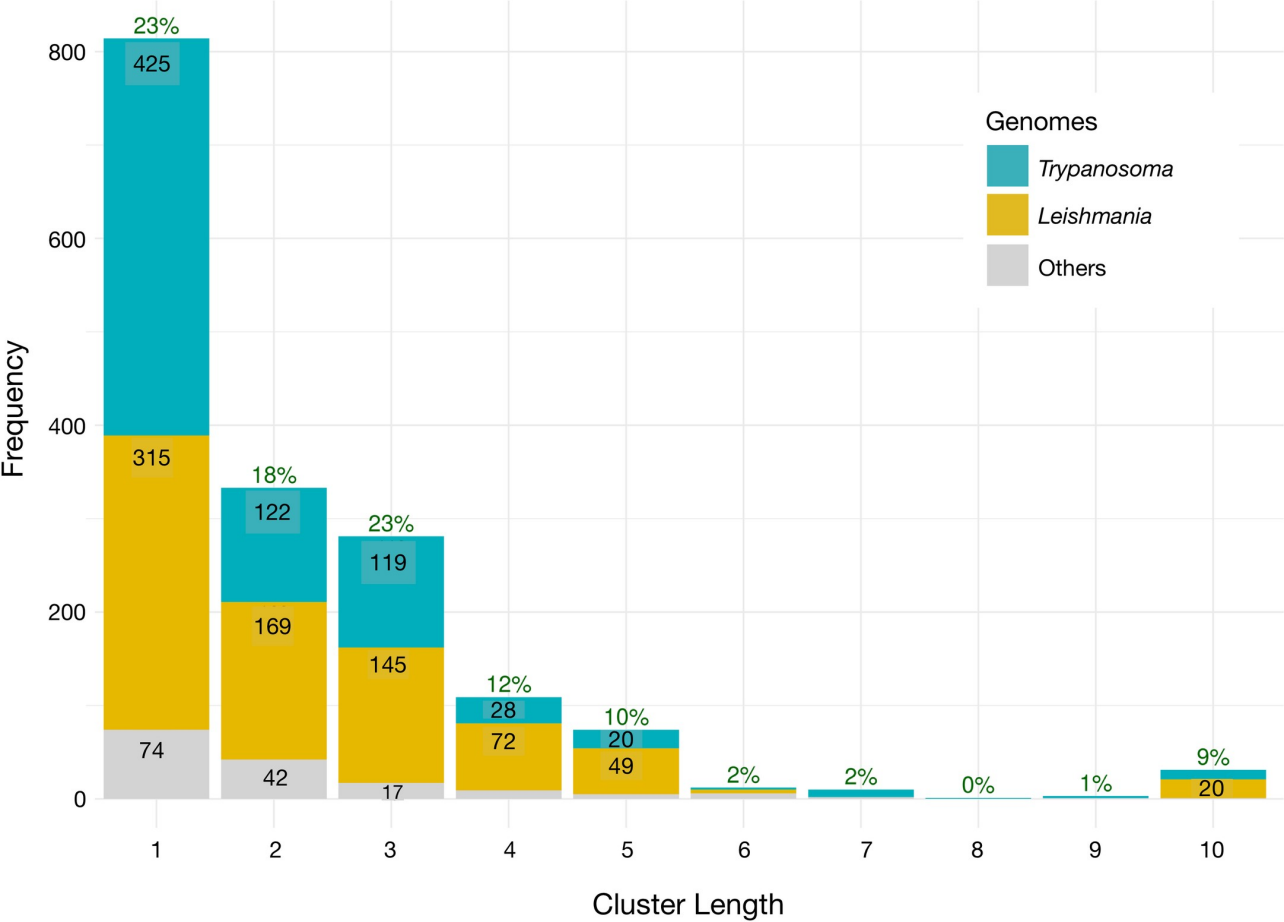
tRNA gene cluster size distribution for *Trypanosoma*, *Leishmania*, and other TriTrypDB version 41 genomes. Green labels at tops of stacks show percentages of total tRNA genes in clusters of given length. Numbers within each bar show frequencies of gene clusters of that length.

To further refine the final annotated gene set, we identified tRNA gene clusters in TriTrypDB genomes using a maximum intergenic distance criterion of 1000 bp on either strand. Doubling this distance criterion did not substantially increase cluster number or size. After filtering 4381 gene records by their gene-finder scores as described below, 3616 high-confidence gene records remained, of which 77% occur in clusters of size two or greater (Fig 1). The largest tRNA gene clusters were of size ten, accounting for 9% of total genes. We used Jaccard distance as a gene functional content distance to hierarchically cluster tRNA gene clusters with similar gene functional contents, and found that distance cutoffs between 0.680 and 0.692 defined intuitively reasonable similar, distinct, and putatively homologous tRNA gene cluster variant groups that we found to be conserved either within each of the *Leishmania* and *Trypanosoma* genera, or across both genera, with substantial evidence of evolution in gene organization and content of gene clusters within groups through gene duplication, divergence, inversion and other changes (S1–S3 Tables). Some evidence for whole tRNA gene cluster duplication and paralogy within our gene cluster variant groups (labeling variants by the concatenation of their tRNA gene function / aminoacylation identities in gene order, using IUPAC one-letter-codes for amino acids to stand for identities) include one gene cluster variant DSA, with a frequency of three in the genome of *T*. *cruzi* DM28c and a frequency of two in the genome of *T*. *cruzi* DM28c, and nine other cluster variants with frequencies of two in one or more genomes, including tRNA gene cluster variants ASD, FEV, NARK, and VYMEMSL occurring twice in the genome of *T*. *cruzi* Tulacl2, variants PTN and VEF occurring twice in the genome of *T*. *cruzi* DM28c, variant EVRH occurring twice in *L*. *arabica* LEM1108, variant FEV occurring twice in *T*. *cruzi* SylvioX10-1, variant GAL in *L*. *tropica* L590, and variant HEF in *T*. *congolense* IL3000. The conservation of tRNA gene cluster variants and groups spanning TriTrypDB genome assemblies of different genera is indisputable, but further statistical and phylogenetic characterization of them may best be undertaken via long-read genome resequencing, as tRNA gene clusters can be difficult to assemble reliably from short-read sequencing data.

**Fig 2:**
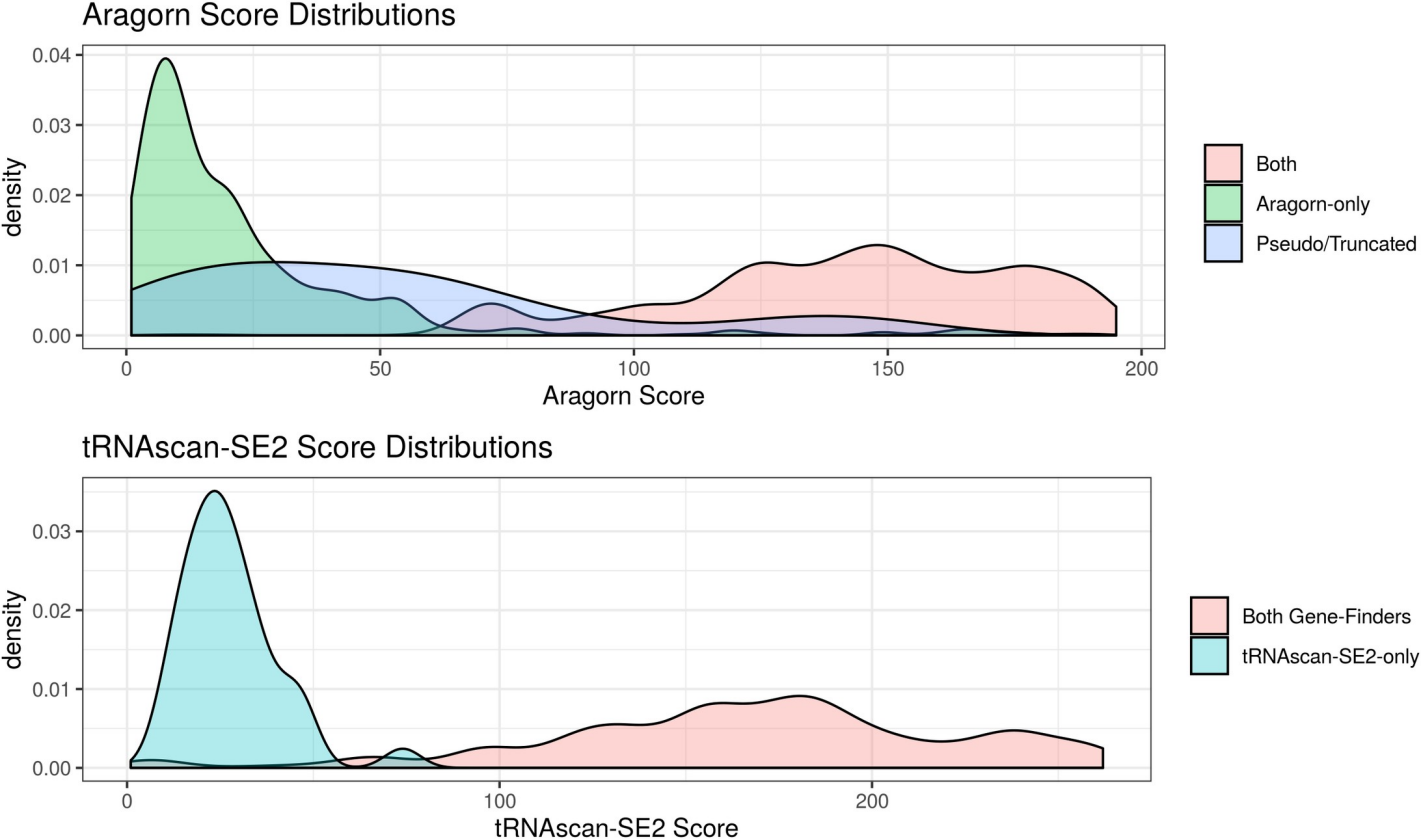
Density plots of gene-finder scores according to source of detection.

Using the similarities of tRNA gene clusters across genomes, we found putative homologs for some of the 45 functionally ambiguous but high-scoring genes marked as pseudogenes or truncated by tRNAscan-SE 2.0.0 as well as some genes detected only by Aragorn. With these results in mind, we plotted the densities of gene-finder scores according to whether they were found by both or only one gene-finder, showing clear evidence of a small fraction of Aragorn-only genes with high scores, making up about 1% of our total finalized gene set (Fig 2 and S4 Table).

Based on this evidence, we retained 3616 genes from 46 TriTrypDB genome assemblies that had an Aragorn bit-score of at least 107 or a tRNAscan-SE 2.0 bit-score of at least 50, including 36 genes found by Aragorn only and 1 gene found by tRNAscan-SE 2.0 only. These score cutoffs separated Aragorn-only genes within conserved gene clusters from singletons, which had lower scores (S1 Fig). At time of publication, a more recent version of tRNAscan-SE, 2.0.5, could find 24 of the 36 high-scoring ara-only genes in our union set. The 24 additionally found genes were identical or had only 1 or 2 base differences in sequence to other high-scoring genes in our defined intersection set. However, we observed them to lie either near the ends or close to ambiguous segments (strings of Ns) in the genome assembly sequences.

The median number of genes per genome in our raw union annotation gene set was 82. Among these were 45 functionally ambiguous but high scoring genes, including 2 with identity unassigned by both gene-finders, 6 marked as pseudogenes or truncated by tRNAscan-SE 2.0.0, 4 containing sequence ambiguities, and 33 with conflicting structural and anticodon annotations, including ten intron-containing genes predicted as tRNA^Tyr^ genes by tRNAscan-SE and tRNA^Asn^ genes by Aragorn, all from genomes in the American *Trypanosoma* clade. We annotated these ten as tRNA^Tyr^ genes following tRNAscan-SE 2.0.0, which are known to contain introns in that clade [61], as this helped complete the sets of functional types for 8 genomes in that clade (S2 Fig). After score filtering, three genome assemblies that were not excluded from further analysis remained incompletely annotated (S2 Fig): *T*. *Cruzi* dm28c (missing a gene for tRNA^Phe^), *T*. *congolense* IL3000 (missing genes for tRNA^Asp^ and tRNA^Ser^) and *T*. *vivax* Y486 (missing a gene for tRNA^Tyr^). Candidate genes were available in our raw union annotation gene-set to complement some but not all of the 46 genomic gene sets that were missing classes (S2 Fig), including one Aragorn-only candidate gene of 100 bits for tRNA^Tyr^ in *T*. *cruzi* MarinkelleiB7, between 5–8 low- or marginal-scoring genes for tRNA^Ser^ in *T*. *congolense* IL3000 (3 from tRNAscan-SE2 with bit scores between 22 and 25 and 5 potentially overlapping genes predicted from Aragorn with scores between 100 and 106), a tRNAscan-SE2-only candidate gene for tRNA^Tyr^ scoring 36 bits in *T*. *vivax* Y486, and an Aragorn-only candidate gene for tRNA^Asp^ scoring 100 bits in *P*. *confusum*. S4 Table shows structural and functional statistics on our score-filtered annotation gene sets. Our score-filtered union annotation gene-set was further filtered and pooled into defined clade gene-sets as described in the Methods section. Table 1 shows mean and standard deviations of tRNA gene number and pooled composition statistics by clade. Every clade contained genes for all 21 functional types excluding tRNA^SeC^ (Table 1 and S2 Fig). S5 Table gives gene numbers and compositions of gene sets by individual genome. Table 1 and S5 Table show that the overall divergence in gene compositions is not great, and follows phylogenetic expectations based on phylogeny. The gene compositions of the *Leishmania* clades are quite similar, and different from those of both humans and *Trypanosoma*. Human gene composition is most divergent, with African *Trypanosoma* gene composition second most divergent from those of *Leishmania*. American and African *Trypanosoma* compositions are less divergent from each other. However, *Trypanosoma* and human tRNA gene compositions are different from those of *Leishmania* in different ways. Human tRNA genes are richer in purines while *Trypanosoma* tRNA genes are richer in G and C. American *Trypanosoma* are also GC-rich, but less so than African *Trypanosoma*. S5 Table shows that there is little heterogeneity of gene set compositions by genome assembly within clades, with the greatest variation appearing in *Trypanosoma*, particularly African *Trypanosoma*.

### Divergent class-informative features between humans and TriTrypDB genomes

**Fig 3:**
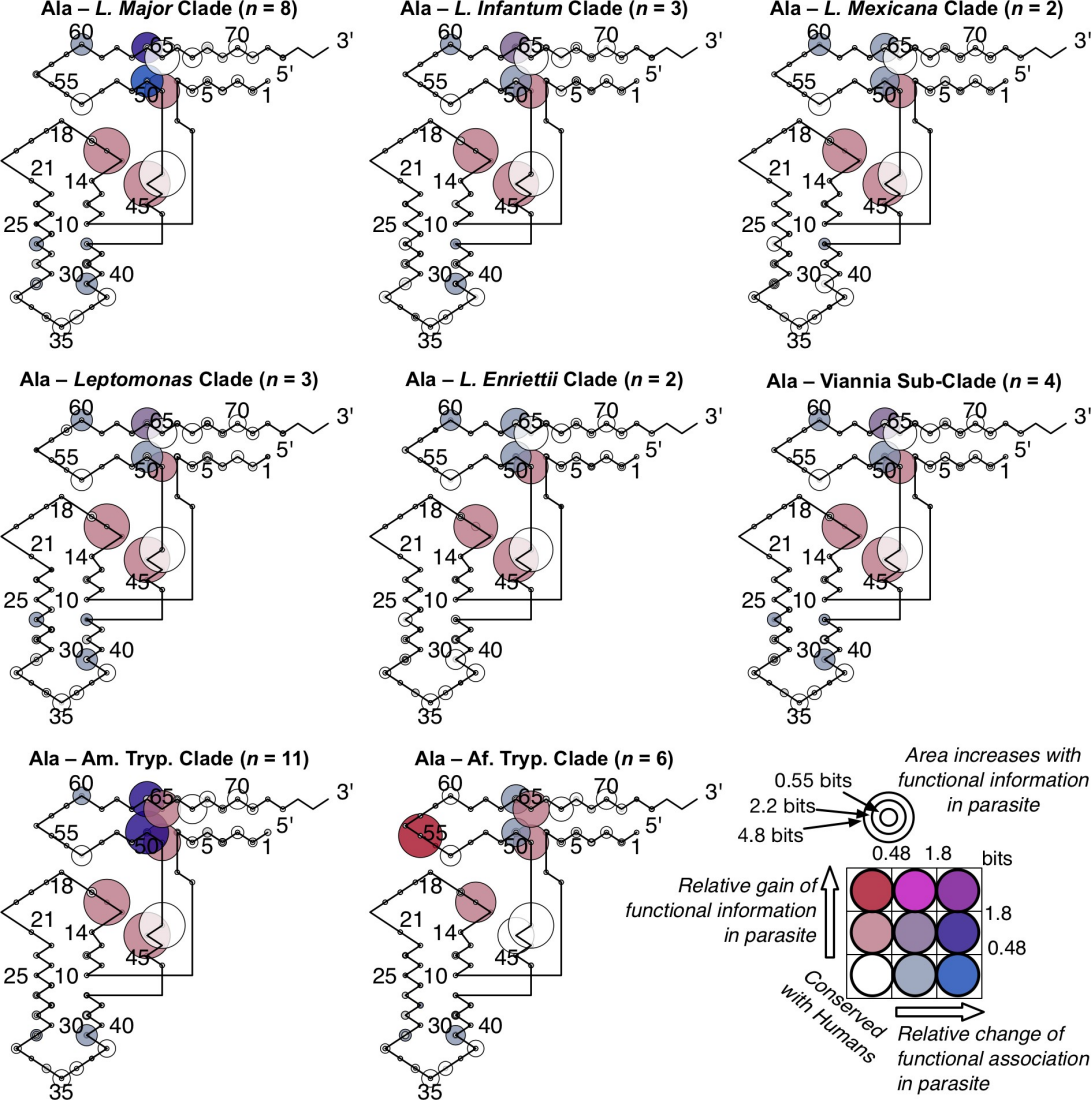
Conserved divergence of parasite tRNA^Ala^ CIFs across eight phylogenetic clades of *Leishmania* and *Trypanosoma*. Evolutionary divergence of trypanosomes relative to *Leishmania major* increases clockwise from *Leishmania major*.

**Fig 4:**
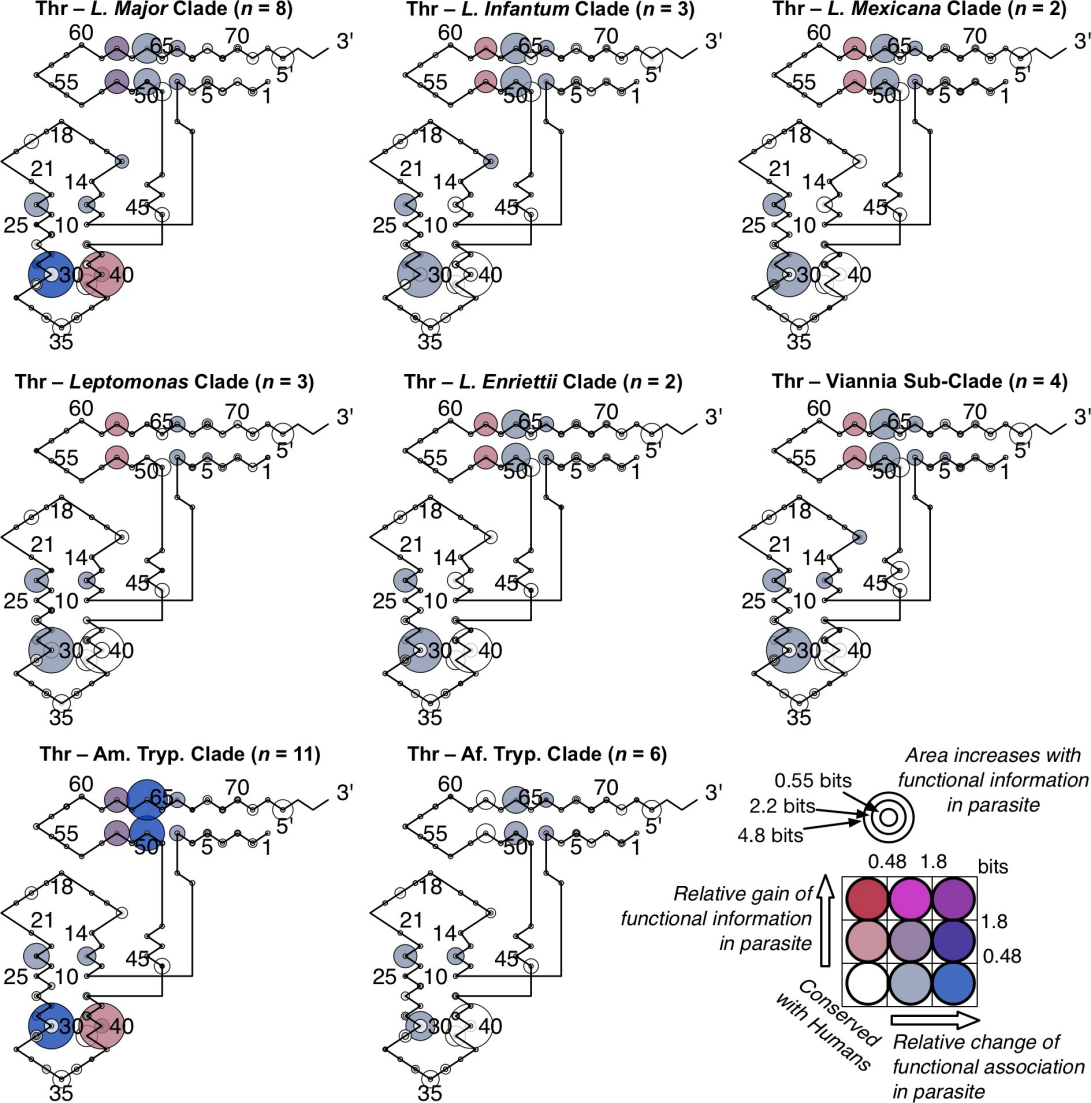
Conserved divergence of parasite tRNA^Thr^ CIFs across eight phylogenetic clades of *Leishmania* and *Trypanosoma*. Evolutionary divergence of trypanosomes relative to *Leishmania major* increases clockwise from *Leishmania major*.

**Fig 5:**
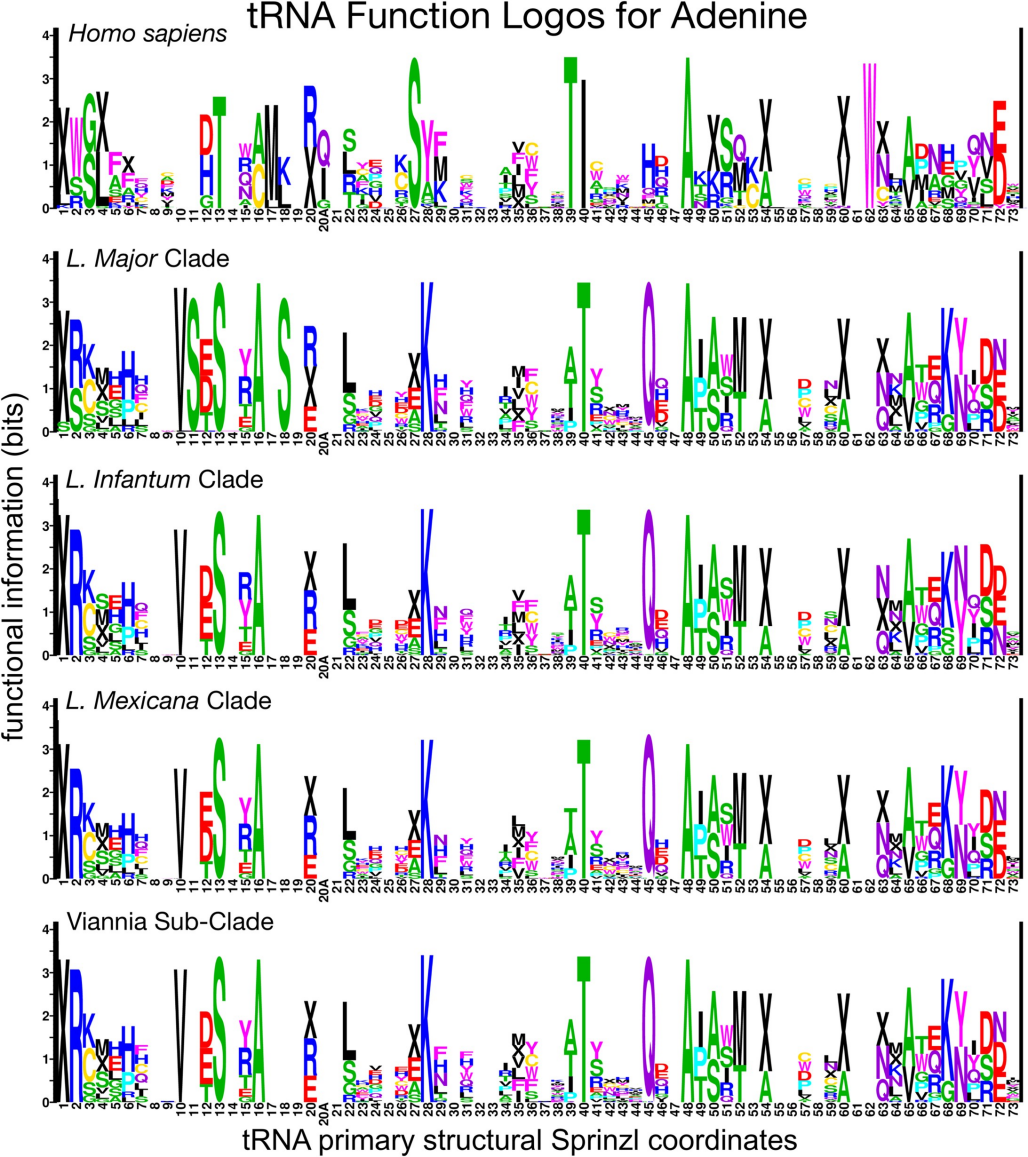
Adenine function logos for humans and four clades of *Leishmania*. Complete single-site function logo results are shown in S24–S31 Figs. The total height of a stack of letters at any site quantifies the information potentially gained about the functional type of a tRNA by a tRNA-binding protein if it recognizes the specific feature corresponding to that site and logo, for example Adenine at Sprinzl coordinate 16 (or some modification that biosynthetically depends on A16) in the case of the left-most boxed site. The letters within each stack symbolize functional types of tRNAs, wherein IUPAC one-letter amino acid codes represent elongator tRNA aminoacylation identities and “X” symbolizes initiator tRNAs. The relative heights of letters within each stack quantifies the over-representation of tRNA functional types carrying that feature relative to the background frequency determined by gene frequencies of functional types (as calculated through the normalized log-odds).

**Fig 6:**
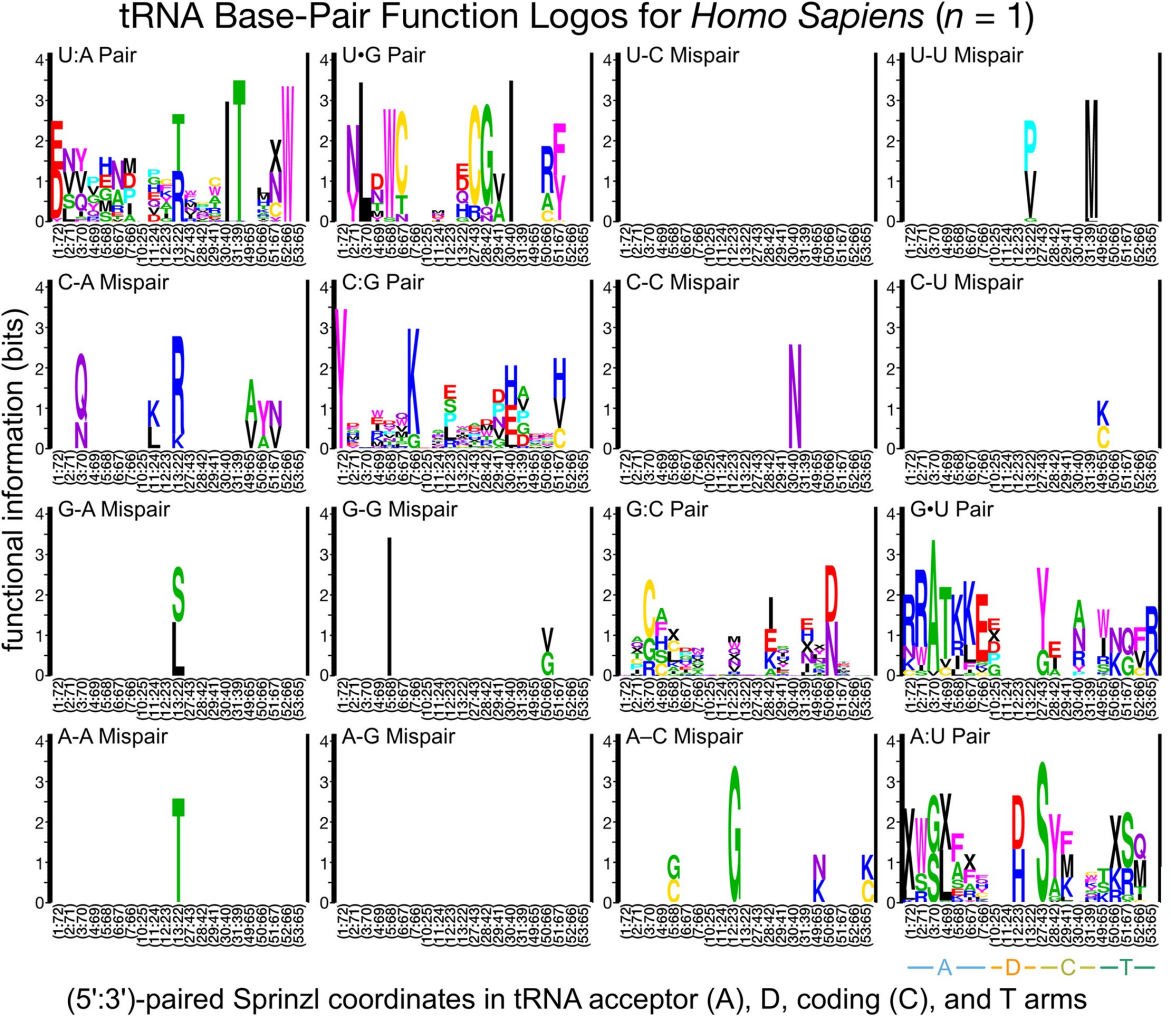
Function logos for tRNA Class-Informative Base-Pairs and Class-Informative Mis-Pairs in humans. The meanings of letters, stack heights and letter heights are all the same as in Fig 5.

**Fig 7:**
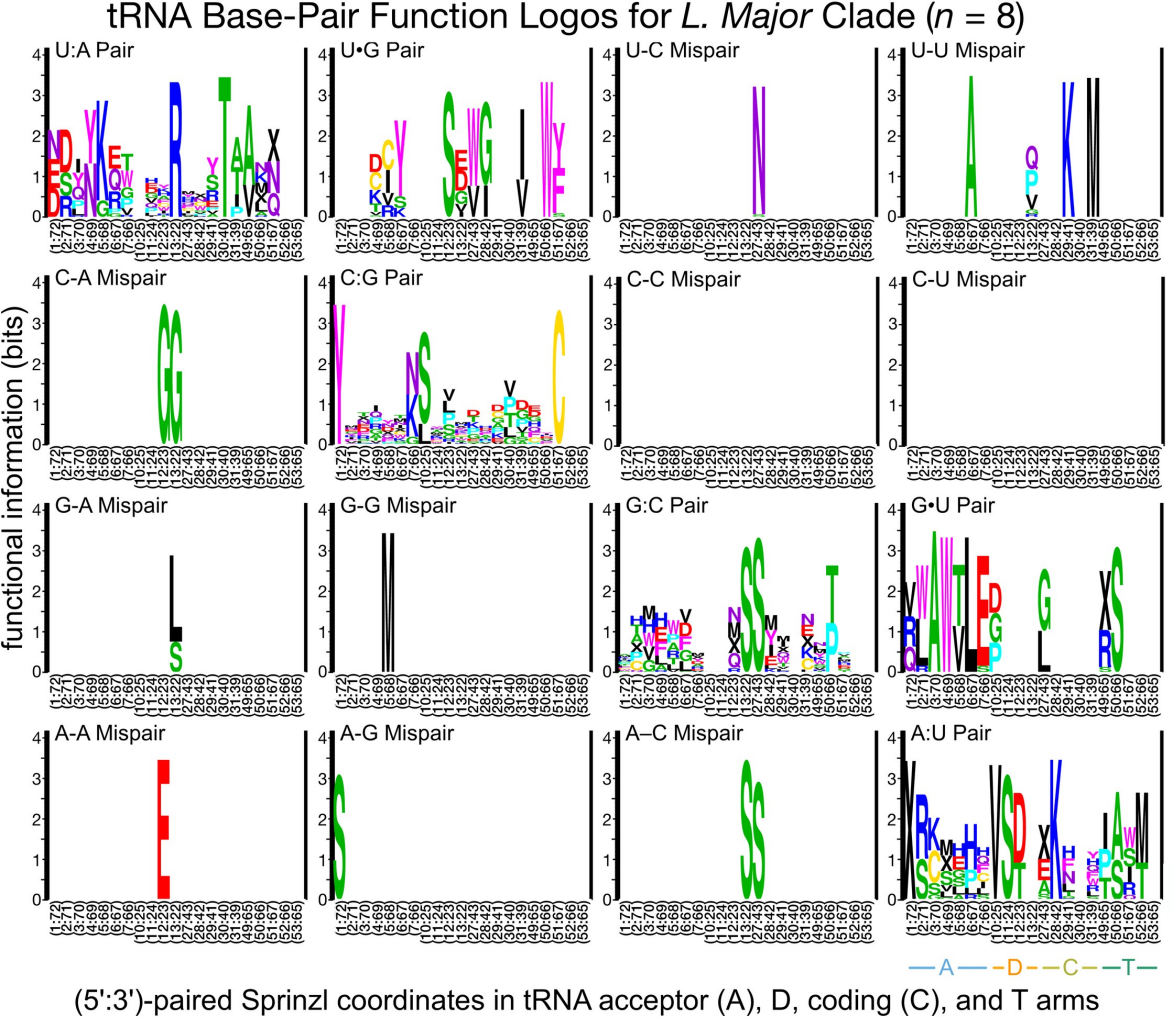
Function logos for tRNA Class-Informative Base-Pairs and Class-Informative Mis-Pairs in the *L*. *major* clade. The meanings of letters, stack heights and letter heights are all the same as in Fig 5.

**Fig 8:**
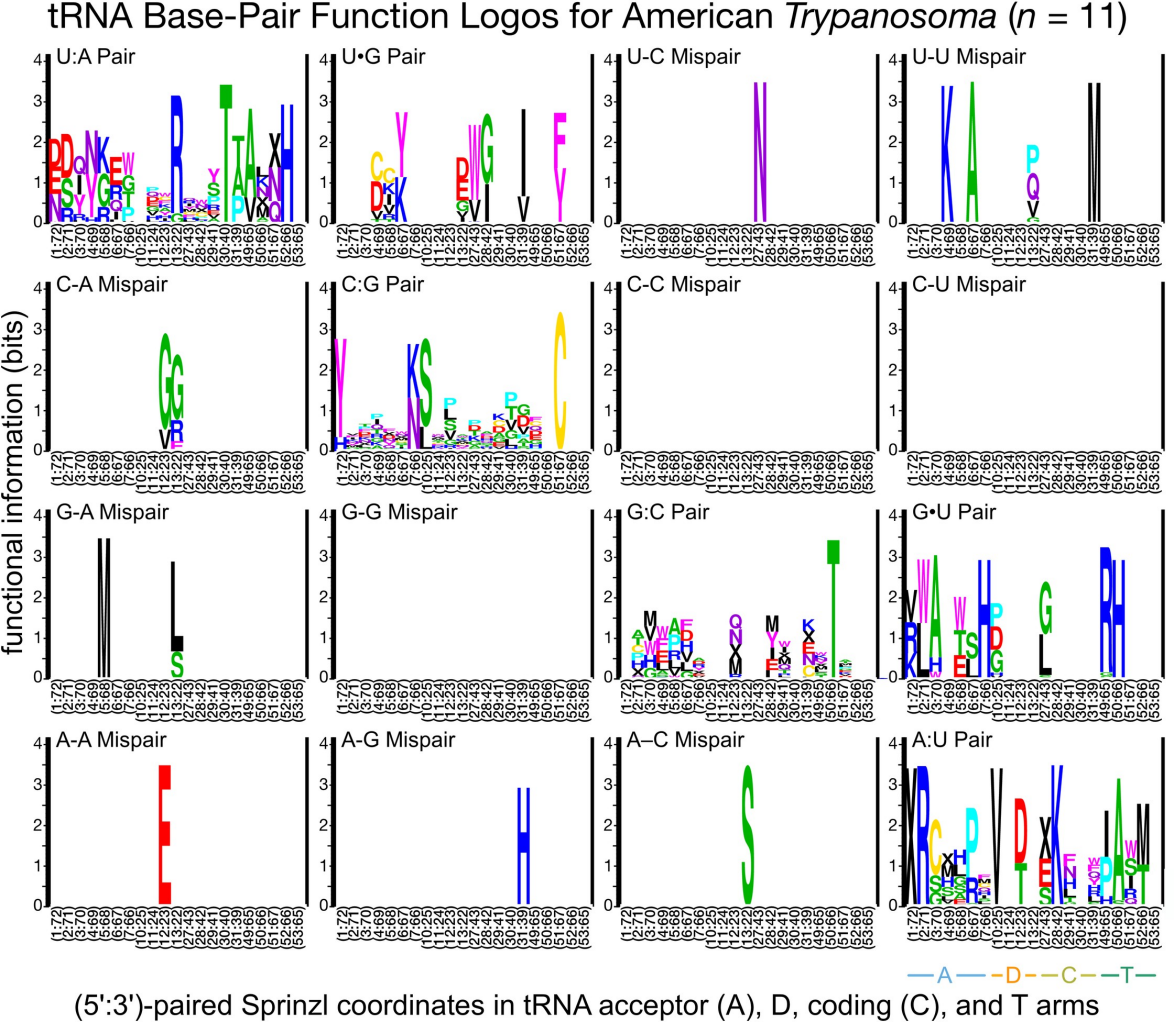
Function logos for tRNA Class-Informative Base-Pairs and Class-Informative Mis-Pairs in the American *Trypanosoma* clade. The meanings of letters, stack heights and letter heights are all the same as in Fig 5.

We developed a bioinformatic workflow that combines information from tRNA function logos estimated from a parasite clade and Information Difference (ID) logos [25] and Kullback-Leibler Divergence (KLD) logos between the parasite clade and humans [27]. The workflow quantitates tRNA features that are functionally informative in the parasite clade and have either gained or retained functional information or altered functional association since divergence of the parasite clade and humans from their common ancestor. We found many examples of highly informative trypanosome CIFs that have been gained, retained or changed functional information since divergence from their common ancestor with humans, and most of these divergent CIFs have been strongly conserved in trypanosomes over 231–283 million years of evolutionary divergence between *Leishmania* and *Trypanosoma* [62], for example among alanine tRNAs (Fig 3) and threonine tRNAs (Fig 4). Structural bubble-plot visualizations at single-site resolution of these CIF divergence measures are provided for all functional classes in supplementary materials, showing that some classes have diverged much more than others. Even though they are calculated at single-site resolution, CIF divergences are correlated across structurally paired sites. Inspection of singles-ite function logos across taxa confirms the conservation of parasite-specific CIFs and reveals A- and U-containing features underlying the signals shown in Figs 3 and 4, including some sharing of divergent features between tRNA^Ala^ and tRNA^Thr^ functional classes, for example at Sprinzl coordinate 39 (Figs 5 and S24–S27). Figs 6–8 show base-pair function logos for Humans, the *L*. *major* clade and the American *Trypanosoma* respectively, showing that both Class Informative Base-Pairs and Class-Informative Mis-Pairs can be relatively conserved, and that recurring hot-spots of CIF evolution appear in the data, yielding insight to mechanisms of CIF evolution. Inspection of Class-Informative Base-Pairs and Mis-Pairs shows that a U:A informative base-pair diverged in tRNA^Thr^ to an adjacent site-pair, from 31:39 to 30:40, and that a U-U informative mispair was gained in tRNA^Ala^ at site-pair 6:66 in trypanosomes relative to humans (Figs 6–8). Full function logo results for all clades are provided in S24–S40 Figs.

Our computational screen for tRNA CIF divergence as shown in S3–S23 Figs, show that tRNA^Ala^ and tRNA^Thr^, are among those tRNA functional types that have the greatest number of sites and site-pairs with the largest CIF divergence relative to humans and would be good potential candidates for therapeutic targeting. Contrast, for example, our results for Trypanosome tRNA^Tyr^ or tRNA^Trp^, which show trypanosomal tRNA CIFs that are strongly conserved with humans, as shown in S9 or S10 Figs. These observations led us to follow-up and investigate tRNA^Ala^ and tRNA^Thr^ because both these tRNA types, and their accompanying cognate synthetases, are readily reconstituted *in vitro*. Previous experience with *in vitro* reconstitution of AlaRS and ThrRS enzymes in the Ibba lab aided us in troubleshooting problems and avoiding false-positive identification of inhibitors, as artifactual changes in enzyme activity are common if technical care is not taken.

### AaRS screen identified *Leishmania major* AlaRS inhibitors

**Fig 9:**
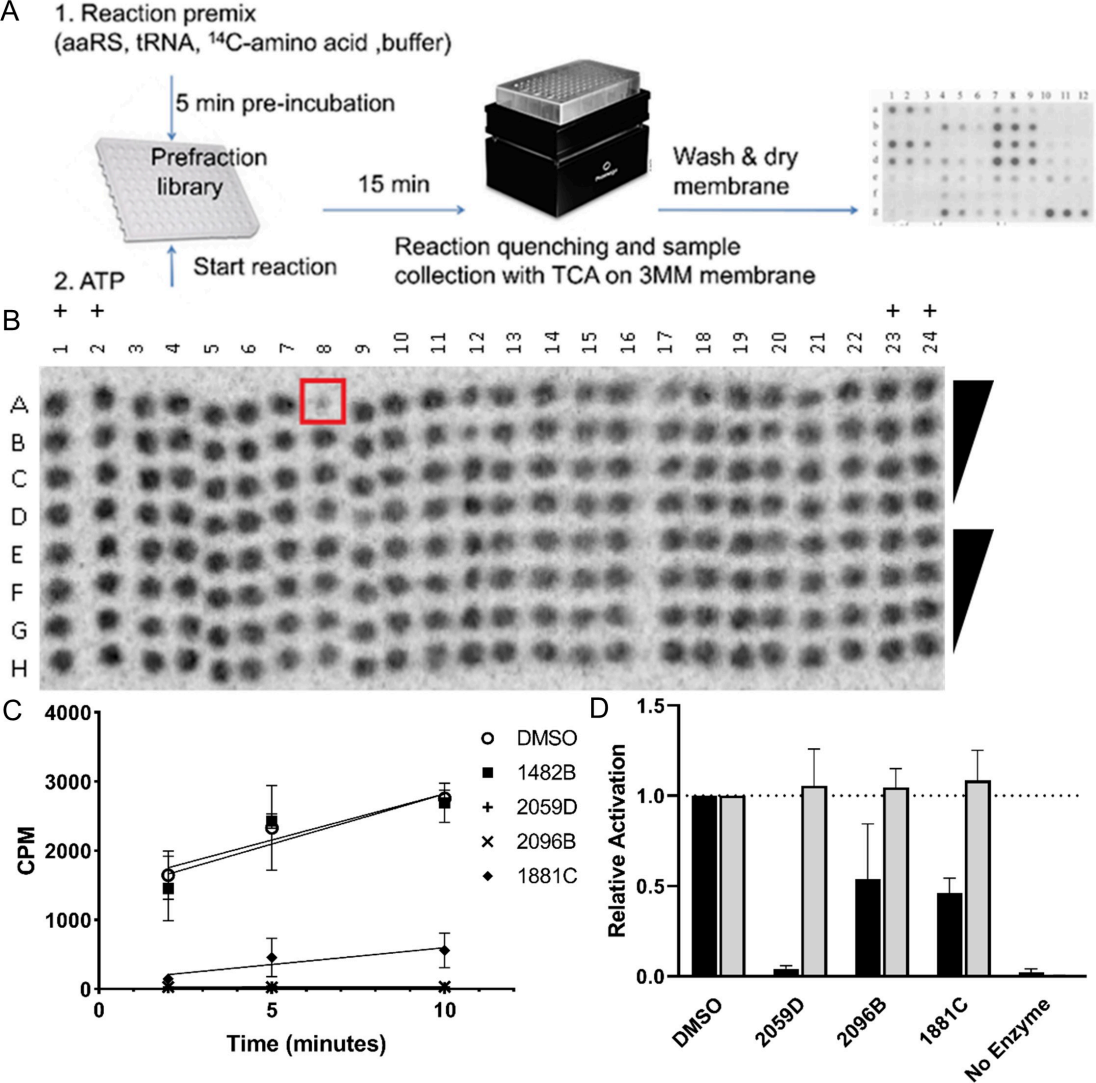
Identification of *Leishmania major* AlaRS inhibitors. A) Workflow to identify aminoacylation inhibitors (details described in Methods). B) Representative image of the MNP chemical screen. The spot boxed in red is an example of a predicted inhibitor depicted by the decrease in signal intensity. DMSO positive control (+). C) Three of the four identified inhibitors prevented the accumulation of Ala-tRNA^Ala^ formation, 1428B was a false-positive result from our preliminary screen. D) The three identified inhibitors perturbed *L*. *major* AlaRS activation (black) but had no effect on human AlaRS (gray). The relative amino acid activation is plotted relative to the DMSO control. Error bars indicate the standard deviation of three replicates.

Using the pre-validated MNP library, we developed a medium-throughput phosphorimaging-based aminoacylation screen to identify aaRS inhibitors *in vitro* (Fig 9A and 9B). From the one hundred and twenty complex inhibitory mixes tested in the MNP library, we qualitatively identified four potential *Lm* AlaRS inhibitors as determined by a decrease in the overall tRNA-aminoacylation signal. These four candidates were then re-screened using time-dependent quantitative approaches and we concluded that three of the four mixes, 1881C, 2059D, and 2096B were altering aminoacylation, with inhibitory activities ranging between 80% and 99% (Fig 9C). Using means and standard deviations of four replicate scintillation count-per-minute endpoints under the DMSO control condition or without added enzyme, we calculated an acceptable Z-factor of 0.67 for this follow-up aminoacylation time-course-based assay (S6 Table).

Since the aminoacylation screen discerns total net changes to the aaRS activity, we attempted to identify which step of the aaRS catalyzed reaction is being affected by the MNPs. To observe any tRNA-independent effects on aaRS function, we used pyrophosphate exchange to monitor ATP-dependent amino acid activation. From this experiment, we were able to conclude that our inhibitors were perturbing amino acid activation, with lead compounds ranging in inhibitory activity between 45% and 95%. The differences in MNP activity between amino acid activation and tRNA-dependent aminoacylation highlight the multiple aaRS activities that can be targeted in our network predictions. To validate the predictive tool for identifying anti-trypanosomal drugs, we counter-screened the newly identified *Lm*. AlaRS inhibitors against the human AlaRS enzyme. Treatment of the human AlaRS enzyme with the MNP inhibitors had no effect on amino acid activation (Fig 9D). Combined with our original screening data, these results show the utility of our computational and biochemical workflow to identify new novel therapeutics that have minimal cross-reactivity with the human homolog of the parasite drug target.

### Natural product library inhibitors of *Leishmania major* ThrRS

**Fig 10:**
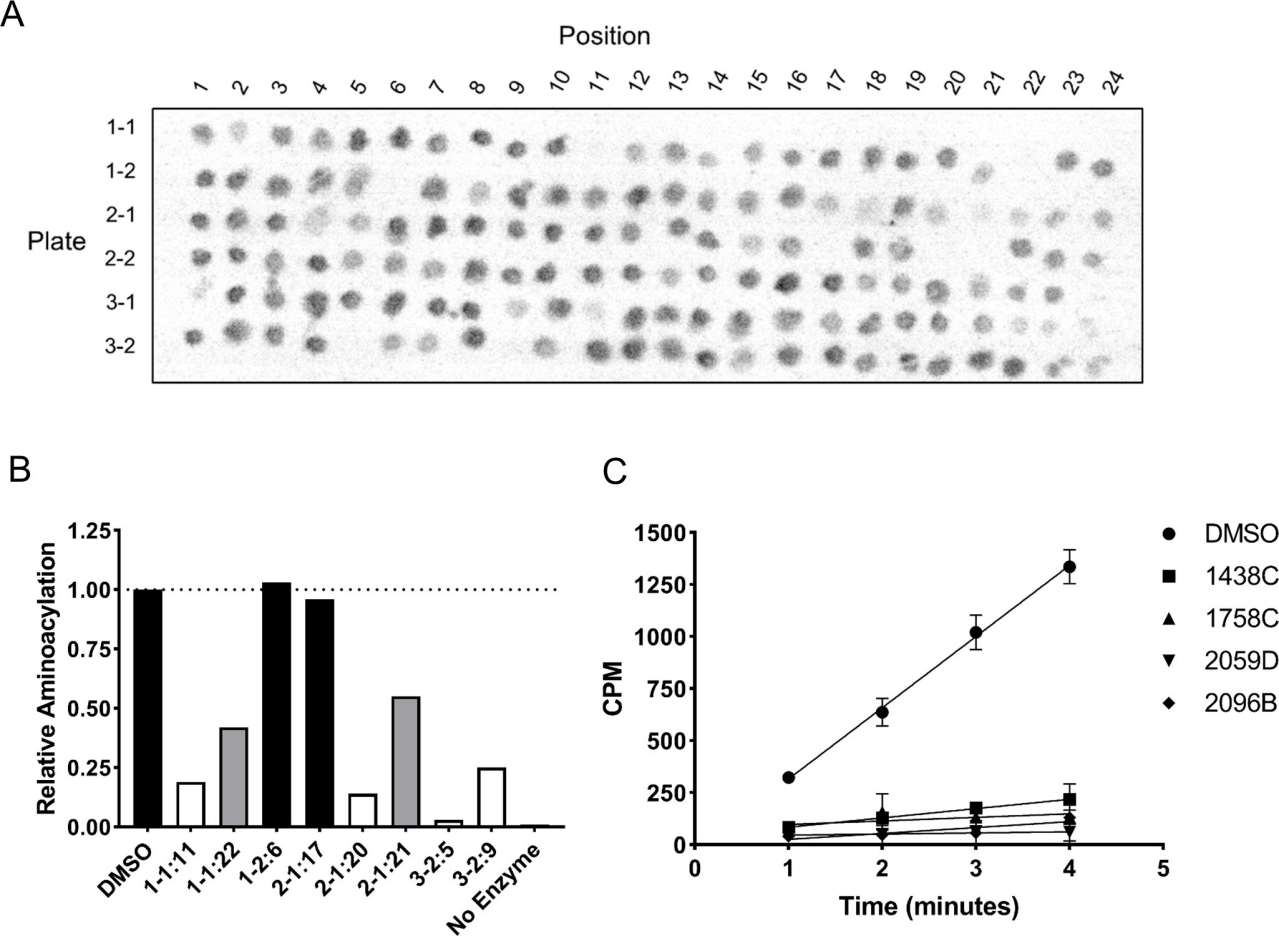
Identification of *Leishmania major* ThrRS inhibitors. A) The MNP library was re-screened at the highest concentrations to qualitatively identify *Lm* ThrRS aminoacylation inhibitors. Plate IDs reference the position within the original library and not library IDs. B) Eight inhibitors were qualitatively identified from the preliminary screen. Two of the candidates did not inhibit aminoacylation (black), two inhibited at ~50% activity (gray), and four inhibited at greater than 25% (white).C) All four active inhibitors continued to perturb aminoacylation over a time course experiment. Error bars indicate the standard deviation of three replicates.

As our network predictions identified CIF divergence among many functional classes of tRNAs (as shown in the Supplementary materials), we also wanted to determine if our MNP library would find inhibitors against non-AlaRS aaRS. The most concentrated mixes from our MNP library were re-screened against *Lm* Threonyl-tRNA synthetase (ThrRS) and tRNA^Thr^ aminoacylation (Fig 10A). The preliminary screen led to the identification of eight extracts with inhibitory activity. Those fractions were re-analyzed using quantitative aminoacylation reactions and the results show that that two of the candidates did not inhibit aminoacylation, two inhibited the reaction at ~50%, and four had greater than 75% inhibition (Fig 10B). In addition, two of the four most active inhibitors (2059D and 2096B) also had activity against *Lm* AlaRS (Fig 10C). The cross-reactivity of these inhibitors may be a consequence of the extensively conserved aaRS architecture found between AlaRS and ThrRS [63,64].

### Predictive network interactions identified broad-spectrum anti-trypanosomal targets

**Fig 11:**
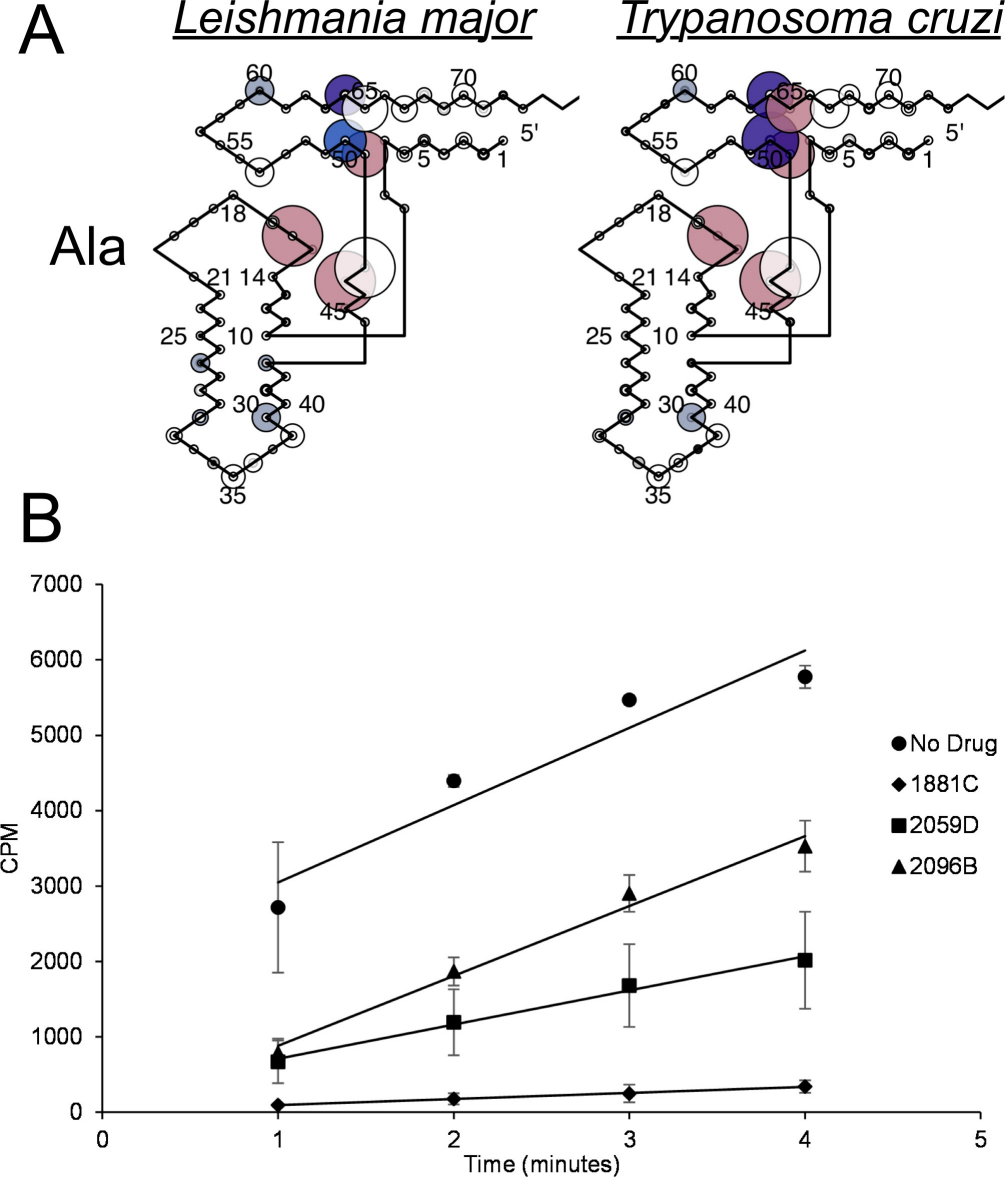
*Leishmania major* and *Trypanosoma cruzi* AlaRS have conserved tRNA identity elements. A) CIF Divergence Models for tRNA^Ala^ in Leishmania *major* and *Trypanosoma cruzi* B) The three identified *Lm* AlaRS inhibitors also have activity against the *Tc* AlaRS enzyme. Error bars indicate the standard deviation of three replicates.

The tRNA-aaRS network analyses suggested that parasite-specific tRNA^Ala^ identity elements were highly conserved between the *Leishmania* and *Trypanosoma* genera (Figs 3,4,5 and 11A). To test this hypothesis, we purified *Tc* AlaRS and screened our three active *Lm* AlaRS inhibitors in an aminoacylation inhibition assay using *Tc* AlaRS and tRNA^Ala^. Supporting our network prediction, all three *Lm* inhibitors also had activity against the *Tc* enzyme, with activities ranging between 40% and 95% total inhibition (Fig 11B). While these activities were slightly reduced compared to their effect of the *Lm* AlaRS enzyme (Fig 9C), these results highlight the additional potential utility of our computational methodologies as a means of identifying broad-spectrum antimicrobials for closely-related clades.

### Separation of active components from natural products extracts

From the initial set of 120 extracts with activity against *L*. *donovani* parasites, four extracts showed corresponding activity in the initial aaRS assay. Of these, three (1881C, 2059D, and 2096B) were prioritized for chemical follow up, based on potent, dose-dependent biological activity. Initially, each sample was separated into 10 sub-fractions using HPLC (Phenomenex Synergy C18, 5μ, 4.6 x 250 mm). Screening of these fractions identified one fraction (2096B F10) with potent activity. To generate additional material, the producing organism was cultured on large scale (1 L, GNZ medium with 20 g XAD-7 resin), filtered, and the resin/cell slurry extracted with organic solvents (2:1 CH_2_Cl_2_/ MeOH, 400 mL). The crude extract was fractionated using an automated Combiflash chromatography system (C_18_ cartridge; 20, 40, 60, 80, 100% MeOH/ H_2_O, 100% EtOAc) and the resulting fractions subjected to biological screening (S1 Text). Two fractions (C and D) showed strong activity and were subjected to subsequent separation to give 10 sub-fractions each (S41–S44 Figs). Of these, fraction 2096D F10 showed the strongest reproducible activity (S45 Fig). However, subsequent fractionation steps yielded sub-fractions with very low quantities of material. Review of these sub-fractions by UPLC-ESI-qTOF mass spectrometry did not identify any individual mass signatures consistent with a candidate bioactive molecule. Similarly, lack of material precluded the identification of diagnostic signals in the NMR spectra for these subfractions. Provisional information from these analyses, including NMR and MS signatures from earlier fractions and the non-polar nature of the active fractions, suggest that the active component is likely a bioactive lipid, although the precise nature of the structure of this metabolite remains unknown. The isolate producing the bioactive substance was collected on April 20th, 2012 from marine sediment off the coast of Kellet Bluff, Henry Island, WA US under the permit issuing authority of the Washington Department of Fish and Wildlife (permit # 12–034).

## Discussion

### Systems-biology driven identification of trypanosome-specific drug targets

tRNA CIFs apply an information criterion using function logos, rather than a conservation criterion using conventional sequence logos, to bioinformatically predict tRNA identity elements. Even though we did not apply a conservation criterion in our predictions, when we applied our information criterion independently over different trypanosome clades, we found that tRNA CIFs were highly conserved over 250 million years of trypanosome evolution. A biological interpretation of this result of tRNA CIF conservation within trypanosomes (and also between trypanosomes and humans) is that the information contained in tRNA CIFs is functional in specifying substrate identity to tRNA-binding proteins such as aaRSs. That is to say, tRNA-binding proteins themselves exploit the information contained in tRNA CIFs to identify their tRNA substrates against the background of all possible tRNAs, with which they must interact to varying degrees. We present a systems biological theory for the function and divergence of tRNA CIFs in [24].

Maintaining efficient and accurate translation is predicated on catalytically productive interactions between aaRSs and free tRNAs in the cell. While the major identity elements for a given aaRS-tRNA pair are generally conserved, here we have identified divergent features within tRNAs that apparently contribute to divergent RNA-protein interactions in trypanosomes. Much of the focus in this work was on the phylogenetic divergence of identity elements among alanine tRNAs. This class of tRNAs strongly support the utility of our computational analyses as the tRNA^Ala^ identity elements have been one of the most well characterized to date [13,14]. Interestingly, it was recently shown that the conserved G3:U70 base pair is recognized by AlaRS using three distinct mechanisms across all domains of life [15]. This observation highlights that even highly conserved identity elements may be recognized and discriminated against by distinct biophysical aaRS interactions, which may therefore be stronger potentially specific drug targets than previously anticipated. The dominant association of G3:U70 with tRNA^Ala^ is conserved among all trypanosome clades and humans in our data (Figs 6–8 and S32–S40 Figs).

The primary objective of this research was to develop a computational workflow to quantify divergence of functionally informative features of tRNAs across different evolutionary clades. The practical application of this work is to use the information gained from our computational analyses to identify novel therapeutic targets that may be of use in the clinic. As described above, tRNA^Ala^ and tRNA^Thr^ were specifically chosen because of their amenability for *in vitro* reconstitution, while the computational results shown in Supplementary Figures show that other leishmanial tRNA/aaRS pairs could serve as additional therapeutic targets either using our MNP library or other available libraries. While interesting, those discoveries are outside the scope of the present work and left to future investigations.

### Inhibition of aminoacyl-tRNA synthetases

A goal of this work was to identify divergent tRNA identity elements in trypanosome parasites. We predicted that parasite-specific tRNA-aaRS interactions would be identified, sufficiently divergent from homologous human machinery to be strong candidates for drug discovery. Interestingly, our network divergence analysis led to the discovery of tRNA-independent, amino acid activation inhibitors that were specific to trypanosomes. We interpret this as consistent with our goal, because tRNAs and aaRSs must coevolve to accommodate changes to structure and mechanism that evolve on either side of their interactions. Presumably, divergence in the structural mechanism of amino acid activation in trypanosome AlaRSs has also changed how they interact with their tRNA substrates. By integrating information from many tRNA functional classes, we gain leverage to interpret divergence in structure and function of the much more structurally complex ensemble of aaRSs as a system. Our tRNA-based network approach identifies potential aaRS targets that may not have been initially predicted when analyzing aaRS functional sequences in isolation.

### Chemotherapeutic inhibition of multiple aminoacyl-tRNA synthetases may be relatively resistance-proof

Two of the fractions we described were effective inhibitors of both AlaRS and ThrRS in parasites. Although monotherapeutic inhibitors of aaRSs are highly effective [65], combination therapies involving multiple aaRSs have not been studied. Because aminoacylation pathways are integrated in parallel at the ribosome, the slowest aminoacylation pathway can be rate-limiting for protein synthesis and growth [24,66]. Thus, we expect the inhibition of multiple aaRSs to be antagonistic relative to Loewe Additivity expectations, in keeping with the Highest Single Agent (HSA) model [67,68]: single- or multiple-drug inhibition of multiple aaRSs should mask the potentially growth-restorative effects of resistance mutations arising in any one parasite aaRS gene. It is known that antagonistic combination chemotherapies are less prone to the evolution of resistance than single-drug or synergistic combination chemotherapies [32,69,70,71]. Therefore, chemotherapeutic inhibition of multiple aaRSs should be relatively less prone to the evolution of resistance than monotherapeutic or synergistic combination chemotherapeutic inhibition of single aaRSs. Further work is needed to test this hypothetical benefit.

### Conclusion

Trypanosome parasites pose a significant health risk worldwide. While current therapies exist, they are often also accompanied by off-target cytotoxicity and can lead to the rise of antimicrobial resistance. Here we have demonstrated that targeting tRNA-synthetase interactions have been an underexplored avenue for drug discovery. Using a combination of predictive computational tRNA network analyses and biochemical validation, we showed that aminoacyl-tRNA synthetases are a promising target for broad-spectrum anti-trypanosomal discovery with no significant consequence to the human counterpart target.

## Supporting information

S1 Fig Aragorn Score Distributions of Singleton vs. Co-Clustered Ara-Only Genes. (PDF)

S2 Fig Annotated and Missing Functional Classes in Union Annotation Gene Set. (PDF)

S3 Fig Bubbleplots of CIF divergence between humans and eight clades of trypanosomes for tRNA^Leu^. (PDF)

S4 Fig Bubbleplots of CIF divergence between humans and eight clades of trypanosomes for tRNA^Ile^. (PDF)

S5 Fig Bubbleplots of CIF divergence between humans and eight clades of trypanosomes for tRNA^Val^. (PDF)

S6 Fig Bubbleplots of CIF divergence between humans and eight clades of trypanosomes for tRNA^Arg^. (PDF)

S7 Fig Bubbleplots of CIF divergence between humans and eight clades of trypanosomes for tRNA^Cys^. (PDF)

S8 Fig Bubbleplots of CIF divergence between humans and eight clades of trypanosomes for tRNA^Met^. (PDF)

S9 Fig Bubbleplots of CIF divergence between humans and eight clades of trypanosomes for tRNA^Glu^. (PDF)

S10 Fig Bubbleplots of CIF divergence between humans and eight clades of trypanosomes for tRNA^Gln^. (PDF)

S11 Fig Bubbleplots of CIF divergence between humans and eight clades of trypanosomes for tRNA^Tyr^. (PDF)

S12 Fig Bubbleplots of CIF divergence between humans and eight clades of trypanosomes for tRNA^Trp^. (PDF)

S13 Fig Bubbleplots of CIF divergence between humans and eight clades of trypanosomes for tRNA^Ser^. (PDF)

S14 Fig Bubbleplots of CIF divergence between humans and eight clades of trypanosomes for tRNA^Thr^. (PDF)

S15 Fig Bubbleplots of CIF divergence between humans and eight clades of trypanosomes for tRNA^Pro^. (PDF)

S16 Fig Bubbleplots of CIF divergence between humans and eight clades of trypanosomes for tRNA^His^. (PDF)

S17 Fig Bubbleplots of CIF divergence between humans and eight clades of trypanosomes for tRNA^Gly^. (PDF)

S18 Fig Bubbleplots of CIF divergence between humans and eight clades of trypanosomes for tRNA^Asp^. (PDF)

S19 Fig Bubbleplots of CIF divergence between humans and eight clades of trypanosomes for tRNA^Asn^. (PDF)

S20 Fig Bubbleplots of CIF divergence between humans and eight clades of trypanosomes for tRNA^Lys^. (PDF)

S21 Fig Bubbleplots of CIF divergence between humans and eight clades of trypanosomes for tRNA^Phe^. (PDF)

S22 Fig Bubbleplots of CIF divergence between humans and eight clades of trypanosomes for tRNA^Ala^. (PDF)

S23 Fig Bubbleplots of CIF divergence between humans and eight clades of trypanosomes for tRNA^iMet^. (PDF)

S24 Fig Single-site function logos for Adenine in all clades, part I. (PNG)

S25 Fig Single-site function logos for Adenine in all clades, part II. (PNG)

S26 Fig Single-site function logos for Uracil in all clades, part I. (PNG)

S27 Fig Single-site function logos for Uracil in all clades, part II. (PNG)

S28 Fig Single-site function logos for Guanine in all clades, part I. (PNG)

S29 Fig Single-site function logos for Guanine in all clades, part II. (PNG)

S30 Fig Single-site function logos for Cytosine in all clades, part I. (PNG)

S31 Fig Single-site function logos for Cytosine in all clades, part II. (PNG)

S32 Fig Paired-site function logos for humans. (PNG)

S33 Fig Paired-site function logos for *L*. *major* clade. (PNG)

S34 Fig Paired-site function logos for *L*. *infantum* clade. (PNG)

S35 Fig Paired-site function logos for *L*. *mexicana* clade. (PNG)

S36 Fig Paired-site function logos for Viannia subclade. (PNG)

S37 Fig Paired-site function logos for *L*. *enriettii* clade. (PNG)

S38 Fig Paired-site function logos for *Leptomonas*/*Crithidia* clade. (PNG)

S39 Fig Paired-site function logos for American *Trypanosoma* clade. (PNG)

S40 Fig Paired-site function logos for African *Trypanosoma* clade. (PNG)

S41 Fig UPLC-qTOF base peak chromatogram of **(A)** RL12-182-HVF-D Sep-Pak Fraction C Subfraction 10–9 and **(B)** extracted ion chromatogram of peak 316.28 *m/z* in active region of trace.
(PDF)

S42 Fig UPLC-qTOF mass spectrum for peak at 316.28 *m/z*. (PDF)

S43 Fig Schematic diagram of the large-scale fermentation and extraction of RL12-182-HVF-D. (PDF)

S44 Fig HPLC-UV trace of the final preparative isolation step for the RL12-182-HVF-D active compound. Red box denotes active peak.
(PDF)

S45 Fig Marine natural product extract 2096D F10 inhibits *Leishmania major* AlaRS aminoacylation. (PDF)

S1 Table Similar and putatively homologous tRNA gene cluster variant groups of length at least three occurring in at least two *Leishmania* genomes. (PDF)

S2 Table Similar and putatively homologous tRNA gene cluster groups of length at least three occurring in at least two *Trypanosoma* genomes. (PDF)

S3 Table Similar and putatively homologous tRNA gene cluster groups of length at least three spanning both *Leishmania* (L) and *Trypanosoma* (T) genera genomes. Only groups 1, 3 and 4 contain five gene cluster variants conserved in both genera, namely, ASD, DSA, LXP (two variants), and QLI, where “X” represents the initiator iMet tRNA gene.
(PDF)

S4 Table Gene length, structure and functional type statistics on final annotation gene sets. (PDF)

S5 Table Nucleotide Composition of TriTryp tRNA Gene-Sets by Individual Genome Assembly, Organized by Clade (alternating background in the order of Table 1, boldface) or Excluded (at end of table in roman font). (PDF)

S6 Table Values of four replicate count-per-minute endpoints (taken at time t = 10 min. in time-course measurements as in Fig 9C) of *Leishmania major* AlaRS under DMSO or without added enzyme, their means and standard deviations, and their corresponding Z-factor in the scintillation counter-based aminoacylation time-course assay. (PDF)

S1 Code and Data Code and data to reproduce computational results. (TGZ)

S1 Text Supplementary methods for bacterial fermentation, natural product extraction, and active compound identification using HPLC-UV-MS and UPLC-ESI-qTOF-MS. (PDF)
