# Supplementary material for: Targeting tRNA-synthetase interactions towards novel therapeutic discovery against eukaryotic pathogens
Source: PLoS Negl Trop Dis. 2020 Feb 27;14(2):e0007983. doi: 10.1371/journal.pntd.0007983 (PMC7046186; doi:10.1371/journal.pntd.0007983)
Supplement: S1 Fig — (PDF) [file pntd.0007983.s001.pdf]

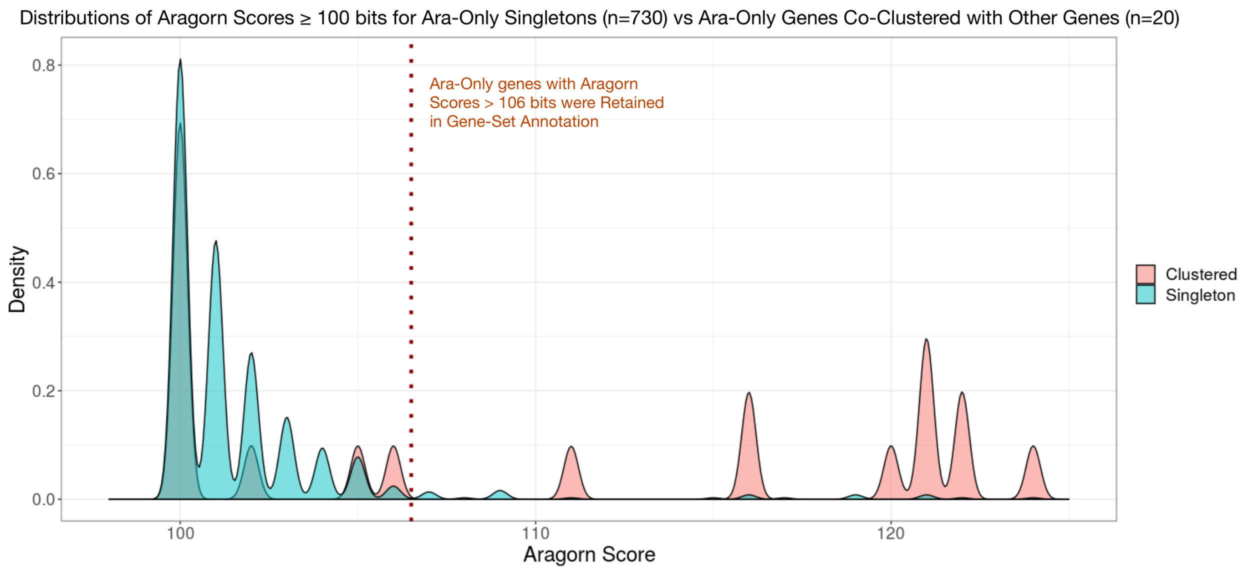
Distributions of Aragorn Scores ≥ 100 bits for Ara-Only Singletons (n=730) vs Ara-Only Genes Co-Clustered with Other Genes (n=20)
Ara-Only genes with Aragorn Scores > 106 bits were Retained in Gene-Set Annotation
Density
0.8
0.6
0.4
0.2
0.0
100
110
120
Aragorn Score
Clustered
Singleton
