# Supplementary material for: Targeting tRNA-synthetase interactions towards novel therapeutic discovery against eukaryotic pathogens
Source: PLoS Negl Trop Dis. 2020 Feb 27;14(2):e0007983. doi: 10.1371/journal.pntd.0007983 (PMC7046186; doi:10.1371/journal.pntd.0007983)

Number of tRNA Functional Classes and Missing Classes by Genome among 3571 Unambiguously Annotated Genes in Union Annotation Gene-Set

Missing classes labelled above bars. Ten additional intron-containing genes found by both gene-finders (two in *T. cruz* Tulacl2), were annotated as $tRNA^{Tyr}$ (in red).

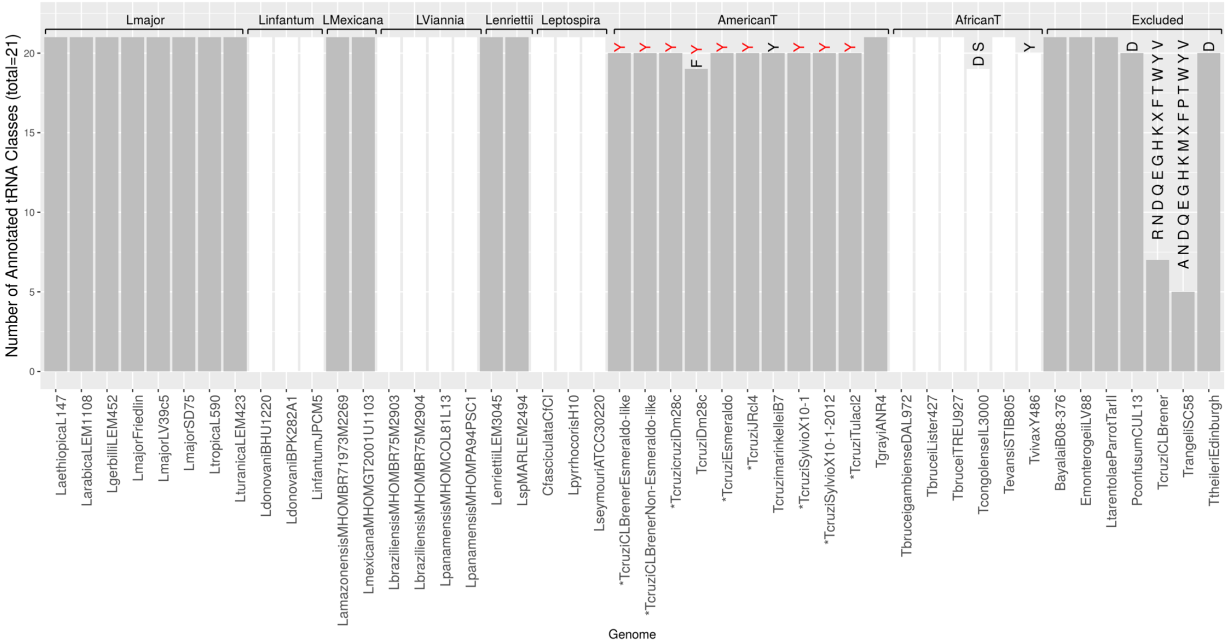

Supplement: S2 Fig — (PDF) [file pntd.0007983.s002.pdf]
