# Supplementary figures and images for: Targeting tRNA-synthetase interactions towards novel therapeutic discovery against eukaryotic pathogens
Source: PLoS Negl Trop Dis. 2020 Feb 27;14(2):e0007983. doi: 10.1371/journal.pntd.0007983 (PMC7046186; doi:10.1371/journal.pntd.0007983)

**Major (n = 8) L**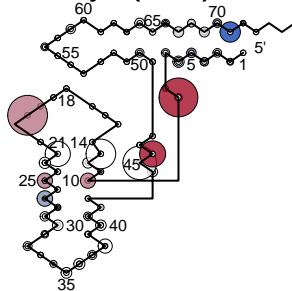**Infantum (n = 3) L**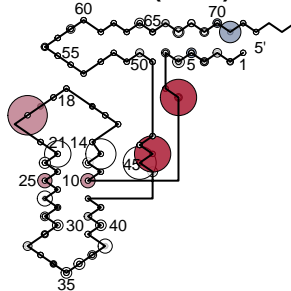**Mexicana (n = 2) L**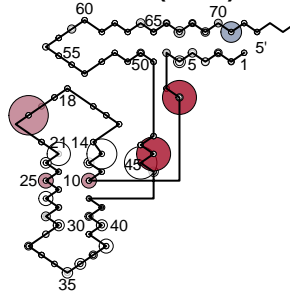**Viannia (n = 4) L**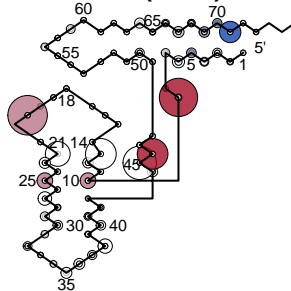**Af. Tryp. (n = 6) L**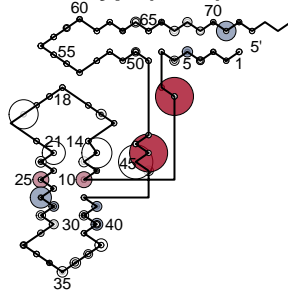**Am. Tryp. (n = 11) L**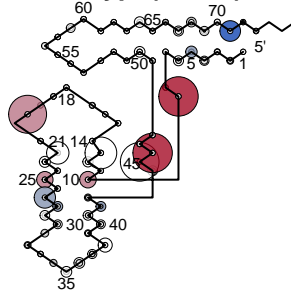**Lepto/Crith (n = 3) L**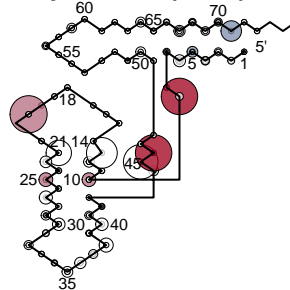**Enriettii (n = 2) L**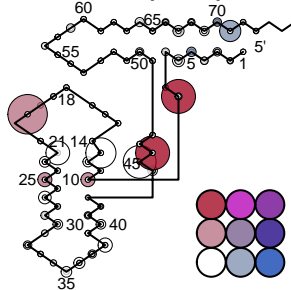

Supplement: S3 Fig — (PDF) [file pntd.0007983.s003.pdf]

**Major (n = 8) I**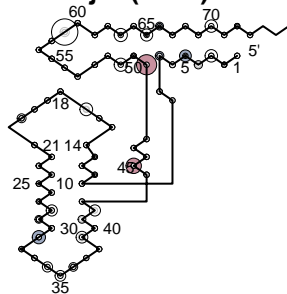**Infantum (n = 3) I**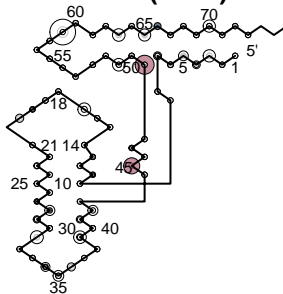**Mexicana (n = 2) I**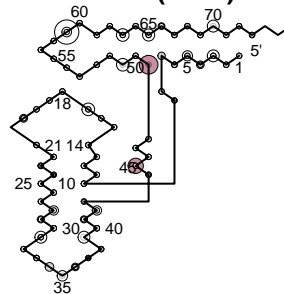**Viannia (n = 4) I**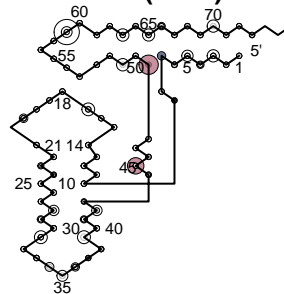**Af. Tryp. (n = 6) I**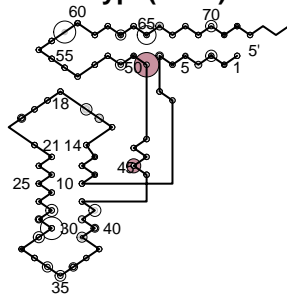**Am. Tryp. (n = 11) I**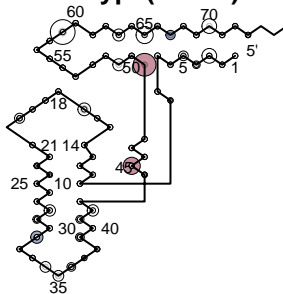**Lepto/Crith (n = 3) I**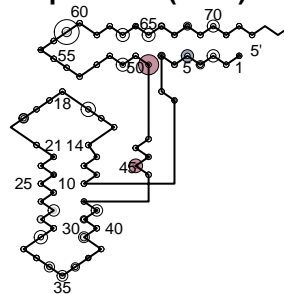**Enriettii (n = 2) I**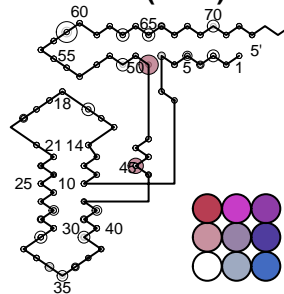

Supplement: S4 Fig — (PDF) [file pntd.0007983.s004.pdf]

**Major (n = 8) V**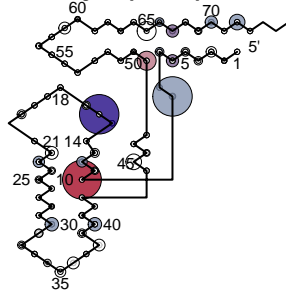**Infantum (n = 3) V**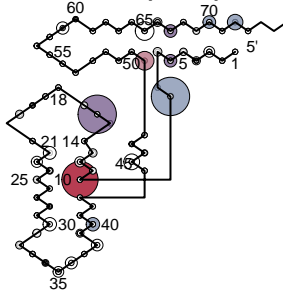**Mexicana (n = 2) V**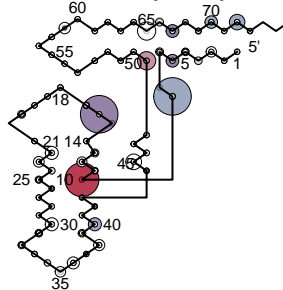**Viannia (n = 4) V**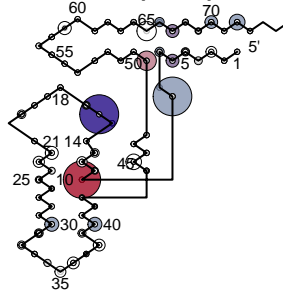**Af. Tryp. (n = 6) V**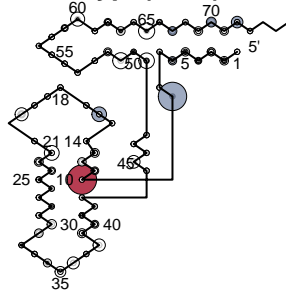**Am. Tryp. (n = 11) V**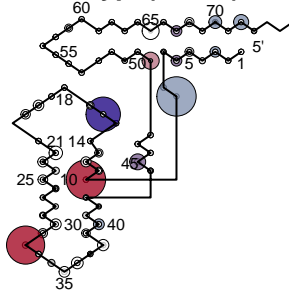**Lepto/Crith (n = 3) V**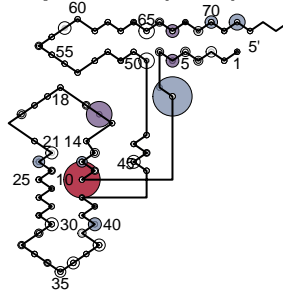**Enriettii (n = 2) V**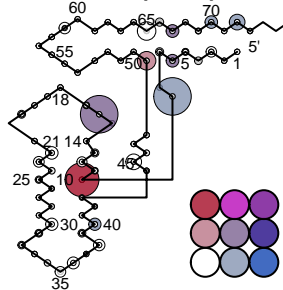

Supplement: S5 Fig — (PDF) [file pntd.0007983.s005.pdf]

**Major (n = 8) R**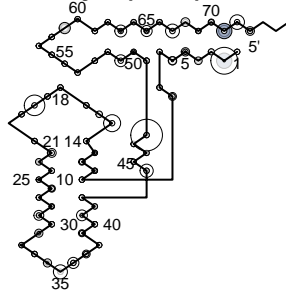**Infantum (n = 3) R**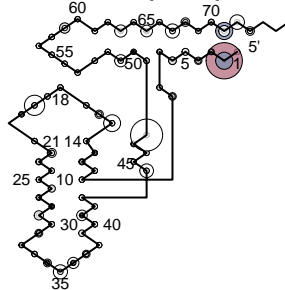**Mexicana (n = 2) R**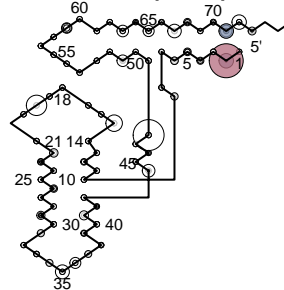**Viannia (n = 4) R**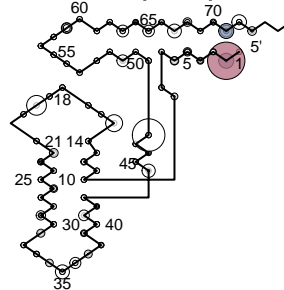**Af. Tryp. (n = 6) R**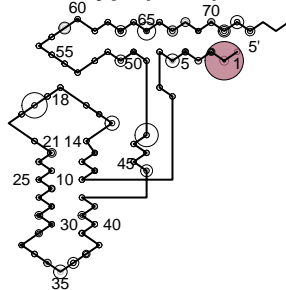**Am. Tryp. (n = 11) R**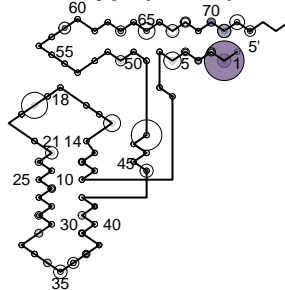**Lepto/Crith (n = 3) R**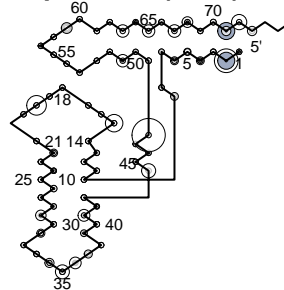**Enriettii (n = 2) R**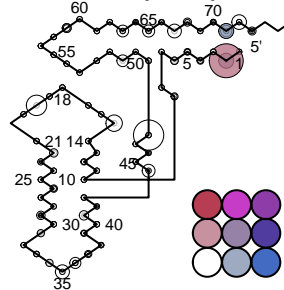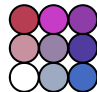

Supplement: S6 Fig — (PDF) [file pntd.0007983.s006.pdf]

**Major (n = 8) C**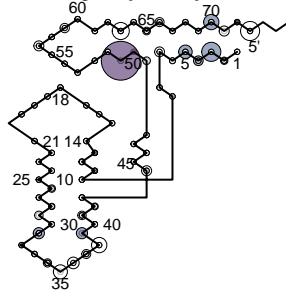**Infantum (n = 3) C**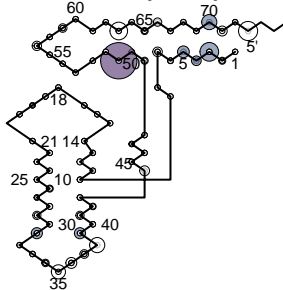**Mexicana (n = 2) C**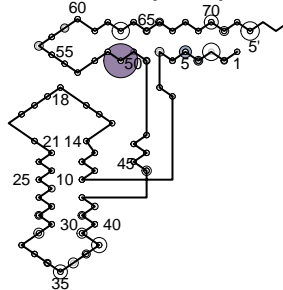**Viannia (n = 4) C**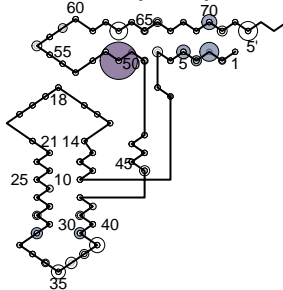**Af. Tryp. (n = 6) C**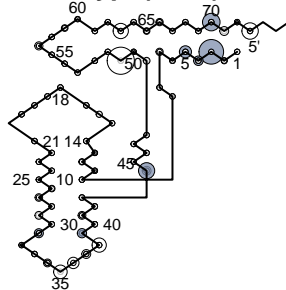**Am. Tryp. (n = 11) C**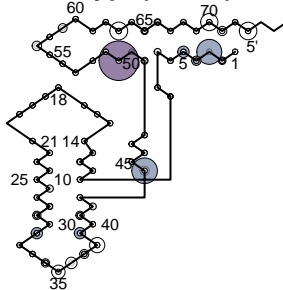**Lepto/Crith (n = 3) C**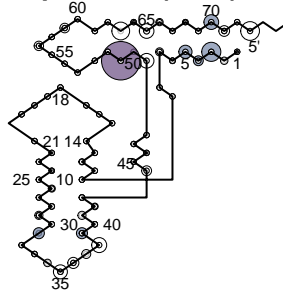**Enriettii (n = 2) C**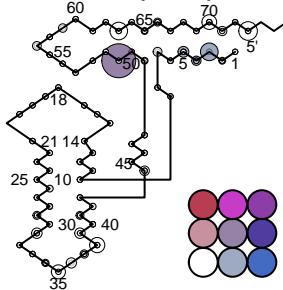

Supplement: S7 Fig — (PDF) [file pntd.0007983.s007.pdf]

**Major (n = 8) M**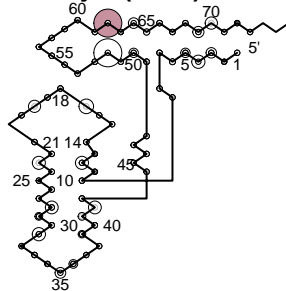**Infantum (n = 3) M**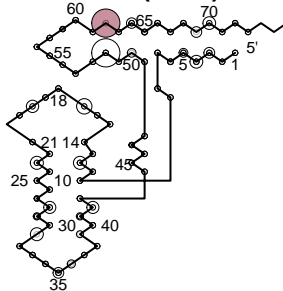**Mexicana (n = 2) M**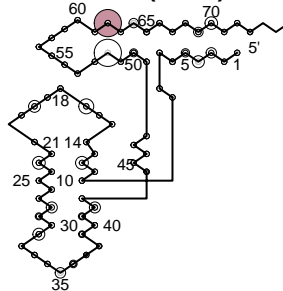**Viannia (n = 4) M**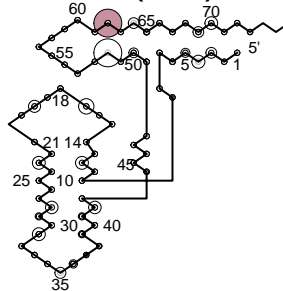**Af. Tryp. (n = 6) M**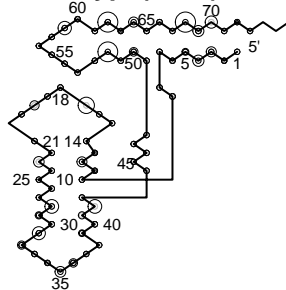**Am. Tryp. (n = 11) M**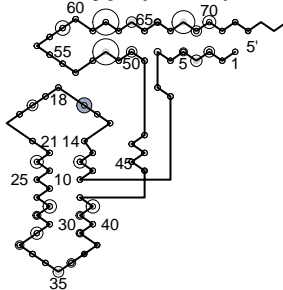**Lepto/Crith (n = 3) M**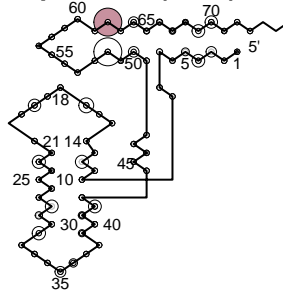**Enriettii (n = 2) M**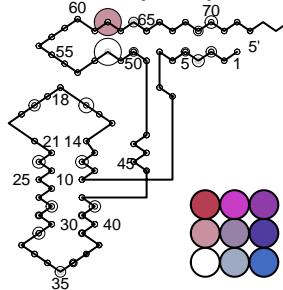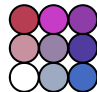

Supplement: S8 Fig — (PDF) [file pntd.0007983.s008.pdf]

**Major (n = 8) E**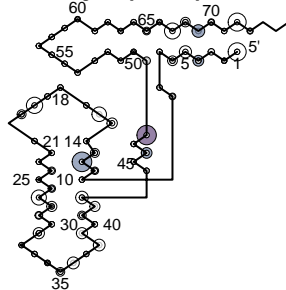**Infantum (n = 3) E**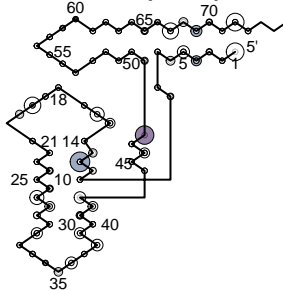**Mexicana (n = 2) E**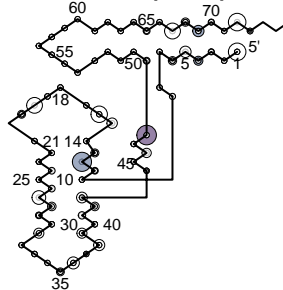**Viannia (n = 4) E**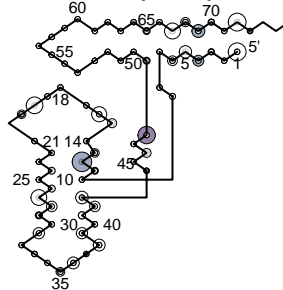**Af. Tryp. (n = 6) E**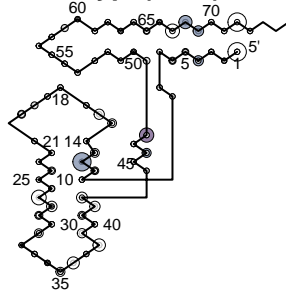**Am. Tryp. (n = 11) E**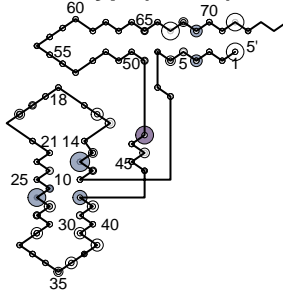**Lepto/Crith (n = 3) E**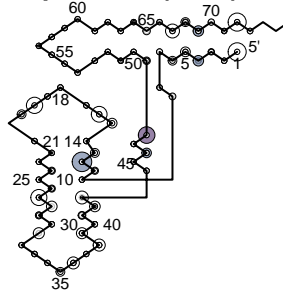**Enriettii (n = 2) E**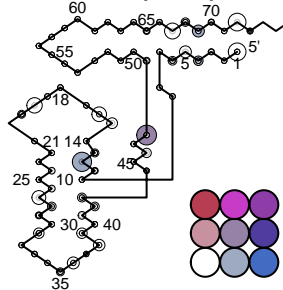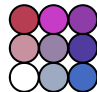

Supplement: S9 Fig — (PDF) [file pntd.0007983.s009.pdf]

**Major (n = 8) Q**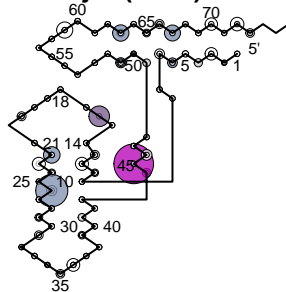**Infantum (n = 3) Q**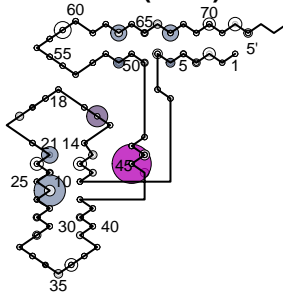**Mexicana (n = 2) Q**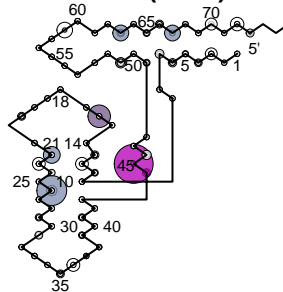**Viannia (n = 4) Q**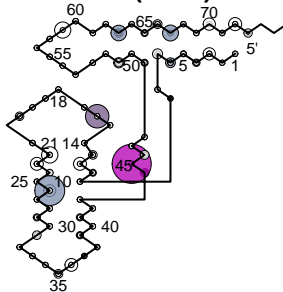**Af. Tryp. (n = 6) Q**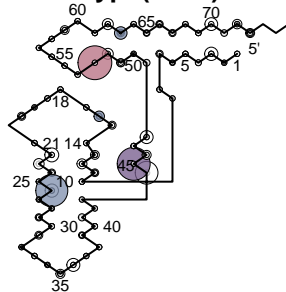**Am. Tryp. (n = 11) Q**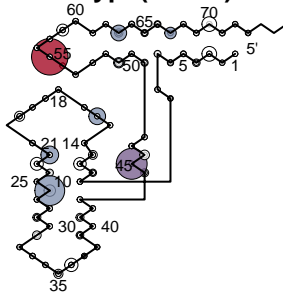**Lepto/Crith (n = 3) Q**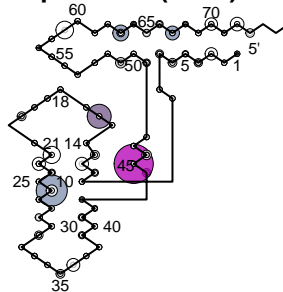**Enriettii (n = 2) Q**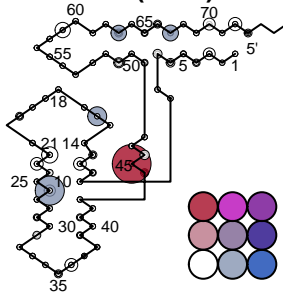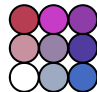

Supplement: S10 Fig — (PDF) [file pntd.0007983.s010.pdf]

**Major (n = 8) Y**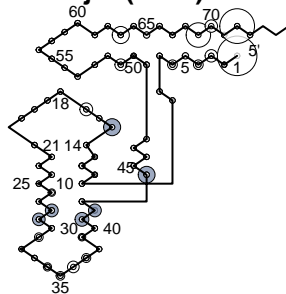**Infantum (n = 3) Y**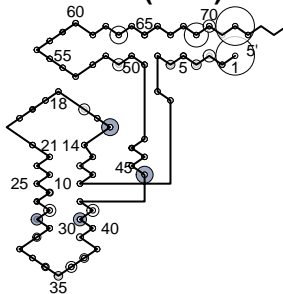**Mexicana (n = 2) Y**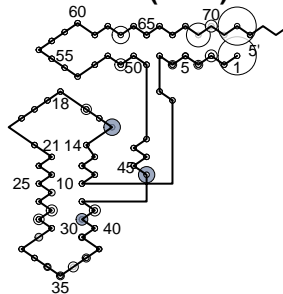**Viannia (n = 4) Y**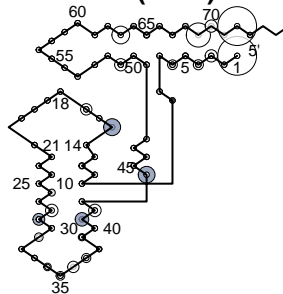**Af. Tryp. (n = 6) Y**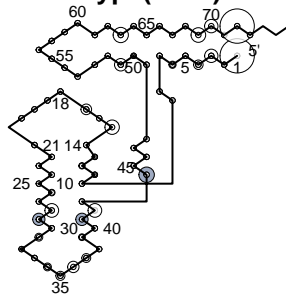**Am. Tryp. (n = 11) Y**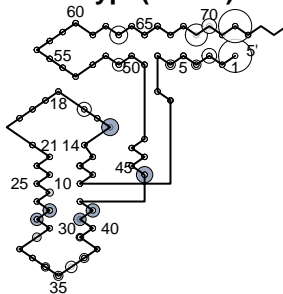**Lepto/Crith (n = 3) Y**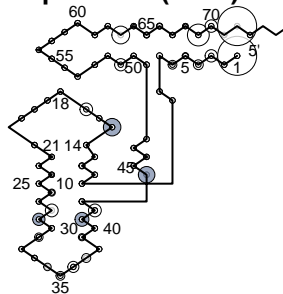**Enriettii (n = 2) Y**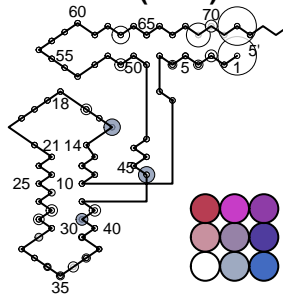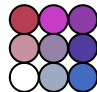

Supplement: S11 Fig — (PDF) [file pntd.0007983.s011.pdf]

**Major (n = 8) W**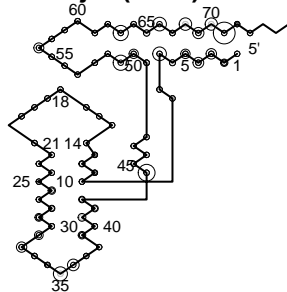**Infantum (n = 3) W**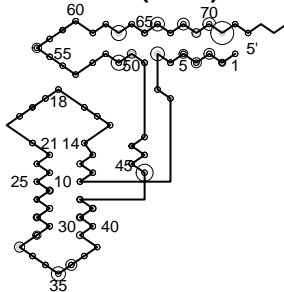**Mexicana (n = 2) W**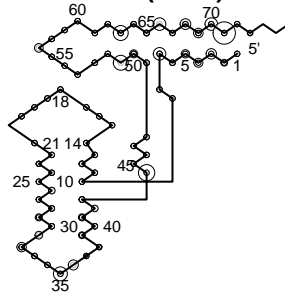**Viannia (n = 4) W**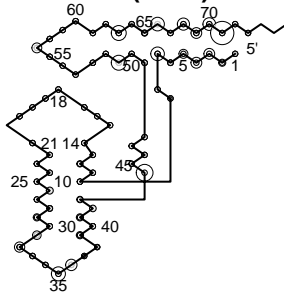**Af. Tryp. (n = 6) W**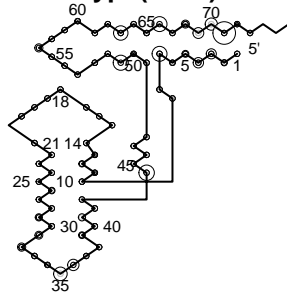**Am. Tryp. (n = 11) W**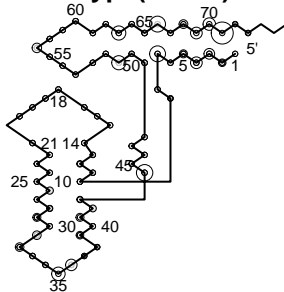**Lepto/Crith (n = 3) W**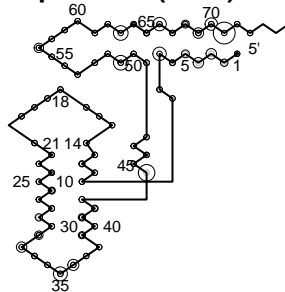**Enriettii (n = 2) W**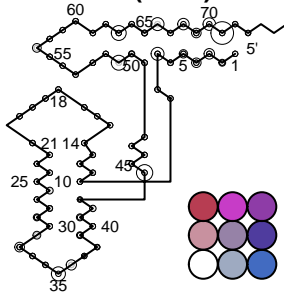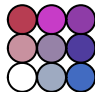

Supplement: S12 Fig — (PDF) [file pntd.0007983.s012.pdf]

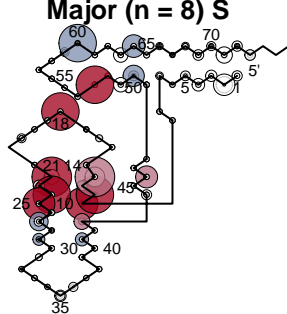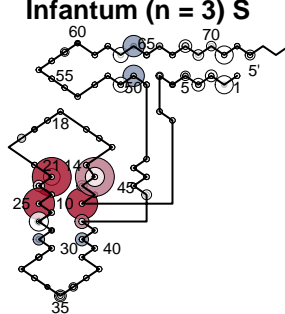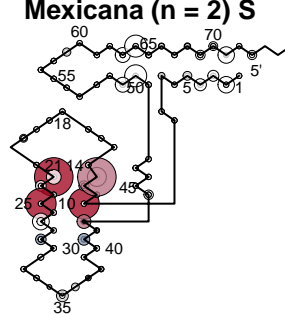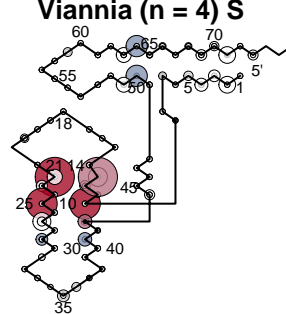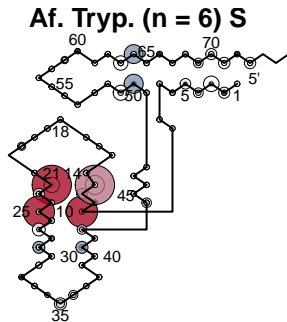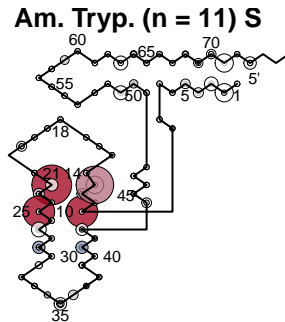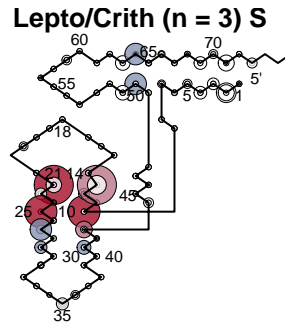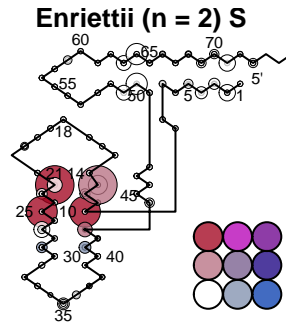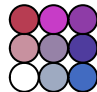

Supplement: S13 Fig — (PDF) [file pntd.0007983.s013.pdf]

**Major (n = 8) T**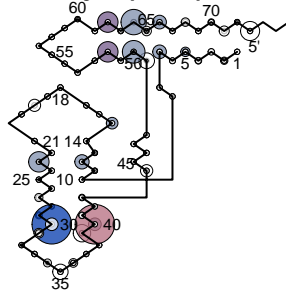**Infantum (n = 3) T**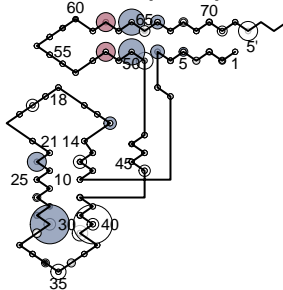**Mexicana (n = 2) T**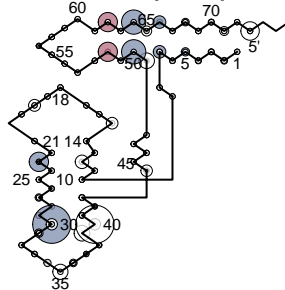**Viannia (n = 4) T**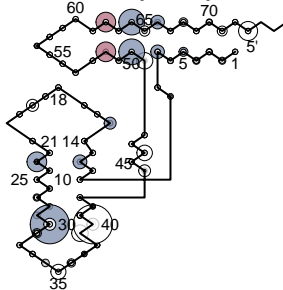**Af. Tryp. (n = 6) T**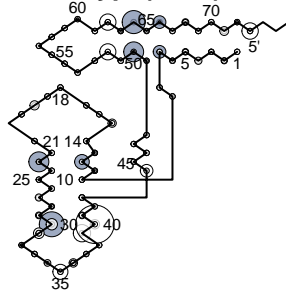**Am. Tryp. (n = 11) T**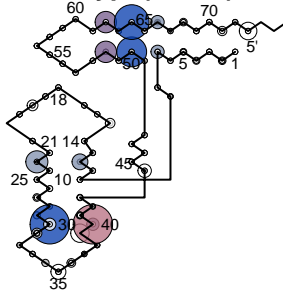**Lepto/Crith (n = 3) T**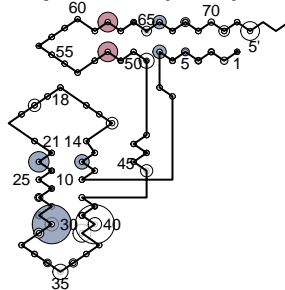**Enriettii (n = 2) T**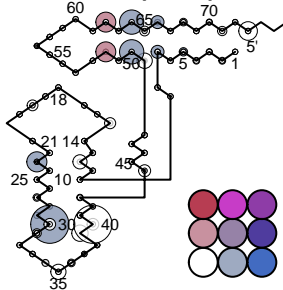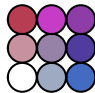

Supplement: S14 Fig — (PDF) [file pntd.0007983.s014.pdf]

**Major (n = 8) P**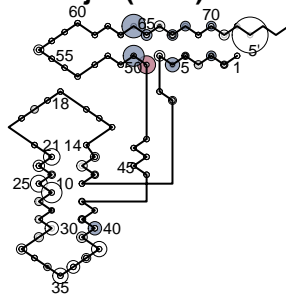**Infantum (n = 3) P**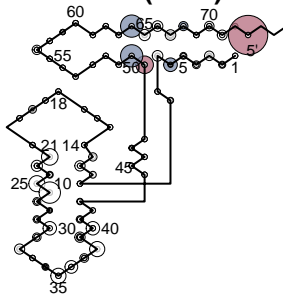**Mexicana (n = 2) P**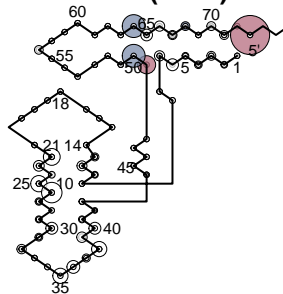**Viannia (n = 4) P**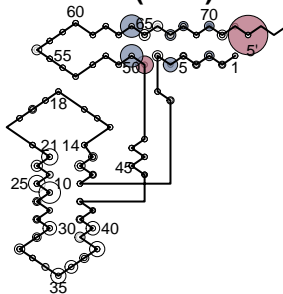**Af. Tryp. (n = 6) P**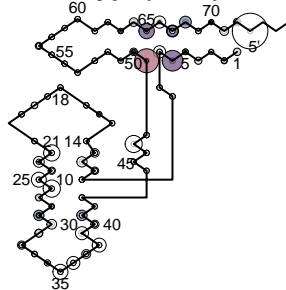**Am. Tryp. (n = 11) P**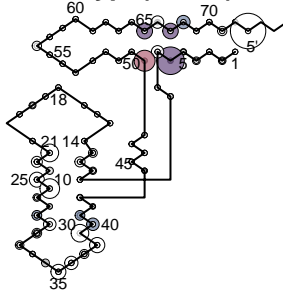**Lepto/Crith (n = 3) P**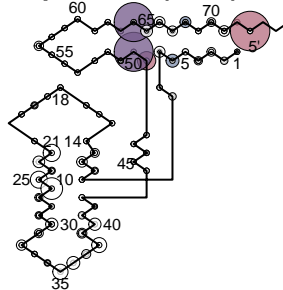**Enriettii (n = 2) P**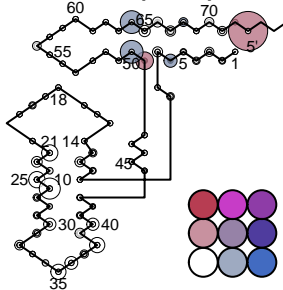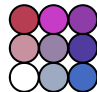

Supplement: S15 Fig — (PDF) [file pntd.0007983.s015.pdf]

**Major (n = 8) H**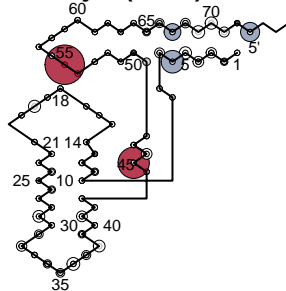**Infantum (n = 3) H**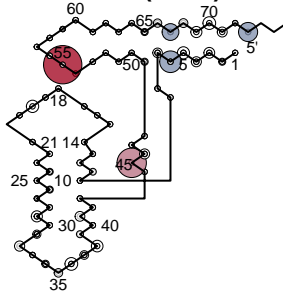**Mexicana (n = 2) H**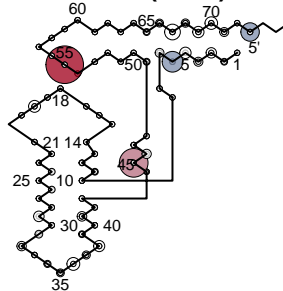**Viannia (n = 4) H**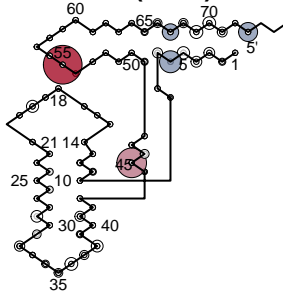**Af. Tryp. (n = 6) H**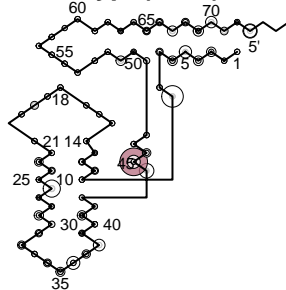**Am. Tryp. (n = 11) H**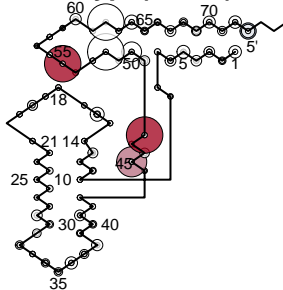**Lepto/Crith (n = 3) H**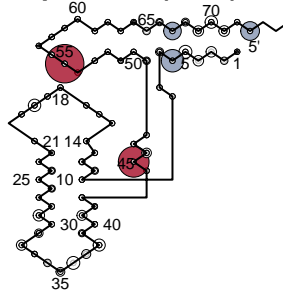**Enriettii (n = 2) H**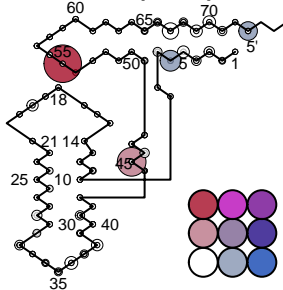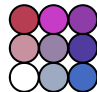

Supplement: S16 Fig — (PDF) [file pntd.0007983.s016.pdf]

**Major (n = 8) G**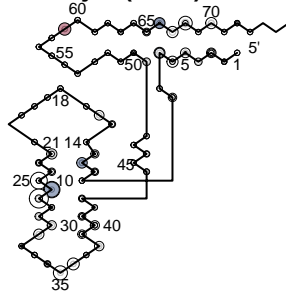**Infantum (n = 3) G**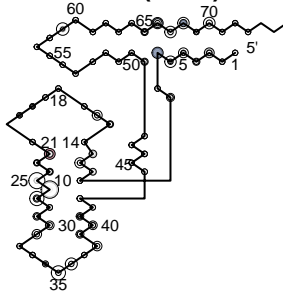**Mexicana (n = 2) G**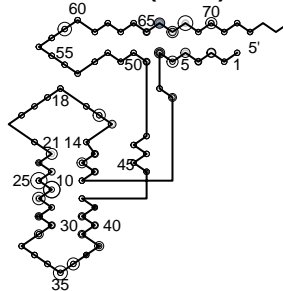**Viannia (n = 4) G**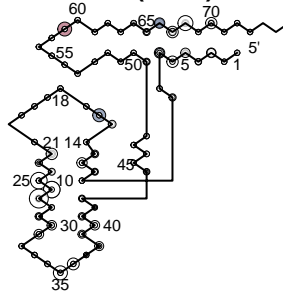**Af. Tryp. (n = 6) G**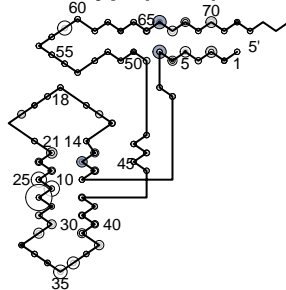**Am. Tryp. (n = 11) G**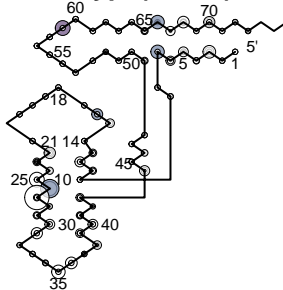**Lepto/Crith (n = 3) G**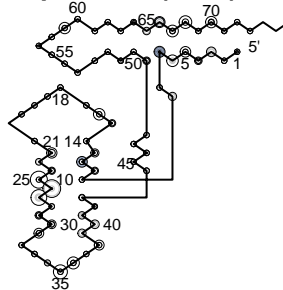**Enriettii (n = 2) G**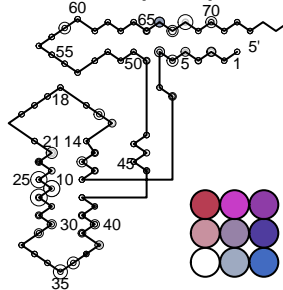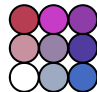

Supplement: S17 Fig — (PDF) [file pntd.0007983.s017.pdf]

**Major (n = 8) D**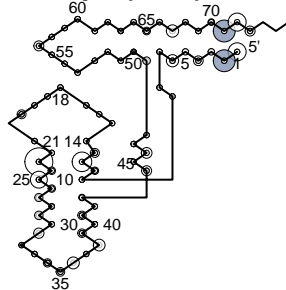**Infantum (n = 3) D**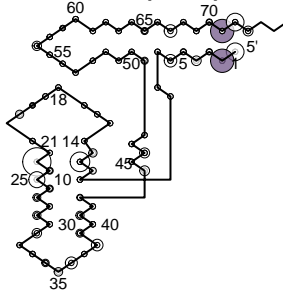**Mexicana (n = 2) D**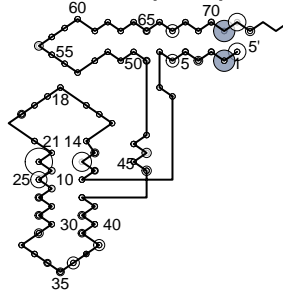**Viannia (n = 4) D**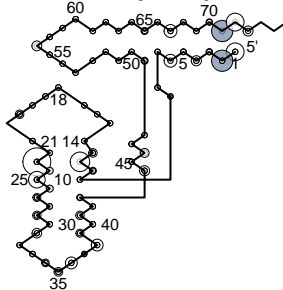**Af. Tryp. (n = 6) D**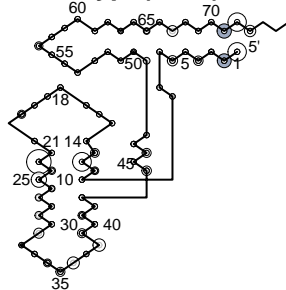**Am. Tryp. (n = 11) D**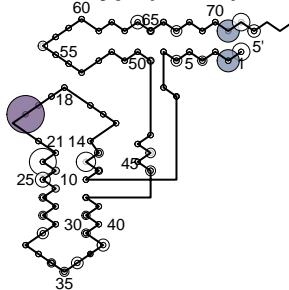**Lepto/Crith (n = 3) D**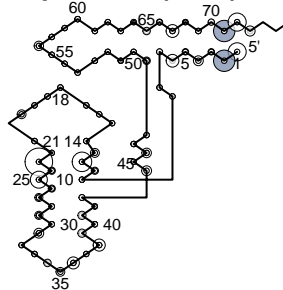**Enriettii (n = 2) D**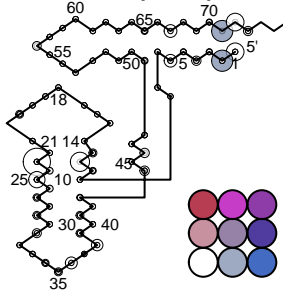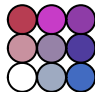

Supplement: S18 Fig — (PDF) [file pntd.0007983.s018.pdf]

**Major (n = 8) N**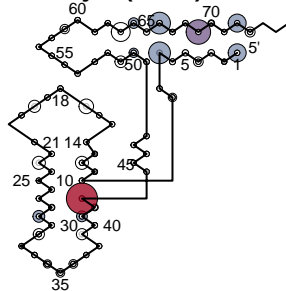**Infantum (n = 3) N**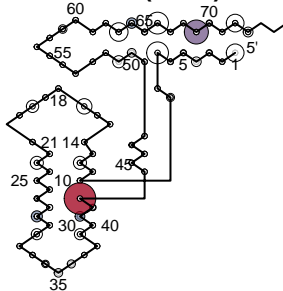**Mexicana (n = 2) N**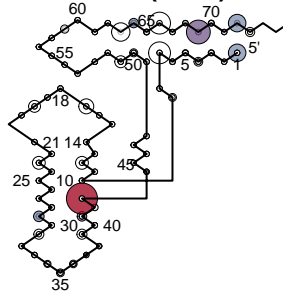**Viannia (n = 4) N**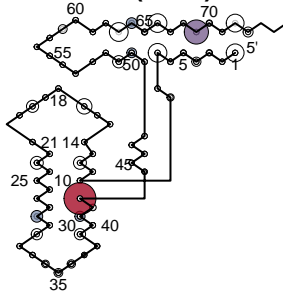**Af. Tryp. (n = 6) N**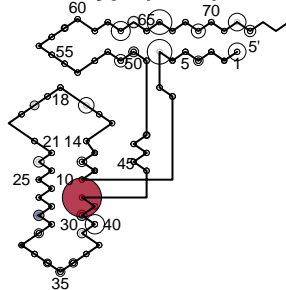**Am. Tryp. (n = 11) N**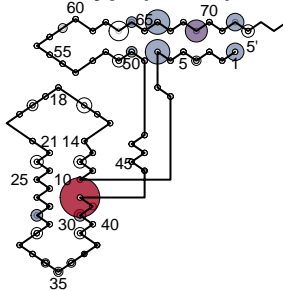**Lepto/Crith (n = 3) N**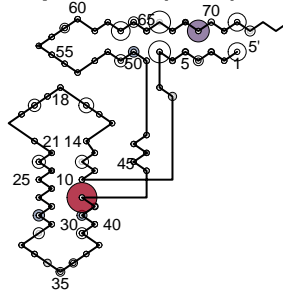**Enriettii (n = 2) N**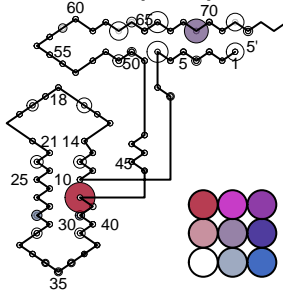

Supplement: S19 Fig — (PDF) [file pntd.0007983.s019.pdf]

**Major (n = 8) K**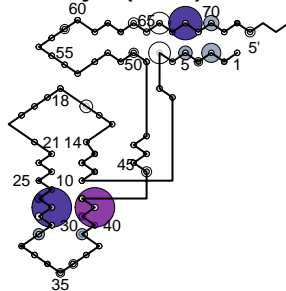**Infantum (n = 3) K**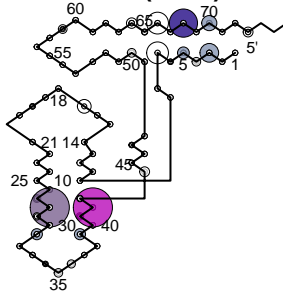**Mexicana (n = 2) K**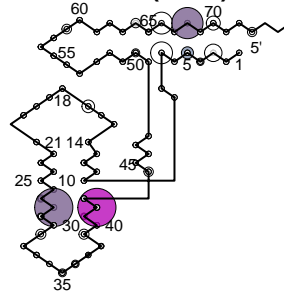**Viannia (n = 4) K**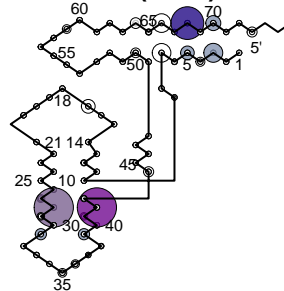**Af. Tryp. (n = 6) K**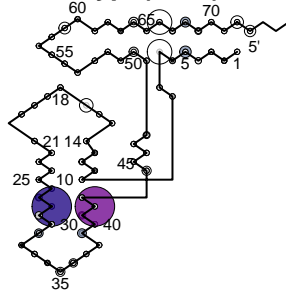**Am. Tryp. (n = 11) K**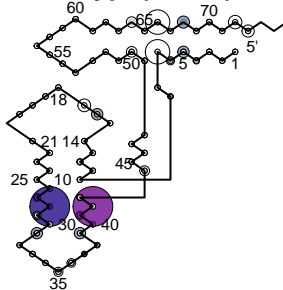**Lepto/Crith (n = 3) K**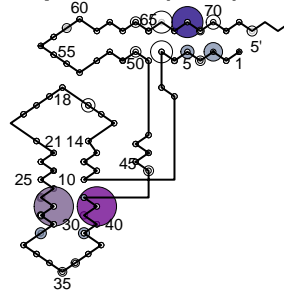**Enriettii (n = 2) K**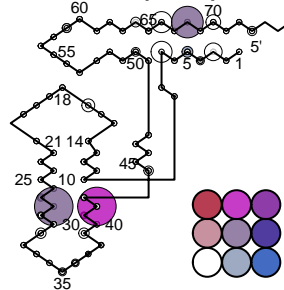

Supplement: S20 Fig — (PDF) [file pntd.0007983.s020.pdf]

**Major (n = 8) F**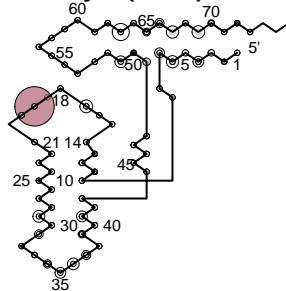**Infantum (n = 3) F**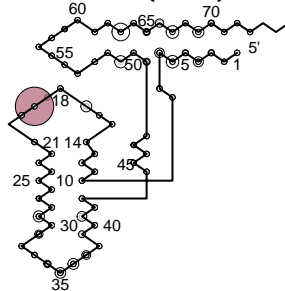**Mexicana (n = 2) F**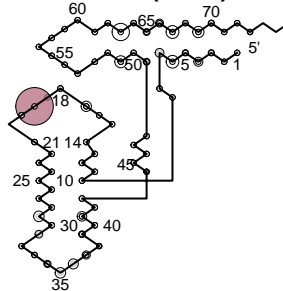**Viannia (n = 4) F**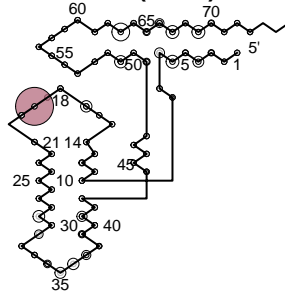**Af. Tryp. (n = 6) F**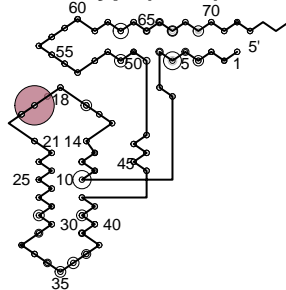**Am. Tryp. (n = 11) F**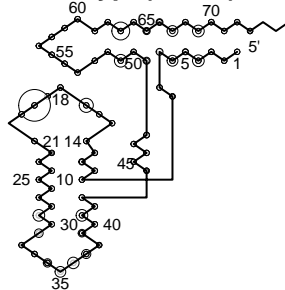**Lepto/Crith (n = 3) F**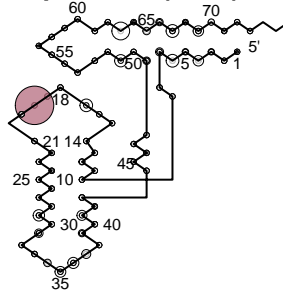**Enriettii (n = 2) F**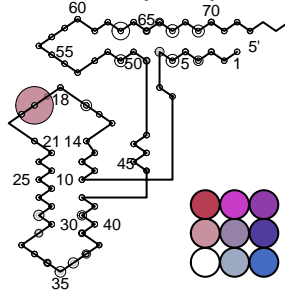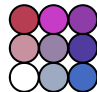

Supplement: S21 Fig — (PDF) [file pntd.0007983.s021.pdf]

**Major (n = 8) A**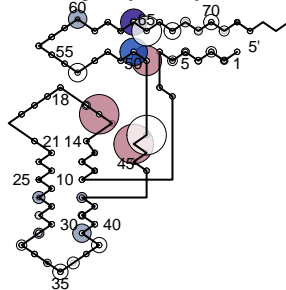**Infantum (n = 3) A**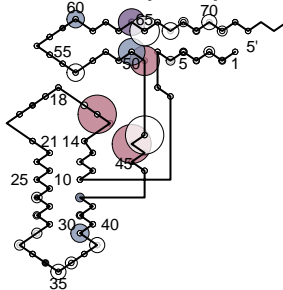**Mexicana (n = 2) A**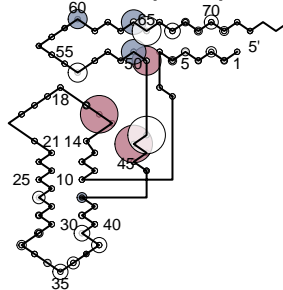**Viannia (n = 4) A**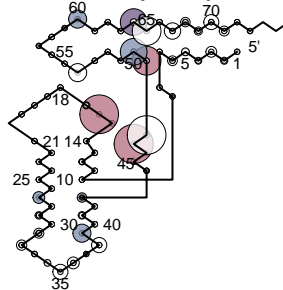**Af. Tryp. (n = 6) A**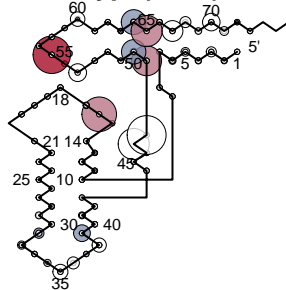**Am. Tryp. (n = 11) A**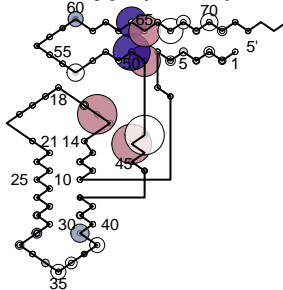**Lepto/Crith (n = 3) A**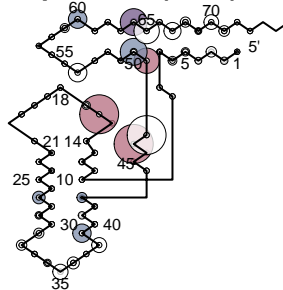**Enriettii (n = 2) A**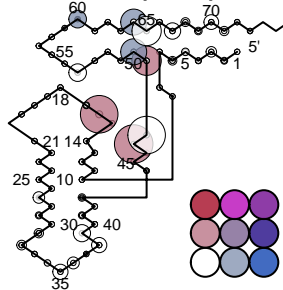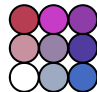

Supplement: S22 Fig — (PDF) [file pntd.0007983.s022.pdf]

**Major (n = 8) X**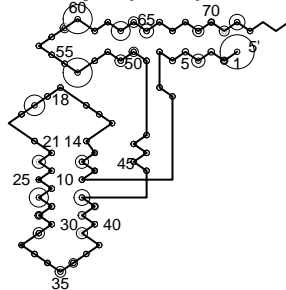**Infantum (n = 3) X**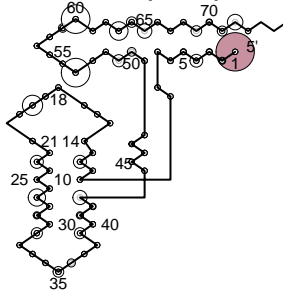**Mexicana (n = 2) X**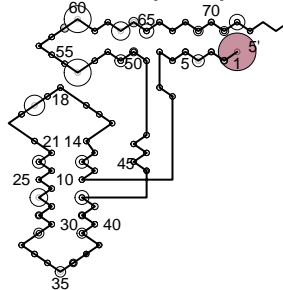**Viannia (n = 4) X**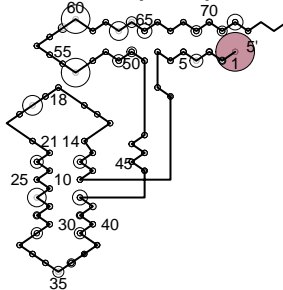**Af. Tryp. (n = 6) X**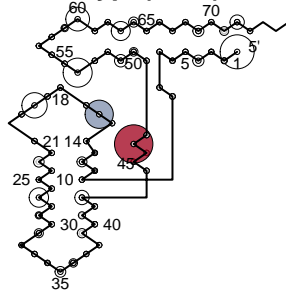**Am. Tryp. (n = 11) X**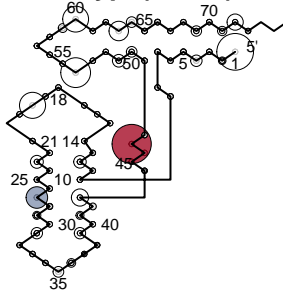**Lepto/Crith (n = 3) X**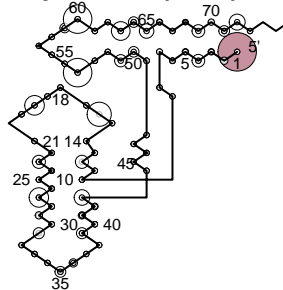**Enriettii (n = 2) X**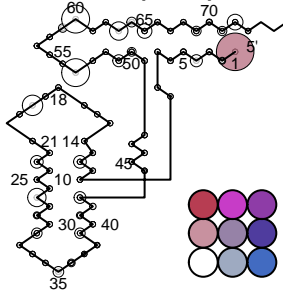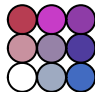

Supplement: S23 Fig — (PDF) [file pntd.0007983.s023.pdf]

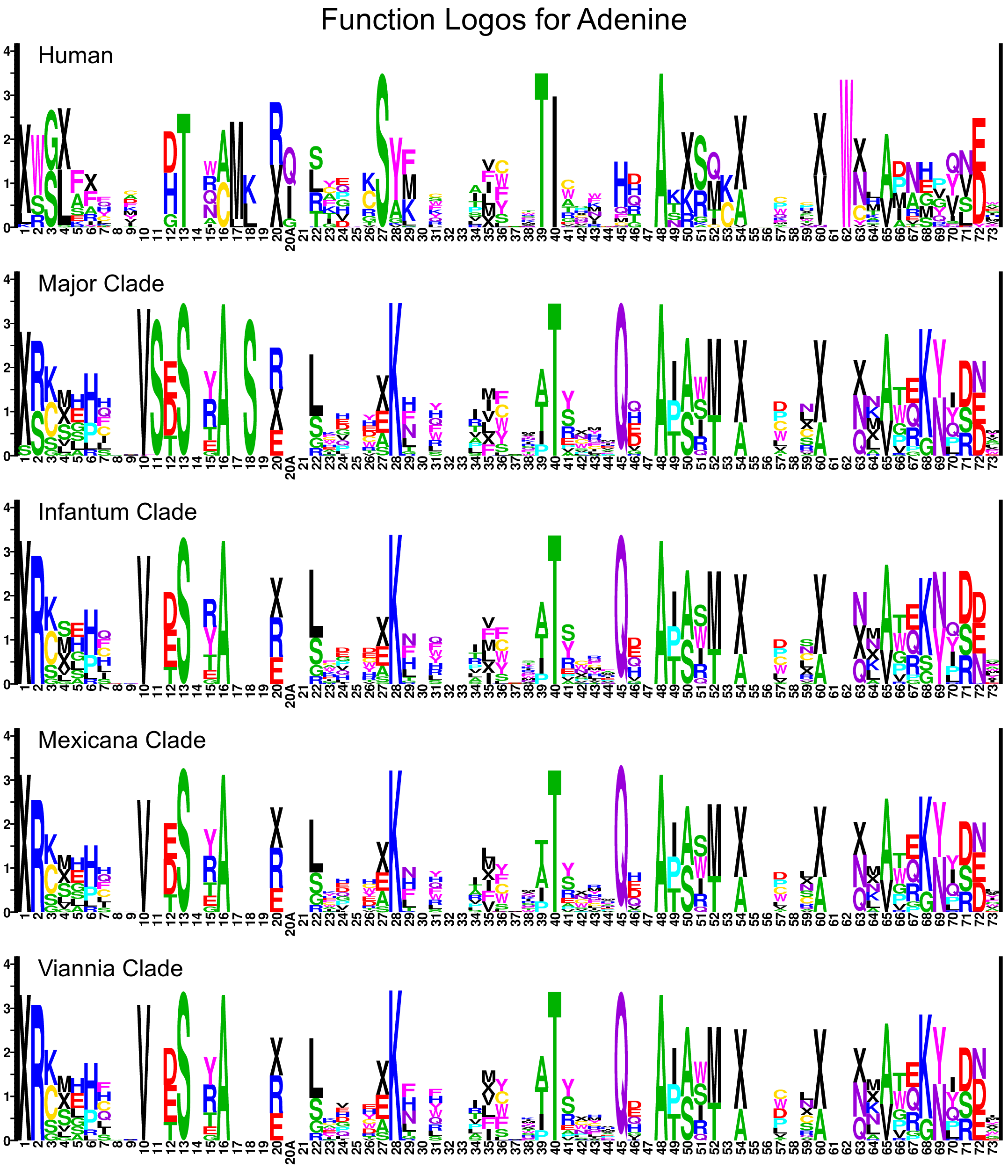

Supplement: S24 Fig — (PNG) [file pntd.0007983.s024.png]

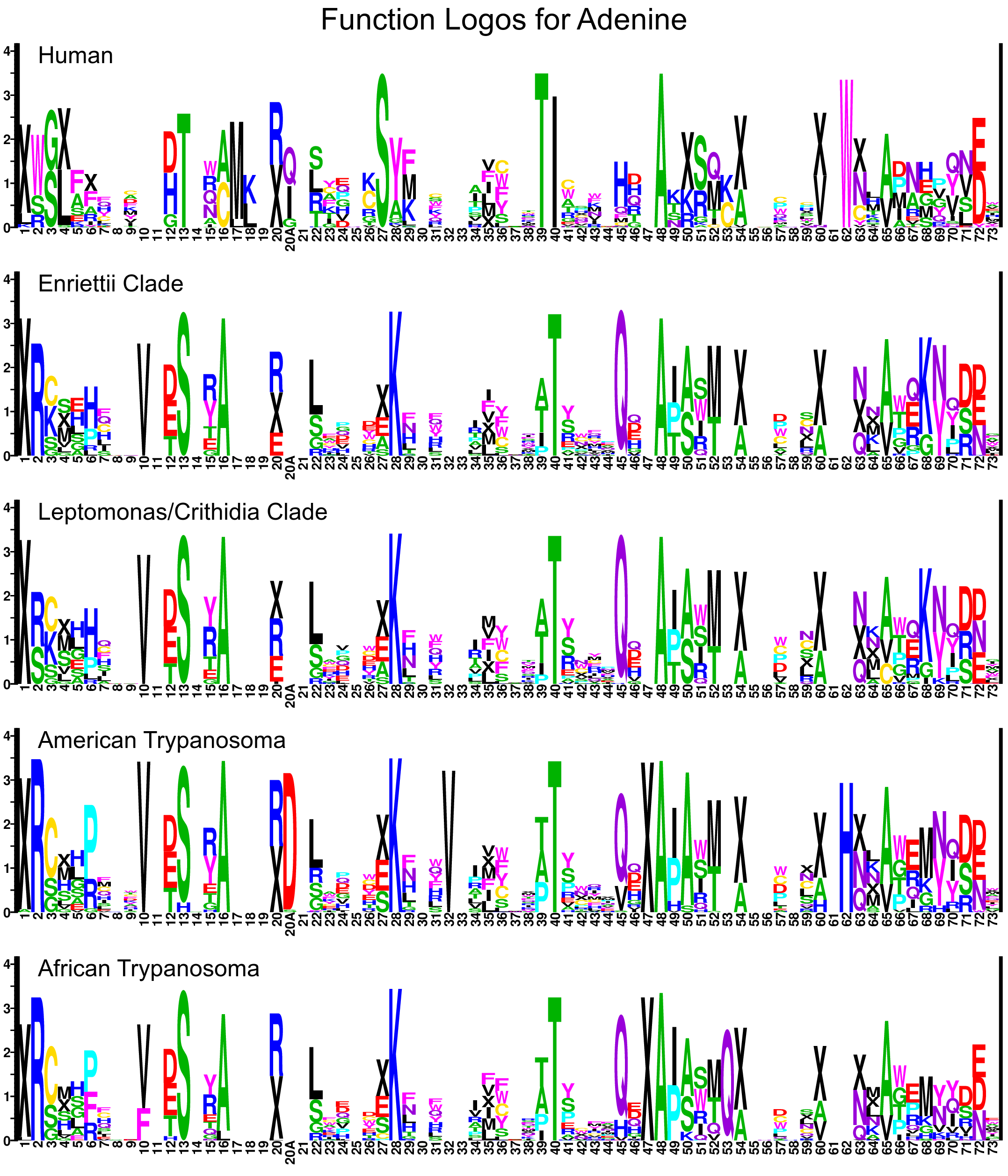

Supplement: S25 Fig — (PNG) [file pntd.0007983.s025.png]

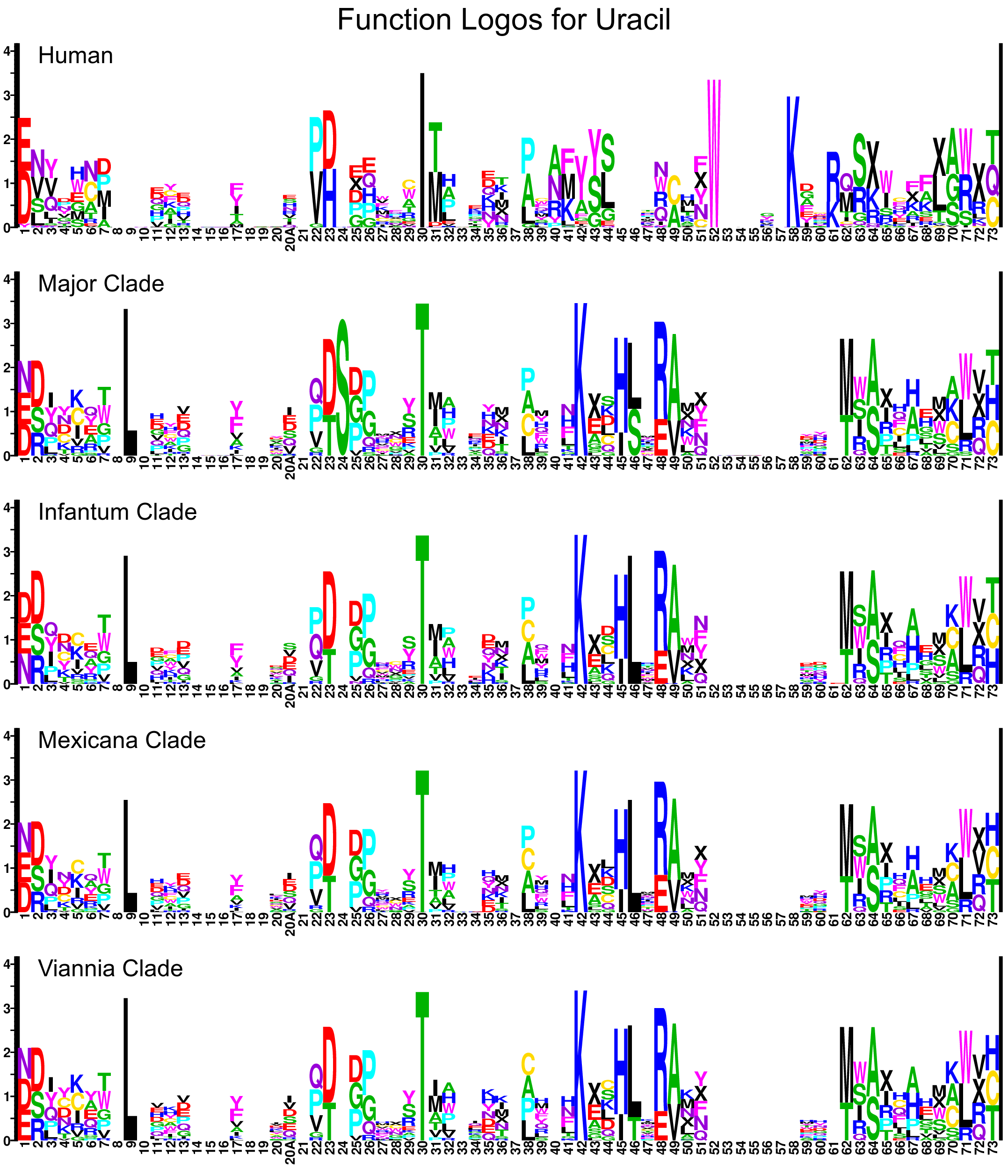

Supplement: S26 Fig — (PNG) [file pntd.0007983.s026.png]

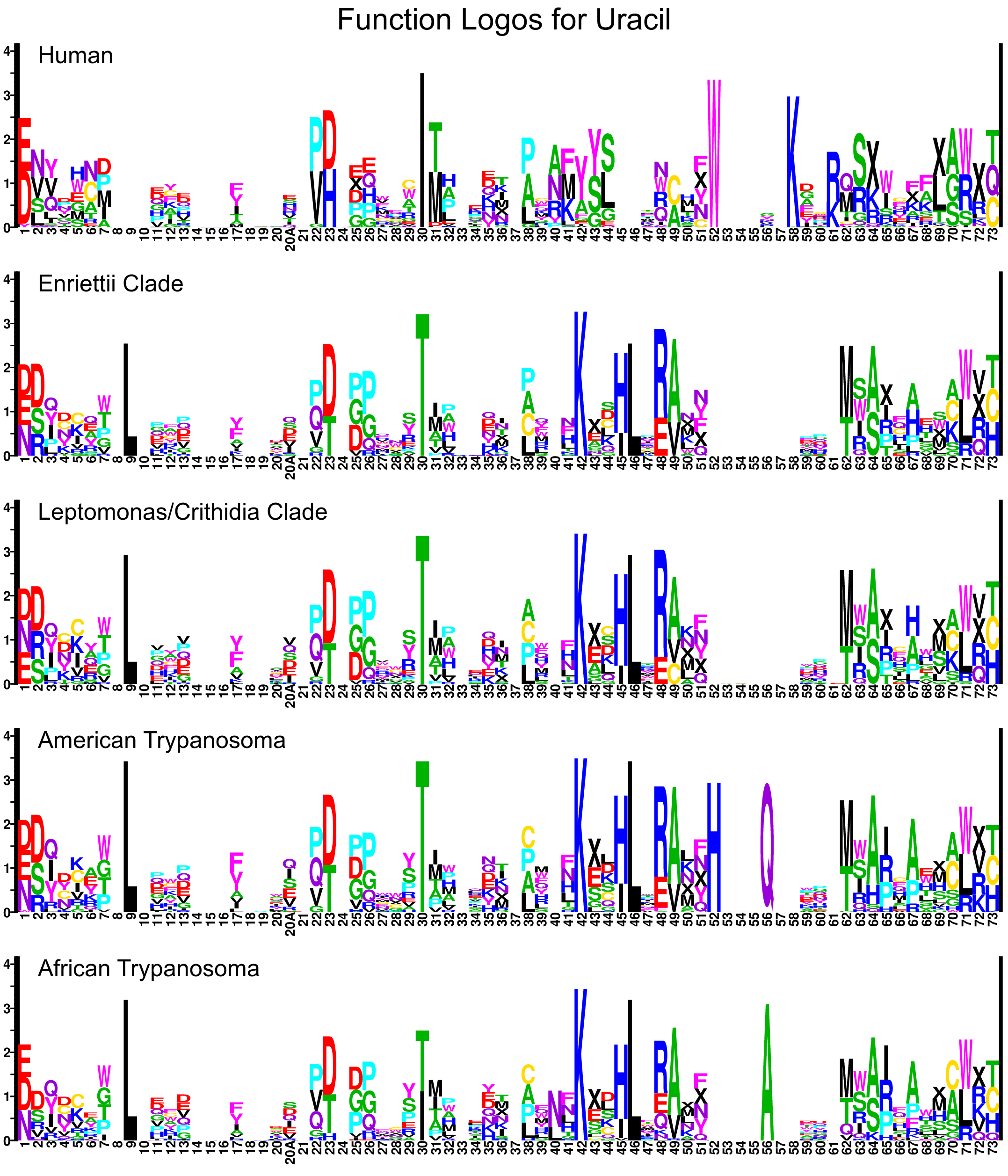

Supplement: S27 Fig — (PNG) [file pntd.0007983.s027.png]

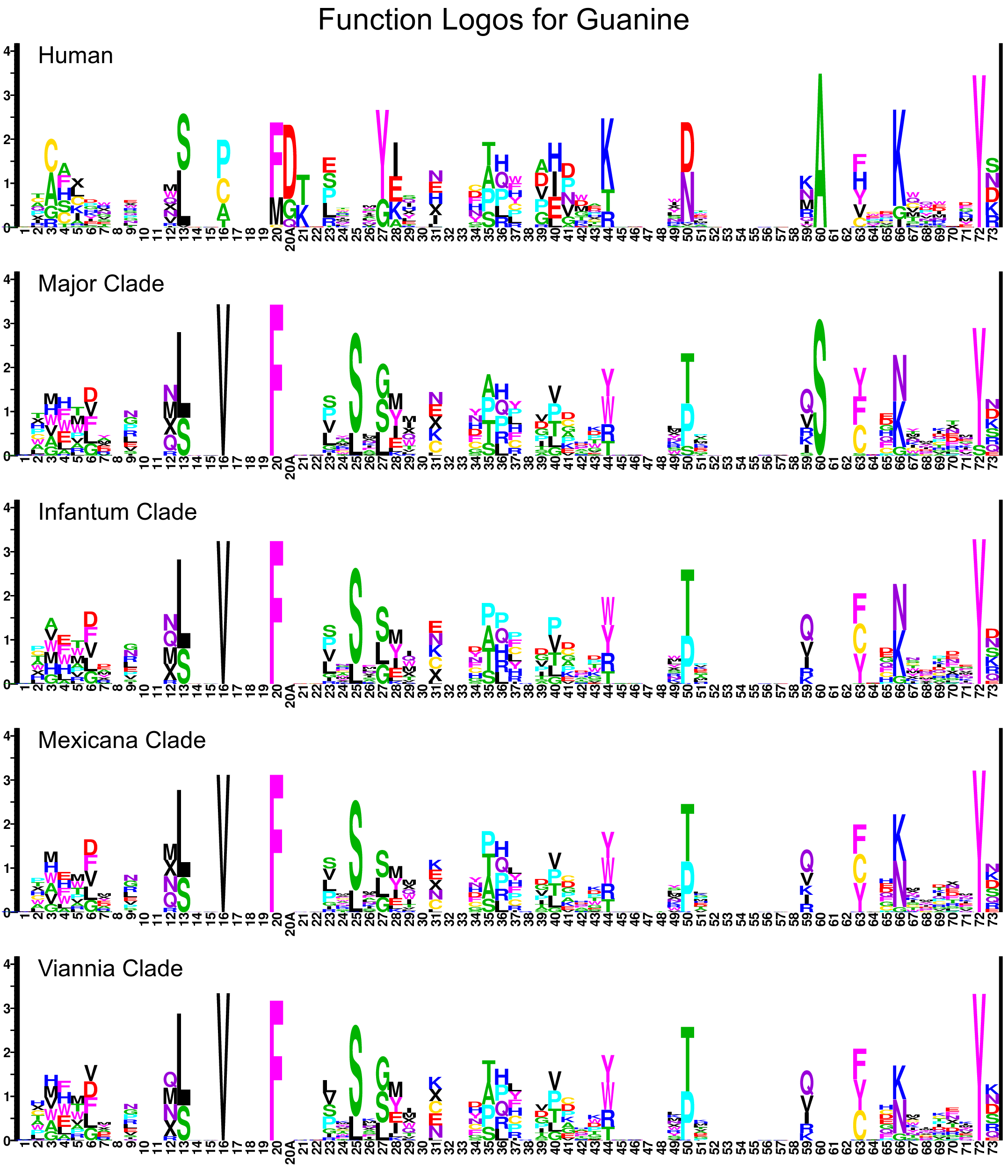

Supplement: S28 Fig — (PNG) [file pntd.0007983.s028.png]

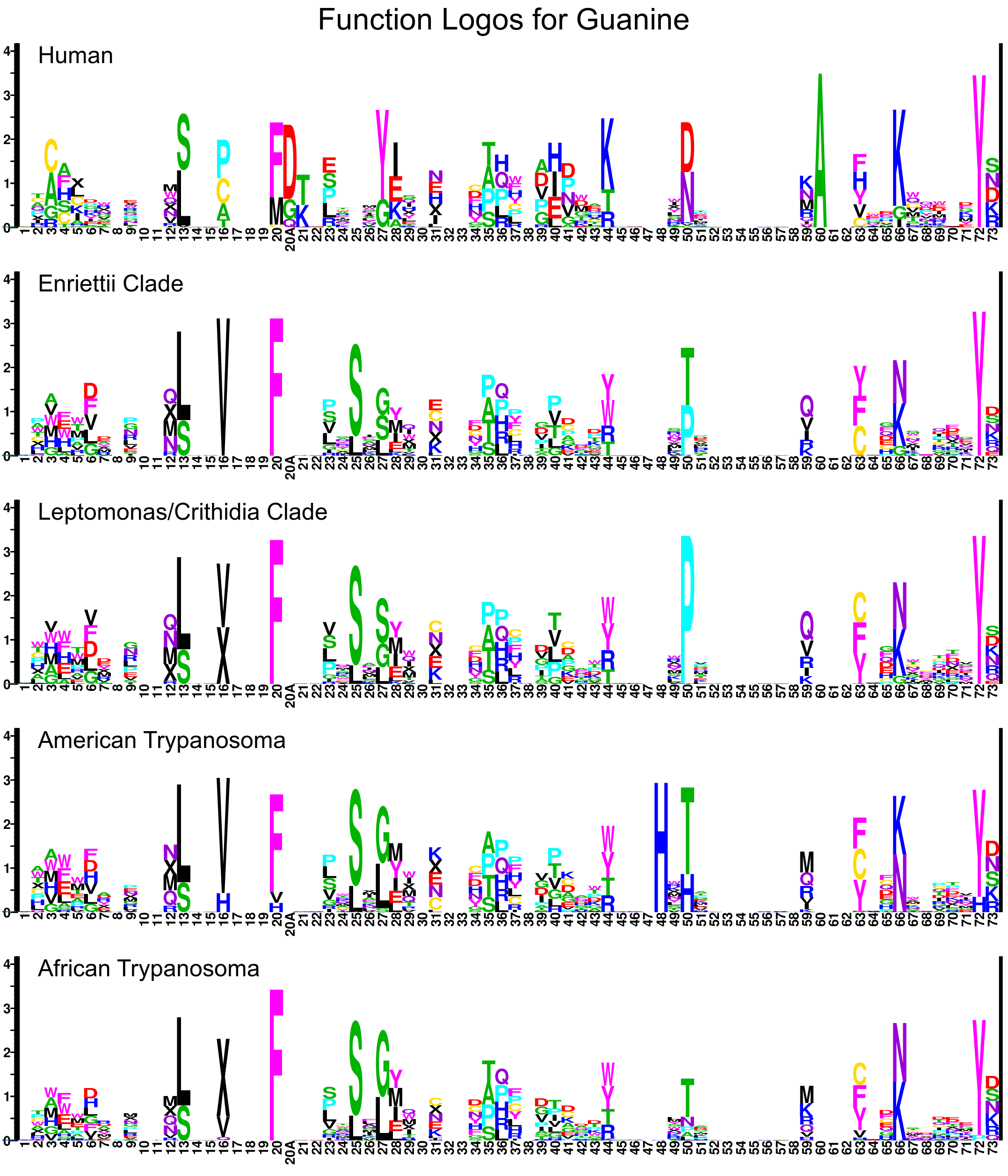

Supplement: S29 Fig — (PNG) [file pntd.0007983.s029.png]

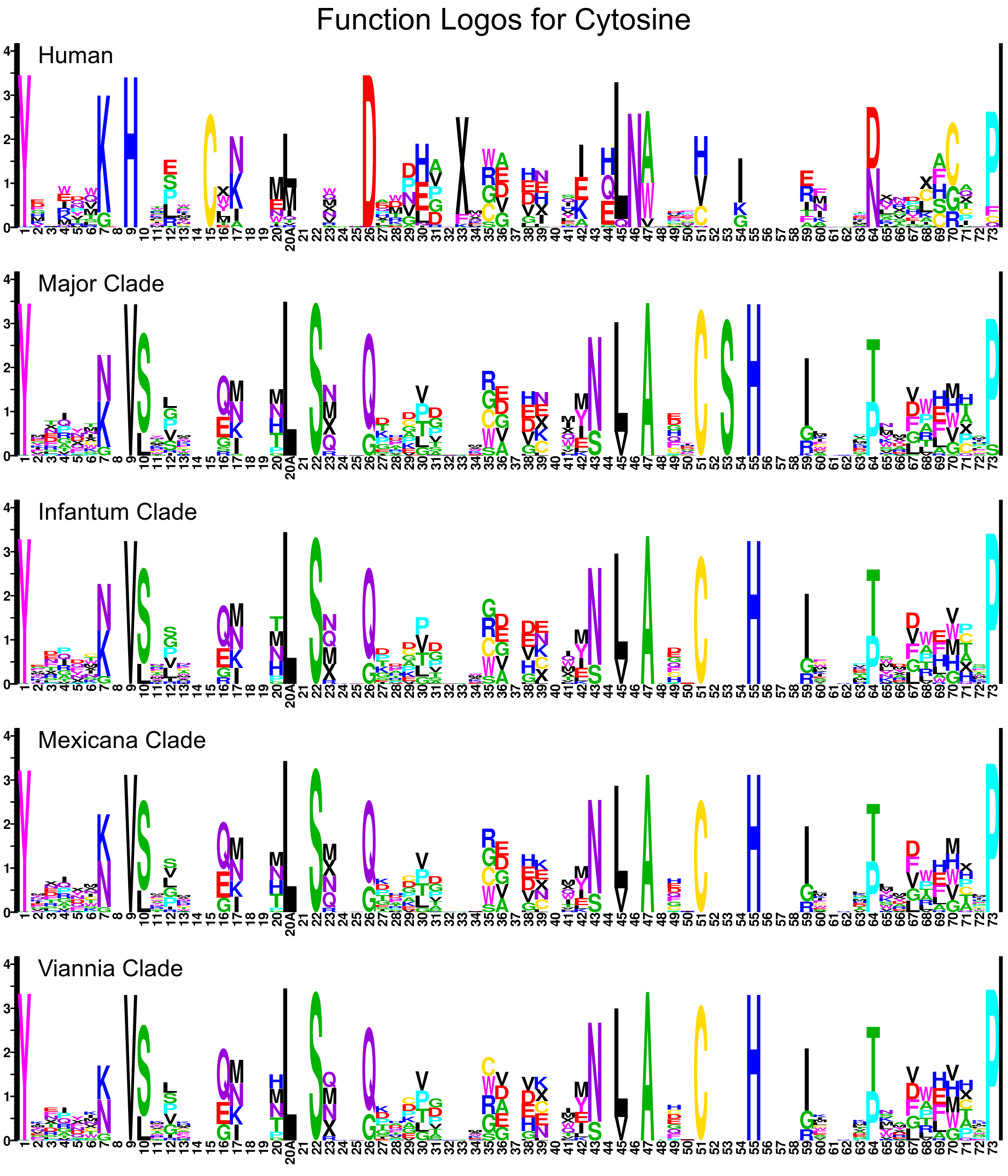

Supplement: S30 Fig — (PNG) [file pntd.0007983.s030.png]

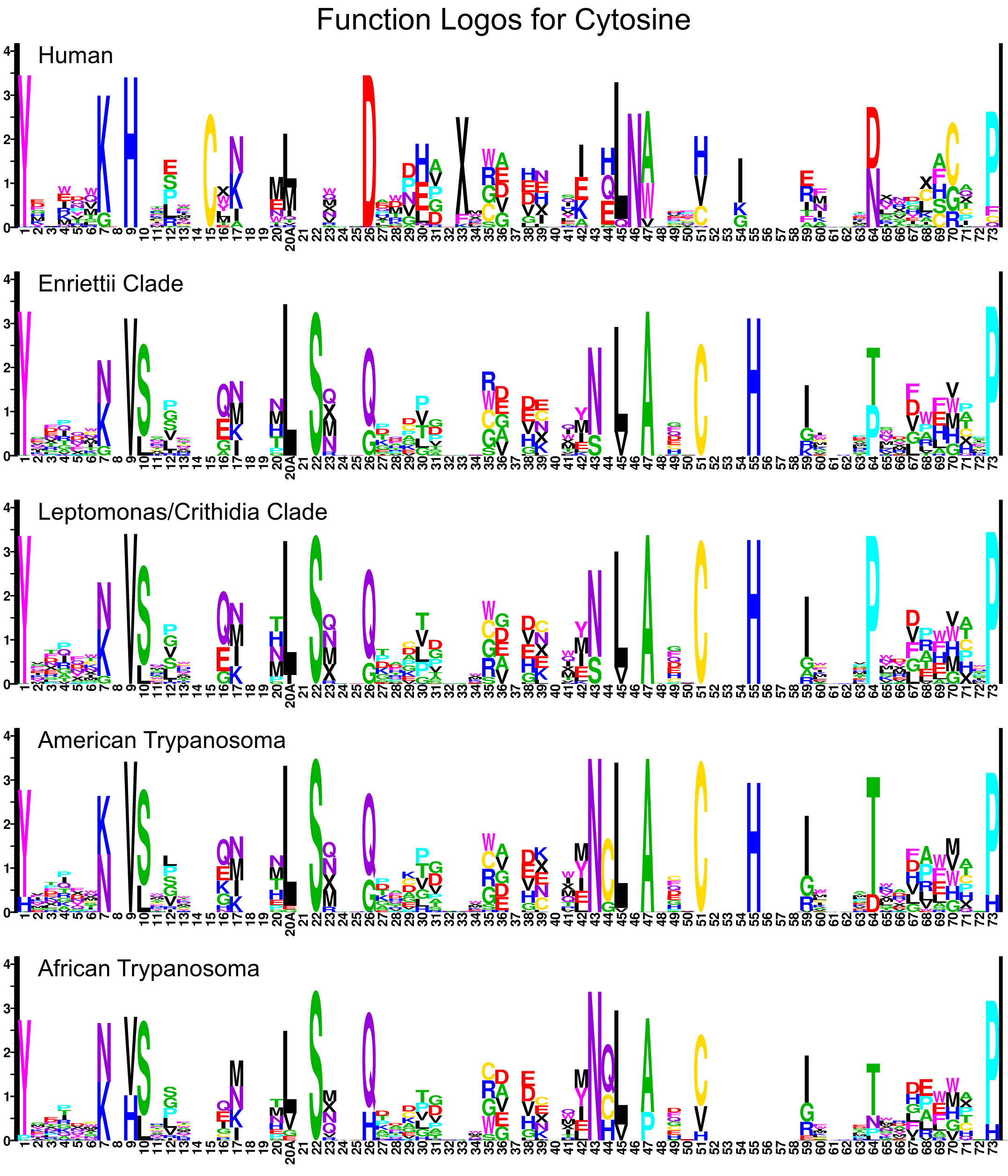

Supplement: S31 Fig — (PNG) [file pntd.0007983.s031.png]

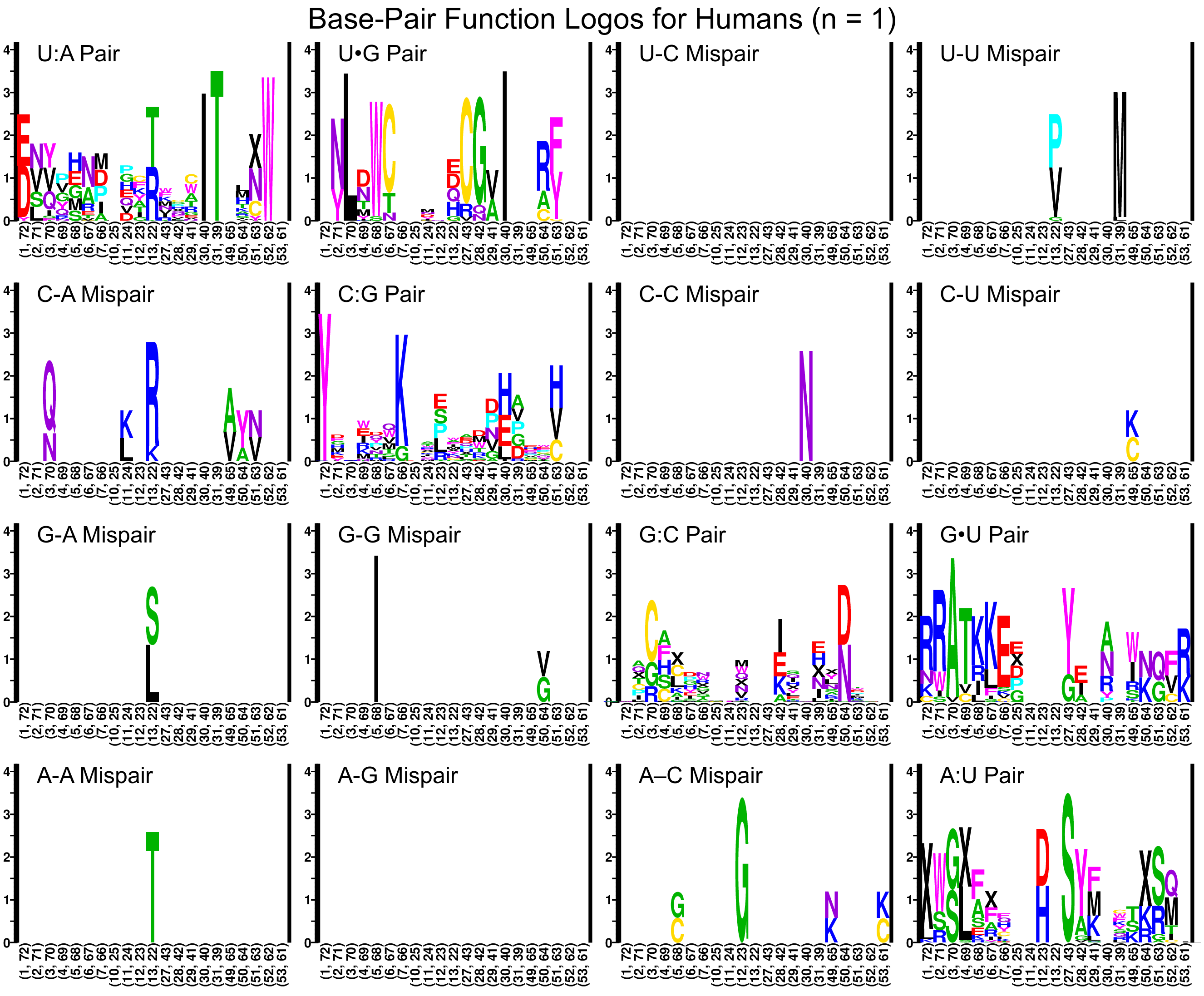

Supplement: S32 Fig — (PNG) [file pntd.0007983.s032.png]

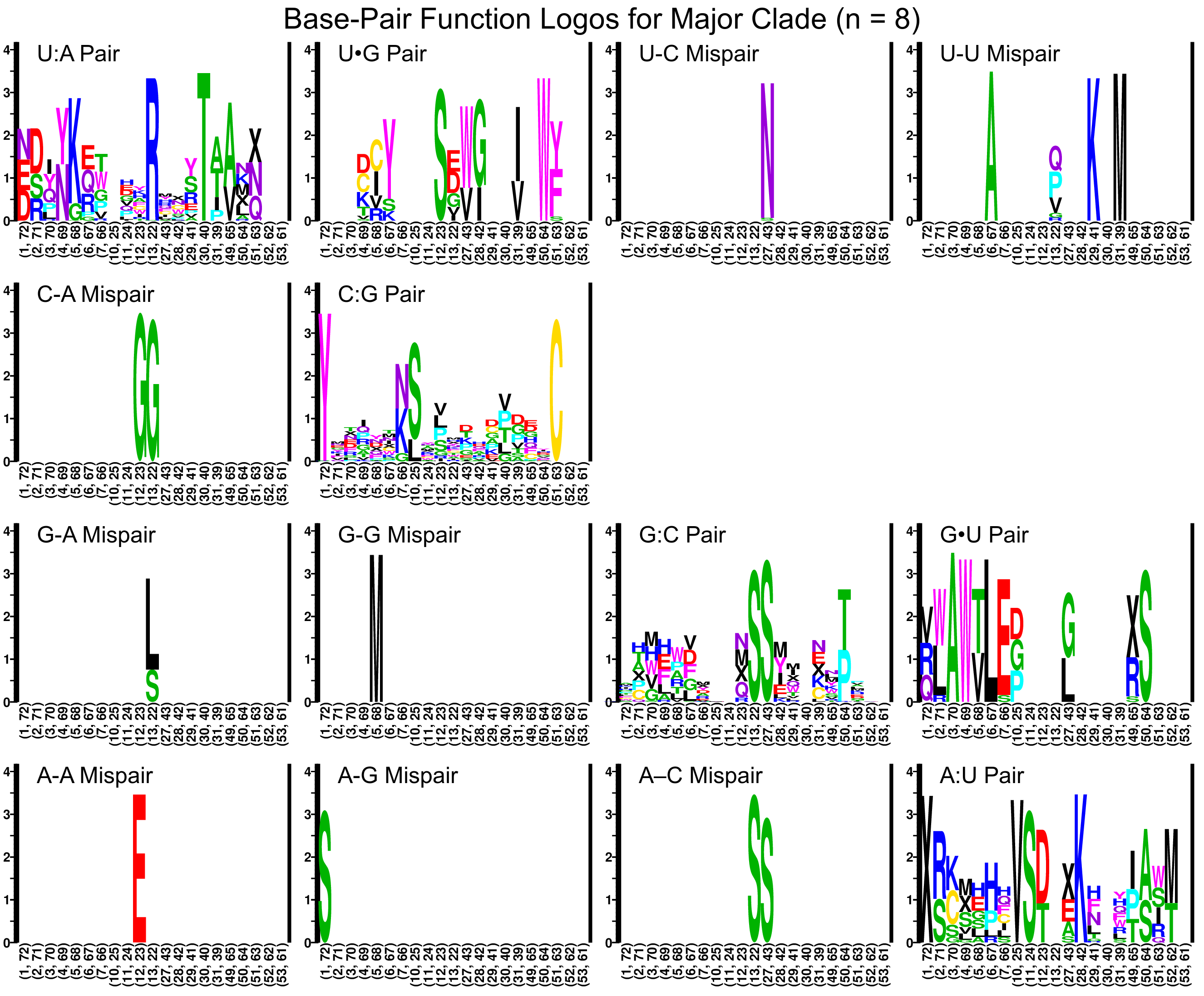

Supplement: S33 Fig — (PNG) [file pntd.0007983.s033.png]

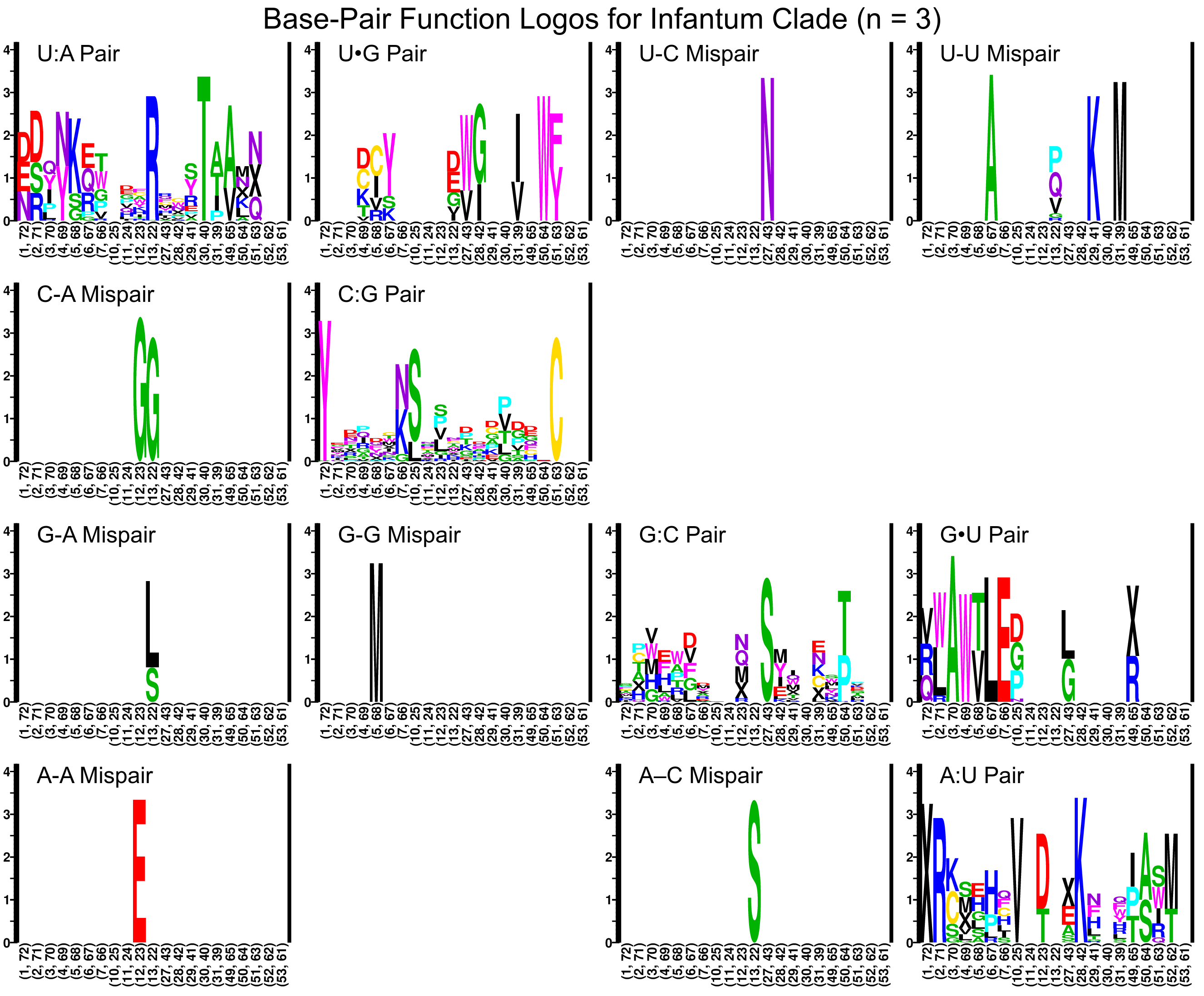

Supplement: S34 Fig — (PNG) [file pntd.0007983.s034.png]

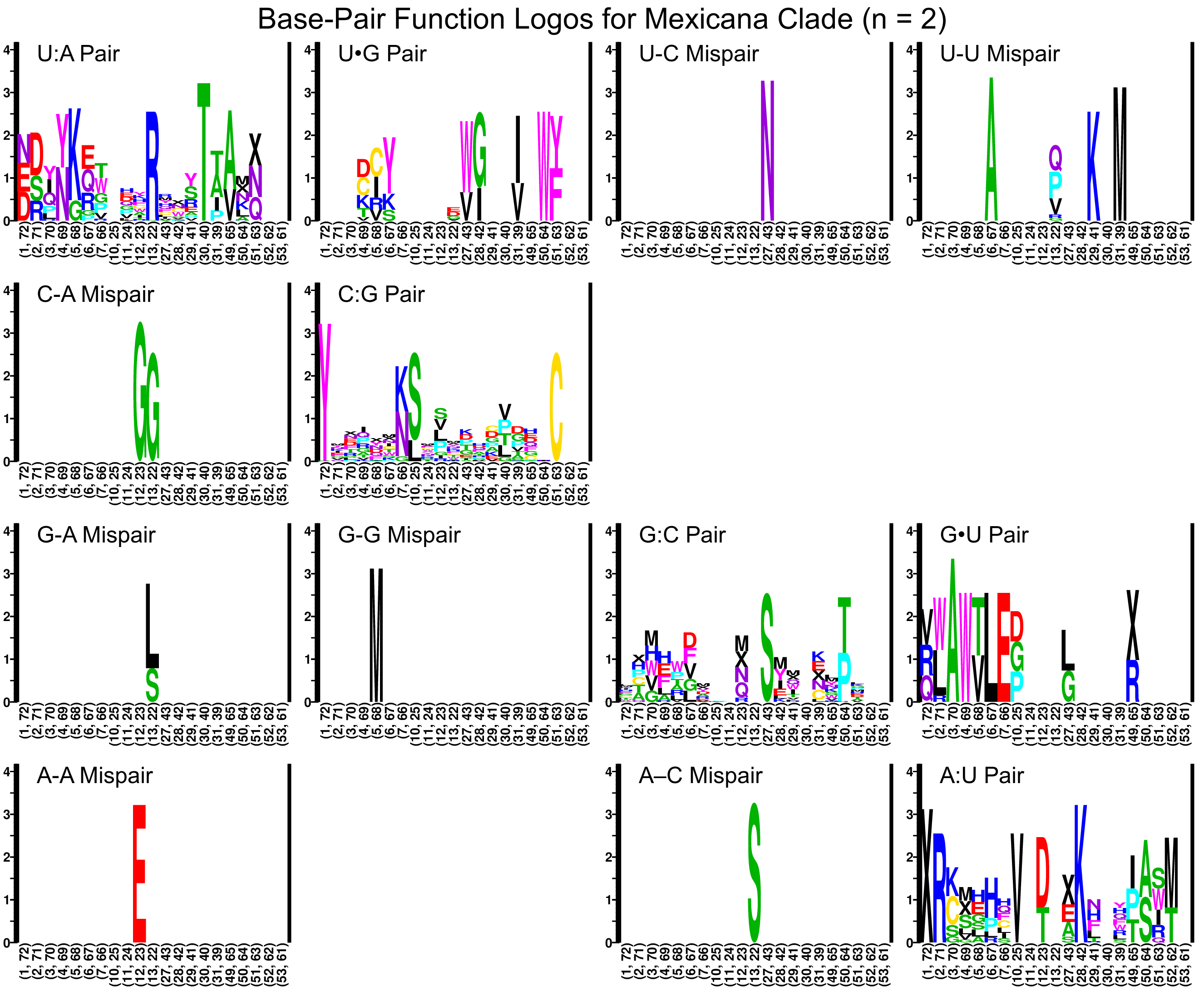

Supplement: S35 Fig — (PNG) [file pntd.0007983.s035.png]

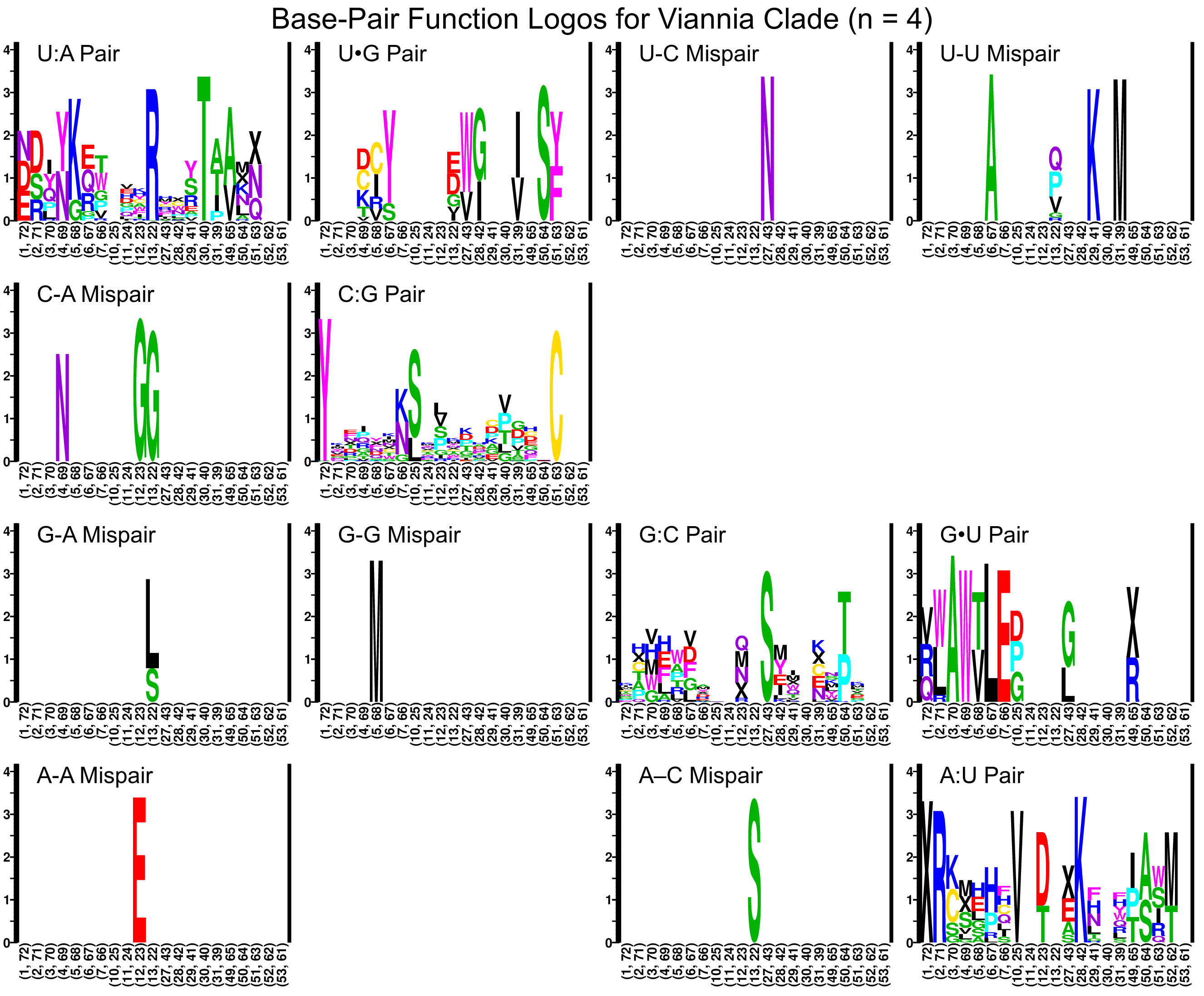

Supplement: S36 Fig — (PNG) [file pntd.0007983.s036.png]

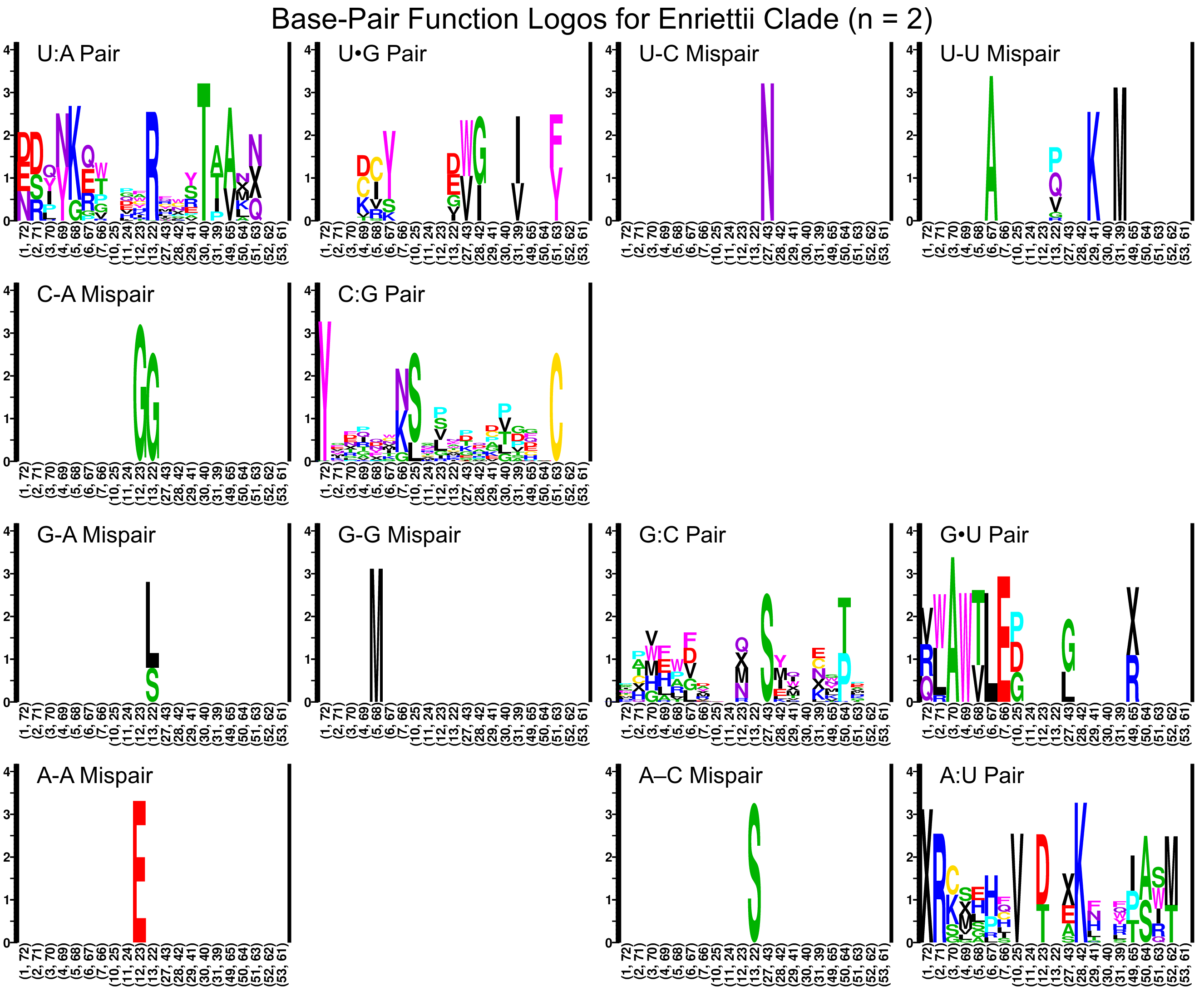

Supplement: S37 Fig — (PNG) [file pntd.0007983.s037.png]

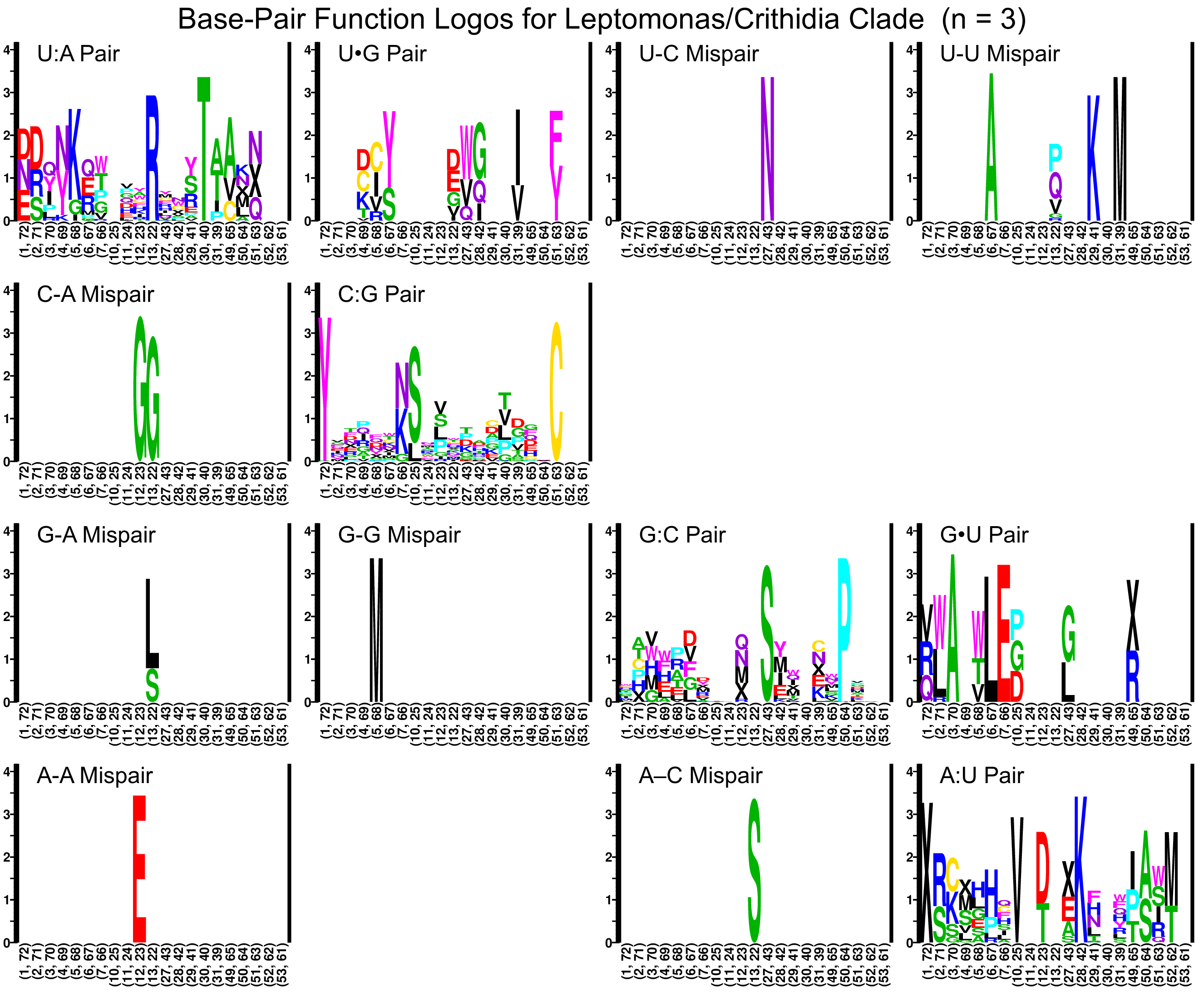

Supplement: S38 Fig — (PNG) [file pntd.0007983.s038.png]

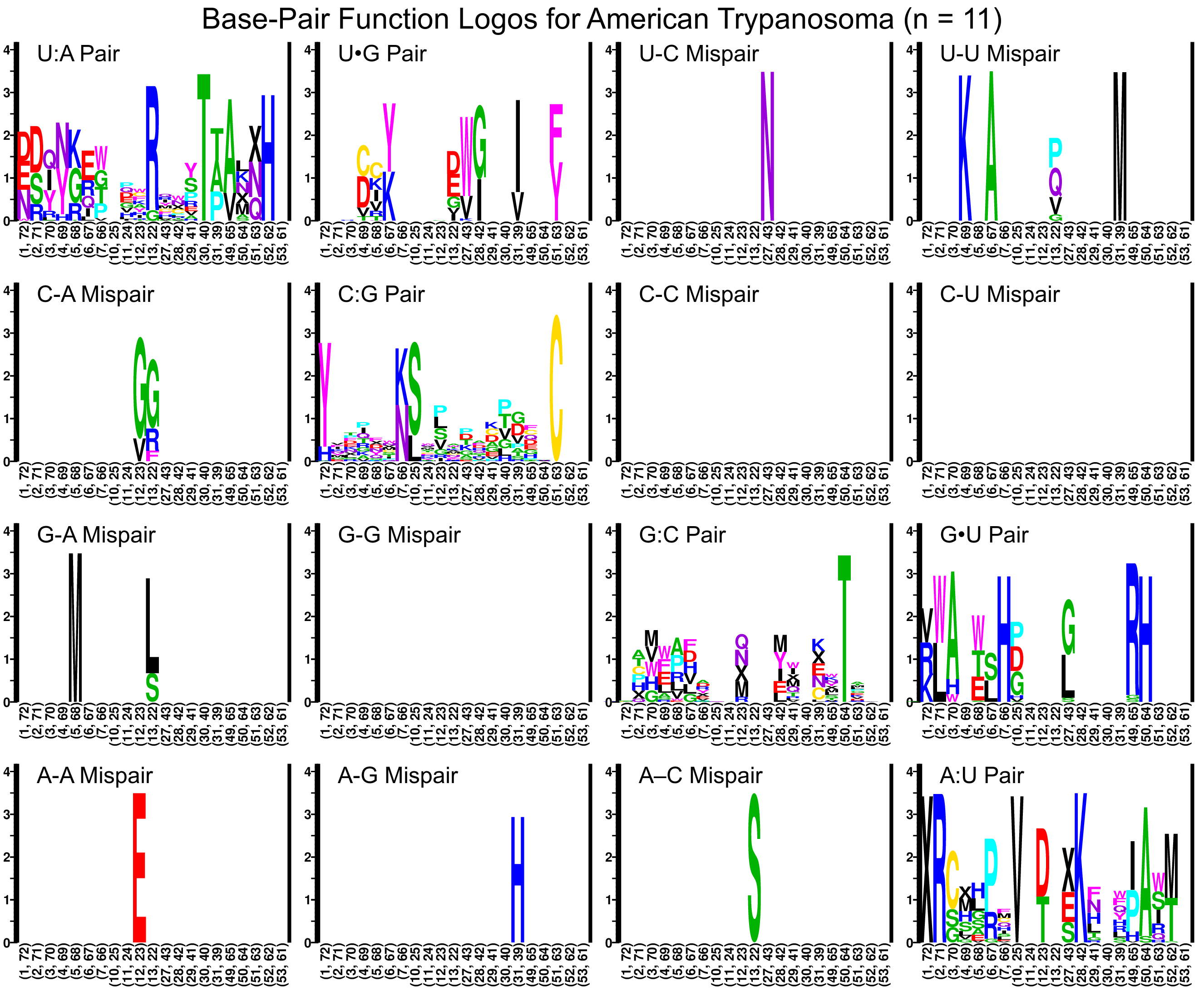

Supplement: S39 Fig — (PNG) [file pntd.0007983.s039.png]

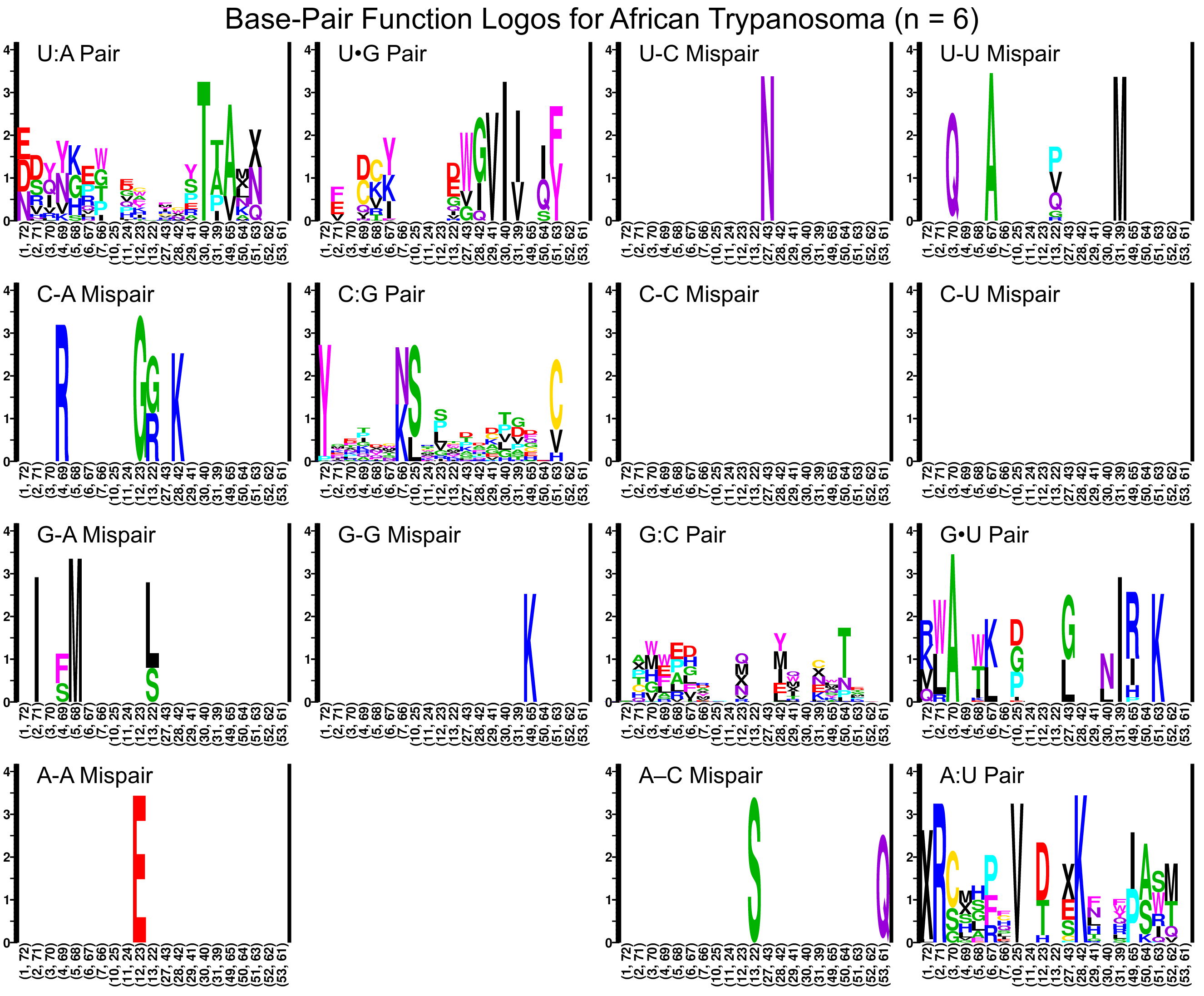

Supplement: S40 Fig — (PNG) [file pntd.0007983.s040.png]

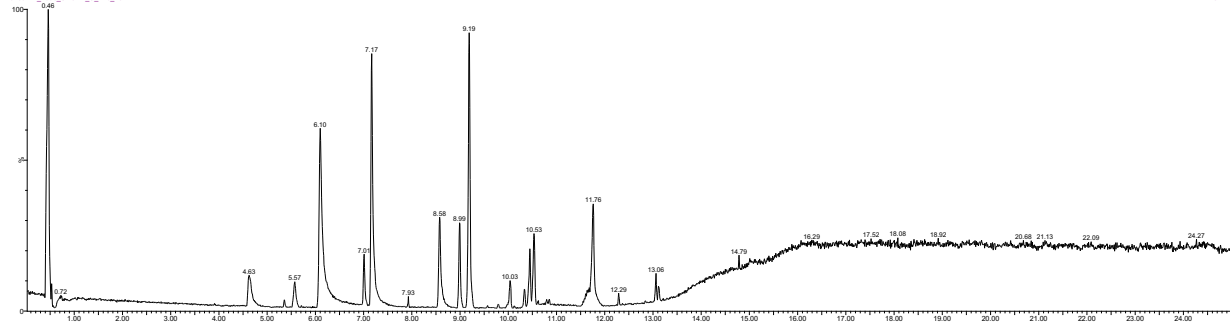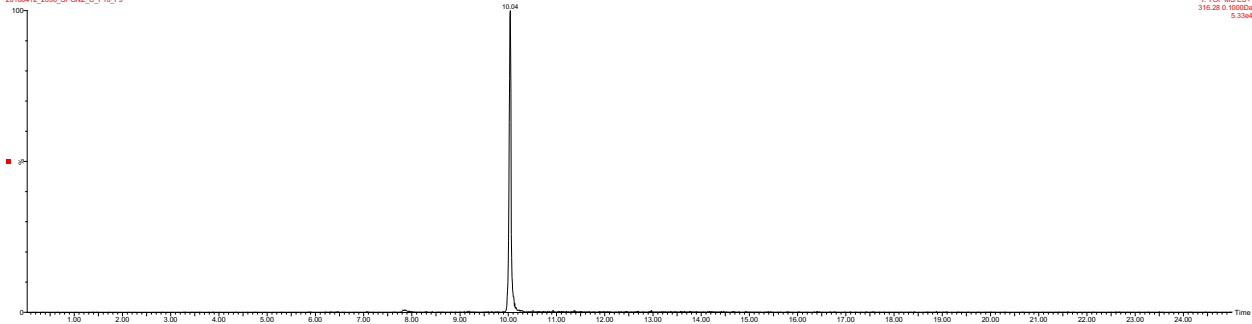

Supplement: S41 Fig — UPLC-qTOF base peak chromatogram of (A) RL12-182-HVF-D Sep-Pak Fraction C Subfraction 10–9 and (B) extracted ion chromatogram of peak 316.28 m/z in active region of trace. (PDF) [file pntd.0007983.s041.pdf]

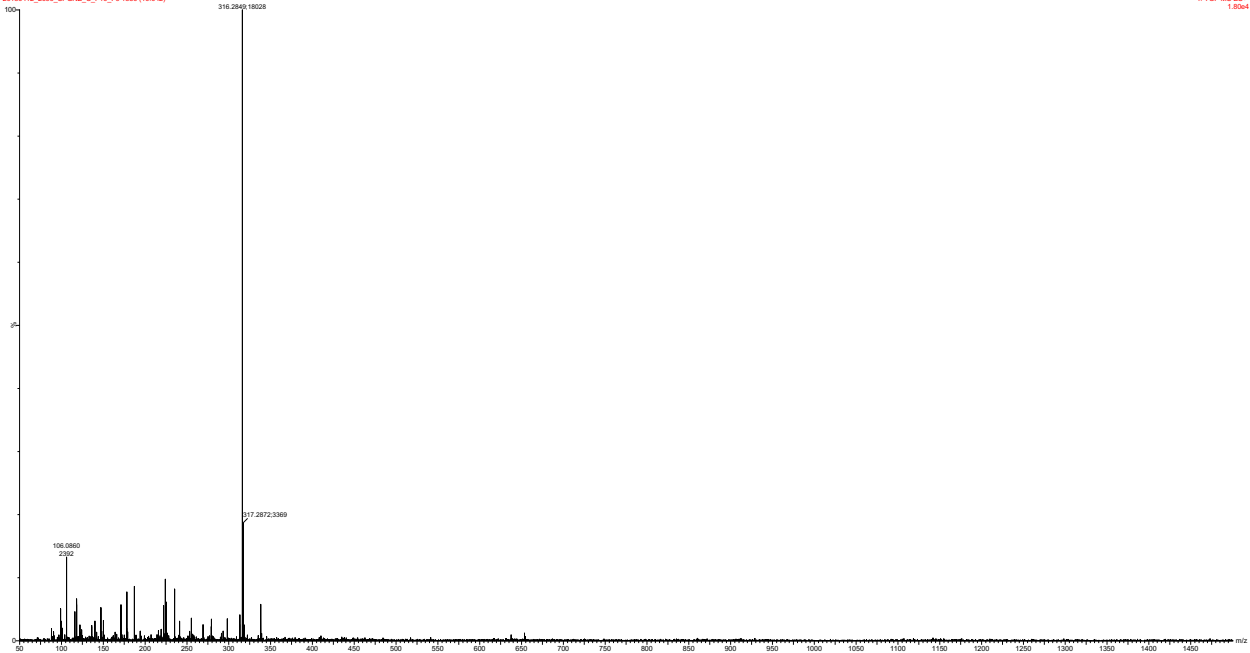

Supplement: S42 Fig — (PDF) [file pntd.0007983.s042.pdf]

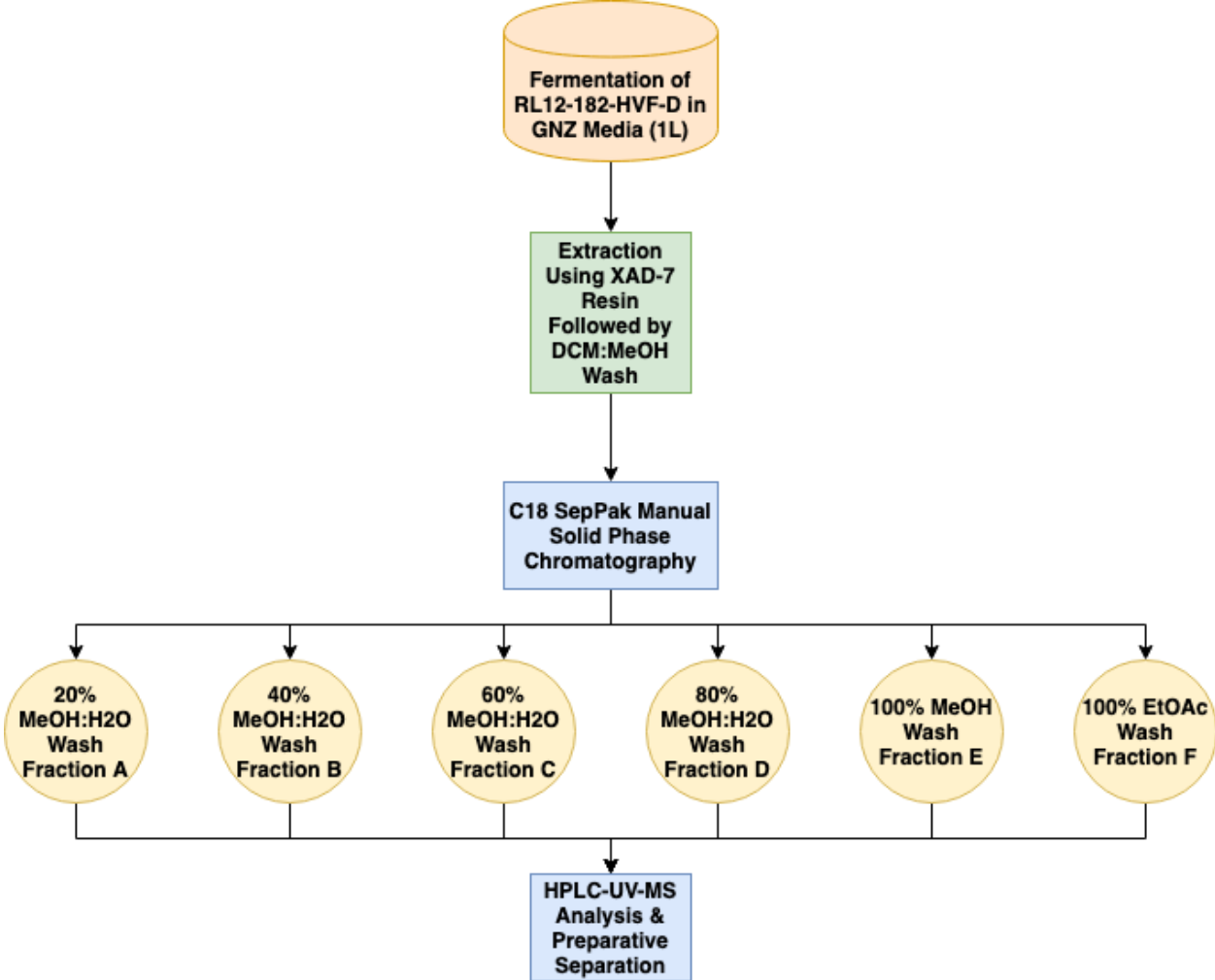

Supplement: S43 Fig — (PDF) [file pntd.0007983.s043.pdf]

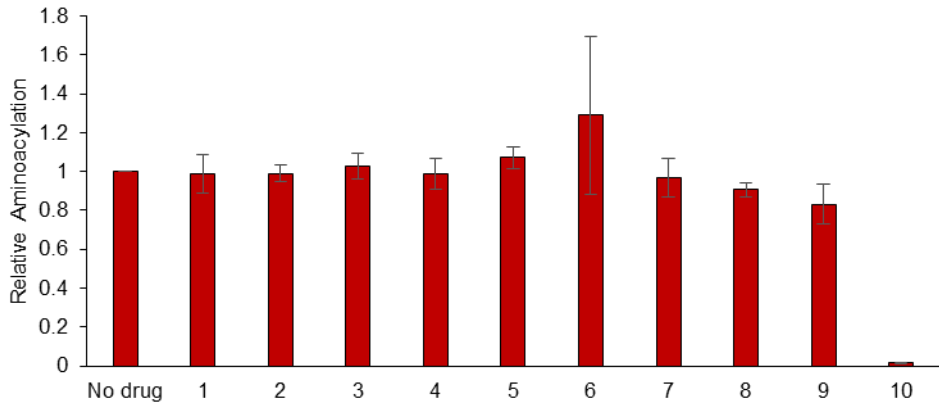

Supplement: S45 Fig — (PDF) [file pntd.0007983.s045.pdf]

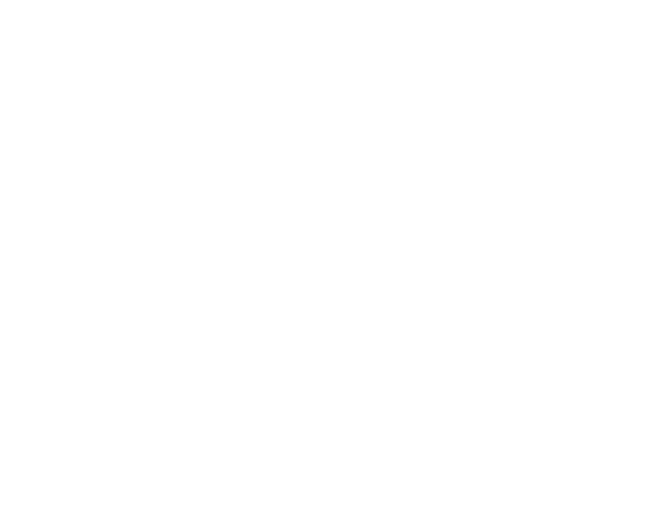

Supplement: S1 Code and Data — (TGZ) [file pntd.0007983.s052.tgz › KellyEtAl_PLoS_NTD_SOM/null.png]
