# Supplementary material for: Targeting tRNA-synthetase interactions towards novel therapeutic discovery against eukaryotic pathogens
Source: PLoS Negl Trop Dis. 2020 Feb 27;14(2):e0007983. doi: 10.1371/journal.pntd.0007983 (PMC7046186; doi:10.1371/journal.pntd.0007983)
Supplement: S44 Fig — Red box denotes active peak. (PDF) [file pntd.0007983.s044.pdf]

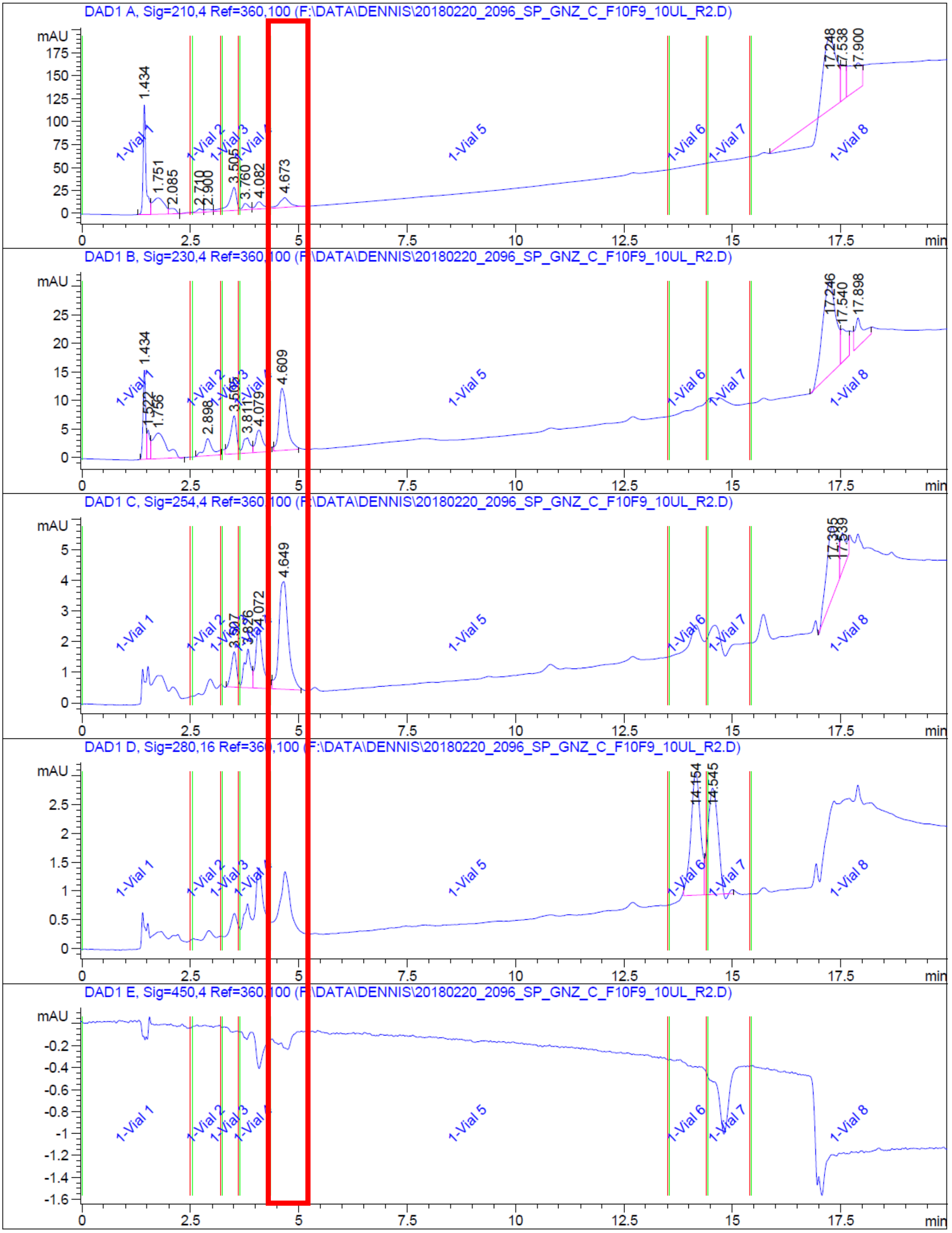
DAD1 A, Sig=210,4 Ref=360,100 (F:\DATA\DENNIS\20180220_2096_SP_GNZ_C_F10F9_10UL_R2.D)
DAD1 B, Sig=230,4 Ref=360,100 (F:\DATA\DENNIS\20180220_2096_SP_GNZ_C_F10F9_10UL_R2.D)
DAD1 C, Sig=254,4 Ref=360,100 (F:\DATA\DENNIS\20180220_2096_SP_GNZ_C_F10F9_10UL_R2.D)
DAD1 D, Sig=280,16 Ref=360,100 (F:\DATA\DENNIS\20180220_2096_SP_GNZ_C_F10F9_10UL_R2.D)
DAD1 E, Sig=450,4 Ref=360,100 (F:\DATA\DENNIS\20180220_2096_SP_GNZ_C_F10F9_10UL_R2.D)
mAU
min
1-Vial 1
1-Vial 2
1-Vial 3
1-Vial 5
1-Vial 6
1-Vial 7
1-Vial 8
