# Supplementary material for: Targeting tRNA-synthetase interactions towards novel therapeutic discovery against eukaryotic pathogens
Source: PLoS Negl Trop Dis. 2020 Feb 27;14(2):e0007983. doi: 10.1371/journal.pntd.0007983 (PMC7046186; doi:10.1371/journal.pntd.0007983)
Supplement: S1 Table — (PDF) [file pntd.0007983.s046.pdf]

Supplementary Table 1 —Similar and putatively homologous tRNA gene cluster variant groups of length at least three occurring in at least two *Leishmania* genomes.

Group	Gene Cluster Variant / Strandedness	Frequency
1	**DSA / – – +**	21
2	**GAL / – – +**	7
2	**LAG / – + +**	10
3	**KGN / + – –**	13
4	**KVP / – – –**	2
4	**PVK +++**	20
5	**LXP / – – +**	5
5	**CLXP / + – – +**	16
6	**MRR / – + +**	2
7	**QLI / + – +**	19
7	**IVQRLTRKGW / + – – – – – + + – +**	18
8	**RRA / – – +**	19
8	**EARR / – – + +**	2
9	**TGP / – – +**	2
9	**GTGP / + – – +**	12
9	**PGTTG / – + + – –**	4
10	**VHF / + + –**	15
10	**EVRH / – – + +**	21
11	**YTTT / – – – +**	2
11	**YTTY / – – + +**	8
12	**NPTYN / – – + + +**	8
12	**NYTPN / – – – + +**	14
13	**SLMIV / – + – – +**	3
13	**VIMLS / – + + – +**	18
