# Supplementary material for: Targeting tRNA-synthetase interactions towards novel therapeutic discovery against eukaryotic pathogens
Source: PLoS Negl Trop Dis. 2020 Feb 27;14(2):e0007983. doi: 10.1371/journal.pntd.0007983 (PMC7046186; doi:10.1371/journal.pntd.0007983)
Supplement: S2 Table — (PDF) [file pntd.0007983.s047.pdf]

Supplementary Table 2 — Similar and putatively homologous tRNA gene cluster variant groups of length at least three occurring in at least two *Trypanosoma* genomes.

Group	Gene Cluster Variant / Strandedness	Frequency
1	**ARF / – – +**	10
1	**FRA / – + +**	5
2	**ASD / – – +**	7
2	**DSA / – + +**	14
3	**FEV / – – +**	5
3	**HEF / + + +**	6
3	**VEF / – + +**	7
4	**IQL / – + +**	7
4	**LQI / – – +**	6
4	**ILQQI / + – – + +**	3
5	**LXP / – – +**	6
5	**LXP / + – +**	5
5	**PXL / – + +**	6
6	**NTP / – – +**	2
6	**PTN / – + +**	8
7	**SLS / + – –**	4
8	**VLM / – – –**	5
8	**EMSV / – + – +**	4
8	**LSMEMYV / – + – – + – +**	2
8	**LSMEMYV / – + – + + – +**	3
8	**VYMEMSL / – + – – + – +**	2
9	**GLTR / + + – +**	6
9	**IQVKGLTRKR / – + + + + – – + + –**	7
9	**RKRTLGKVQI / + – – + + – – – – +**	3
10	**IQVK / – + + +**	4
11	**NARK / + – + +**	2
11	**NARKR / + – + + +**	11
