# Supplementary material for: Targeting tRNA-synthetase interactions towards novel therapeutic discovery against eukaryotic pathogens
Source: PLoS Negl Trop Dis. 2020 Feb 27;14(2):e0007983. doi: 10.1371/journal.pntd.0007983 (PMC7046186; doi:10.1371/journal.pntd.0007983)
Supplement: S3 Table — Only groups 1, 3 and 4 contain five gene cluster variants conserved in both genera, namely, ASD, DSA, LXP (two variants), and QLI, where “X” represents the initiator iMet tRNA gene. (PDF) [file pntd.0007983.s048.pdf]

Supplementary Table 3 — Similar and putatively homologous tRNA gene cluster variant groups of length at least three spanning both *Leishmania* (L) and *Trypanosoma* (T) genera genomes. Only groups 1, 3 and 4 contain five gene cluster variants conserved in both genera, namely, ASD, DSA, LXP (two variants), and QLI, where "X" represents the initiator iMet tRNA gene.

Group	Gene Cluster Variant / Strandedness	Frequency	Clade
1	**ARF / – – +**	10	T
1	**FRA / – + +**	5	T
1	**RRA / – – +**	19	L
1	**EARR / – – + +**	2	L
2	**ASD / – – +**	8	L, T
2	**DSA / – + +**	35	L, T
3	**FEV / – – +**	5	T
3	**HEF / + + +**	6	T
3	**VEF / – + +**	7	T
3	**VHF / + + –**	15	L
3	**EVRH / – – + +**	21	L
4	**IQL / – + +**	7	T
4	**LQI / – – +**	6	T
4	**QLI / + – +**	19	L, T
4	**ILQQI / + – – + +**	3	T
5	**LXP / – – +**	11	L, T
5	**LXP / + – +**	5	L, T
5	**PXL / – + +**	6	T
5	**CLXP / + – – +**	16	L
6	**NTP / – – +**	2	T
6	**PTN / – + +**	8	T
6	**NPTYN / – – + + +**	8	L
6	**NYTPN / – – – + +**	14	L
7	**VLM / – – –**	5	T
7	**SLMIV / – + – – +**	3	L
7	**VIMLS / – + + – +**	18	L
8	**GLTR / + + – +**	6	T
8	**IQVKGLTRKR / – + + + + – – + + –**	7	T
8	**IVQRLTRKGW / + – – – – – + + – +**	18	L
8	**RKRTLGKVQI / + – – + + – – – – +**	3	T
