# Supplementary material for: Targeting tRNA-synthetase interactions towards novel therapeutic discovery against eukaryotic pathogens
Source: PLoS Negl Trop Dis. 2020 Feb 27;14(2):e0007983. doi: 10.1371/journal.pntd.0007983 (PMC7046186; doi:10.1371/journal.pntd.0007983)
Supplement: S4 Table — (PDF) [file pntd.0007983.s049.pdf]

Supplementary Table 4 — Gene Length, Structure and Functional Type Statistics on Final Annotation Gene Sets

	Intersection Set	Aragorn–Only	Union Set
**#tRNA genes**	3579	36	3616
**Avg. gene length**	74	98	75
**Min. gene length**	68	71	68
**Max. gene length**	89	206	206
**%intron**	2	28	3
**%GC**	58	59	58
**#Ala tRNA genes**	210	2	212
**#Cys tRNA genes**	64	1	65
**#Asp tRNA genes**	105	1	106
**#Glu tRNA genes**	160	1	161
**#Phe tRNA genes**	104	2	106
**#Gly tRNA genes**	228	3	231
**#His tRNA genes**	80	4	84
**#Ile tRNA genes**	171	1	172
**#Lys tRNA genes**	183	1	184
**#Leu tRNA genes**	335	6	341
**#Met tRNA genes**	97	0	97
**#Asn tRNA genes**	125	0	125
**#Pro tRNA genes**	200	0	200
**#Gln tRNA genes**	161	0	161
**#Arg tRNA genes**	348	2	350
**#Ser tRNA genes**	228	7	235
**#Thr tRNA genes**	218	4	222
**#Val tRNA genes**	236	0	236
**#Trp tRNA genes**	52	1	53
**#iMet tRNA genes**	76	0	76
**#Tyr tRNA genes**	88	0	88
**#SeC tRNA genes**	76	0	76
**#Ambiguous tRNA genes**	34	0	35
