# Supplementary material for: Targeting tRNA-synthetase interactions towards novel therapeutic discovery against eukaryotic pathogens
Source: PLoS Negl Trop Dis. 2020 Feb 27;14(2):e0007983. doi: 10.1371/journal.pntd.0007983 (PMC7046186; doi:10.1371/journal.pntd.0007983)
Supplement: S5 Table — (PDF) [file pntd.0007983.s050.pdf]

Supplementary Table 5 — Nucleotide Composition of TriTryp tRNA Gene–Sets by Individual Genome Assembly, Organized by Clade (alternating background in the order of Table 1, boldface) or excluded (at end of table in roman font)

Genome	Genes	Pooled %G	Pooled %C	Pooled %T
**LaethiopicaL147**	**83**	**32.0**	**26.1**	**23.4**
**LarabicaLEM1108**	**85**	**31.8**	**26.0**	**23.5**
**LgerbilliLEM452**	**81**	**31.9**	**26.2**	**23.3**
**LmajorFriedlin**	**84**	**32.0**	**26.1**	**23.4**
**LmajorLV39c5**	**84**	**31.9**	**26.1**	**23.4**
**LmajorSD75**	**82**	**32.0**	**26.1**	**23.4**
**LtropicaL590**	**87**	**31.9**	**26.0**	**23.4**
**LturanicaLEM423**	**86**	**31.9**	**26.1**	**23.3**
**LdonovaniBPK282A1**	**85**	**31.9**	**26.1**	**23.3**
**LdonovaniBHU1220**	**84**	**31.9**	**26.1**	**23.3**
**LinfantumJPCM5**	**84**	**31.9**	**26.0**	**23.4**
**LmexicanaMHOMGT2001U1103**	**84**	**31.9**	**26.0**	**23.4**
**LamazonensisMHOMBR71973M2269**	**66**	**31.8**	**25.9**	**23.4**
**LbraziliensisMHOMBR75M2903**	**86**	**31.8**	**26.0**	**23.5**
**LbraziliensisMHOMBR75M2904**	**83**	**32.0**	**26.1**	**23.4**
**LpanamensisMHOMCOL81L13**	**88**	**31.8**	**26.2**	**23.3**
**LpanamensisMHOMPA94PSC1**	**74**	**31.9**	**26.1**	**23.3**
**LenriettiiLEM3045**	**82**	**31.9**	**26.0**	**23.4**
**LspMARLEM2494**	**80**	**31.9**	**25.9**	**23.4**
**CfasciculataCfCl**	**105**	**31.8**	**26.0**	**23.4**
**LpyrrhocorisH10**	**104**	**31.9**	**26.1**	**23.3**
**LseymouriATCC30220**	**94**	**31.9**	**26.0**	**23.3**
**TcruziCLBrenerEsmeraldo-like**	**57**	**32.3**	**26.3**	**23.1**
**TcruziCLBrenerNon-Esmeraldo-like**	**57**	**32.2**	**26.4**	**22.9**

**TcruzimarinkelleiB7**	**57**	**32.3**	**26.5**	**22.8**
**TcruziDm28c**	**51**	**32.3**	**26.4**	**22.8**
**TcruzicruziDm28c**	**97**	**32.5**	**26.3**	**23.0**
**TcruziEsmeraldo**	**74**	**32.2**	**26.2**	**22.8**
**TcruziJRcl4**	**74**	**32.2**	**26.0**	**23.0**
**TcruziTulacl2**	**121**	**32.2**	**26.4**	**23.0**
**TcruziSylvioX10-1**	**69**	**32.2**	**26.2**	**23.1**
**TcruziSylvioX10-1-2012**	**72**	**32.1**	**26.4**	**22.8**
**TgrayiANR4**	**95**	**32.5**	**26.3**	**23.0**
**TbruceigambienseDAL972**	**64**	**32.7**	**26.4**	**22.8**
**TbruceiLister427**	**67**	**32.6**	**26.4**	**22.9**
**TbruceiTREU927**	**73**	**32.5**	**26.3**	**22.9**
**TcongolenseIL3000**	**72**	**32.7**	**26.5**	**22.9**
**TevansiSTIB805**	**67**	**32.6**	**26.4**	**22.9**
**TvivaxY486**	**82**	**31.0**	**25.2**	**23.9**
TtheileriEdinburgh	159	32.2	26.3	22.7
EmonterogeiiLV88	104	31.6	26.1	23.2
BayalaiB08-376	69	31.6	26.3	23.1
LtarentolaeParrotTarII	79	31.8	26.0	23.4
PconfusumCUL13	61	32.2	26.2	22.9
TrangeliSC58	6	29.4	25.3	24.0
TcruziCLBrener	18	33.3	26.4	22.9
