# Supplementary material for: Targeting tRNA-synthetase interactions towards novel therapeutic discovery against eukaryotic pathogens
Source: PLoS Negl Trop Dis. 2020 Feb 27;14(2):e0007983. doi: 10.1371/journal.pntd.0007983 (PMC7046186; doi:10.1371/journal.pntd.0007983)
Supplement: S6 Table — (PDF) [file pntd.0007983.s051.pdf]

Supplementary Table 6 — Values of four replicate count-per-minute endpoints (taken at time *t* = 10 min. in time-course measurements as in Fig. 9C) of *Leishmania major* AlaRS under DMSO or without added enzyme, their means and standard deviations, and their corresponding Z-factor in the scintillation counter-based aminoacylation time-course assay.

Replicate	DMSO Endpoint	No Enzyme Endpoint
1	2674	25
2	2844	20
3	2814	27
4	3047	326
**mean**	2844.75	99.5
**std. dev.**	153.8	151.03
Z′	**0.67**	
