# Supplementary material for: Targeting tRNA-synthetase interactions towards novel therapeutic discovery against eukaryotic pathogens
Source: PLoS Negl Trop Dis. 2020 Feb 27;14(2):e0007983. doi: 10.1371/journal.pntd.0007983 (PMC7046186; doi:10.1371/journal.pntd.0007983)
Supplement: S1 Code and Data — (TGZ) [file pntd.0007983.s052.tgz › KellyEtAl_PLoS_NTD_SOM/KellyEtAl_PLoS_NTD_SOM.html]

KellyEtAl\_PLoS\_NTD\_SOM


# Supplementary Online Methods for Kelly *Et Al.* "Targeting tRNA-Synthetase Interactions towards Novel Therapeutic Discovery Against Eukaryotic Pathogens"¶

## About this Document¶

This document was created from the Jupyter notebook `KellyEtAl_PLoS_NTD_SOM.ipynb`, written by David Ardell, using a BASH kernel. It contains code to reproduce the figures from:

"Targeting tRNA-Synthetase Interactions towards Novel Therapeutic Discovery Against Eukaryotic Pathogens" by Paul Kelly, Fatemeh Hadi-Nezhad, Dennis Liu, Travis J. Lawrence, Roger G. Linington, Michael Ibba, David H. Ardell

This version uses the Bayesian Nemenman–Shafee–Bialek (NSB) entropy estimator with option -x 1 (which calculates the expected bias by the method of Schneider et al. (1996) in a sample size of 1).

## Copyright, License, Sources and Credits for this SOM¶

Please see the files `COPYING`, `GNULICENSE`, and `README.md` that should be distributed together with this file.

## Required Software to Reproduce Results and Figures¶

- cove (http://eddylab.org/software.html)
- tSFM >= v0.9.14 (https://github.com/tlawrence3/tSFM)
- ImageMagick (https://imagemagick.org/index.php)
- FAST (https://github.com/tlawrence3/FAST)
- Packages
  - Set::Scalar (perl)
  - gdata (R)
  - heR.Misc (R) (http://exposurescience.org/her.html)

## Test tSFM and FAST versions¶

In [1]:

```
tsfm -V
fasgrep --version
```

```
tsfm v0.9.14
/usr/local/bin/fasgrep version 1.06
(Getopt::Long::GetOptions version 2.39; Perl version 5.18.2)
```

## Score-Filtered Final Gene Annotation for TriTrypDB v.41¶

This is includes all genes with tRNAscan-SE scores >= 50 bits or Aragorn scores >= 107 bits in 46 genomes.

In [4]:

```
faswc TriTrybDB_v41.Hadi-Nezhad_Ardell_Annotation_KellyEtAl19.fas 
cat TriTrybDB_v41.Hadi-Nezhad_Ardell_Annotation_KellyEtAl19.fas | grep \> | perl -pe 's/_.*//' | sort | uniq -c | wc
cat TriTrybDB_v41.Hadi-Nezhad_Ardell_Annotation_KellyEtAl19.fas | grep \> | perl -pe 's/_.*//' | sort | uniq -c
```

```
      3616    270146 TriTrybDB_v41.Hadi-Nezhad_Ardell_Annotation_KellyEtAl19.fas
      3616    270146 total
      46      92    1103
  69 >BayalaiB08-376
 105 >CfasciculataCfCl
 104 >EmonterogeiiLV88
  83 >LaethiopicaL147
  66 >LamazonensisMHOMBR71973M2269
  85 >LarabicaLEM1108
  86 >LbraziliensisMHOMBR75M2903
  83 >LbraziliensisMHOMBR75M2904
  84 >LdonovaniBHU1220
  85 >LdonovaniBPK282A1
  82 >LenriettiiLEM3045
  81 >LgerbilliLEM452
  84 >LinfantumJPCM5
  84 >LmajorFriedlin
  84 >LmajorLV39c5
  82 >LmajorSD75
  84 >LmexicanaMHOMGT2001U1103
  88 >LpanamensisMHOMCOL81L13
  74 >LpanamensisMHOMPA94PSC1
 104 >LpyrrhocorisH10
  94 >LseymouriATCC30220
  80 >LspMARLEM2494
  79 >LtarentolaeParrotTarII
  87 >LtropicaL590
  86 >LturanicaLEM423
  61 >PconfusumCUL13
  67 >TbruceiLister427
  73 >TbruceiTREU927
  64 >TbruceigambienseDAL972
  72 >TcongolenseIL3000
  18 >TcruziCLBrener
  57 >TcruziCLBrenerEsmeraldo-like
  57 >TcruziCLBrenerNon-Esmeraldo-like
  51 >TcruziDm28c
  74 >TcruziEsmeraldo
  74 >TcruziJRcl4
  69 >TcruziSylvioX10-1
  72 >TcruziSylvioX10-1-2012
 121 >TcruziTulacl2
  97 >TcruzicruziDm28c
  57 >TcruzimarinkelleiB7
  67 >TevansiSTIB805
  95 >TgrayiANR4
   6 >TrangeliSC58
 159 >TtheileriEdinburgh
  82 >TvivaxY486
```

## Initial Data: Editing Alignment, Mapping to Sprinzl Coordinates, and Selecting Sites¶

Tthe file `final_CIF_geneset.fasta` as a starting point for TriTryp data. This file contains 3488 genes from 44 genomes, excluding two incomplete assemblies for TcruziCLBrener and TrangeliSC58. Identifiers end with the gene class (3454, intersection set) or `ara` and the gene class (34 aragorn-only). We filter the human data (433 genes) in `hg38-tRNAs.fa` from GtRNAdb for Selenocysteine genes, resulting in 431 human tRNA genes. Combined data held 3919 gene sequences. To make it easier to edit alignments for Type II (L,S,E) sequences that are misaligned at positions 45 and 47, DHA sorted by sequence character at position 196 in the COVEA produced alignment. DHA then edited 595 sequences in Seaview, as well as selected 74 sites, saving the work in the SEAVIEW file `tritryp_homo.covea.sort.edited.mase`. The steps to reproduce the edited alignment are below and we include the edited alignment of 3919 genes in `covea.74sites.fas`.

The code chunk to produce the input data was as follows

```
fasgrep -vd SeC hg38-tRNAs.fa > hg38-tRNAs-noSeC.fas  # Remove Two SeC sequences from hg38-tRNAs.fa leaving 431 Human sequences
cat final_CIF_geneset.fasta hg38-tRNAs-noSeC.fas > tritryp_homo.fas
covea -o tritryp_homo.covea TRNA2-euk.cm tritryp_homo.fas
sreformat -u a2m tritryp_homo.covea | tr '[a-z].' '[A-Z]-' > tritryp_homo.covea.fas
fassort -sx '.{195}(.)' tritryp_homo.covea.fas | fascut --moltype=DNA 196 | fasgrep -s "-" | faswc # 595 Human and kinetoplastid sequences have a gap at coordinate 45, start of V-arm, typically type II L or S, one E
fassort -sx '.{195}(.)' tritryp_homo.covea.fas > tritryp_homo.covea.sort.fas
# edit alignment in seaview and save file in tritryp_homo.covea.sort.edited.mase to manually edit misaligned coordinates 45 and 47
# extract 74 site alignment in seaview into fasta format
# compare 74-site alignments from v5,v6 for equality to current 74-site alignment
fassub -d '.*' '' tritryp_homo.covea.sort.edited.74sites.fas > covea.74sites.fas # remove "no comment" in description
```

In [1]:

```
faswc *.fa* 
#faswc final_CIF_geneset.fasta #3488
#faswc covea.74sites.fas #3919
```

```
      3616    270146 TriTrybDB_v41.Hadi-Nezhad_Ardell_Annotation_KellyEtAl19.fas
      3919    290006 covea.74sites.fas
      3488    259536 final_CIF_geneset.fas
       431     32403 hg38-tRNAs-noSeC.fas
       433     32574 hg38-tRNAs.fa
     11887    884665 total
```

### Genomes in Gene Set and Gene Numbers by Genome¶

Among 46 genomes downloaded geneset

In [43]:

```
cat final_CIF_geneset.fasta | grep \> | perl -pe 's/_.*//' | sort | uniq -c 
cat final_CIF_geneset.fasta | grep \> | perl -pe 's/_.*//' | sort | uniq -c | wc
```

```
  68 >BayalaiB08-376
 104 >CfasciculataCfCl
 102 >EmonterogeiiLV88
  82 >LaethiopicaL147
  65 >LamazonensisMHOMBR71973M2269
  84 >LarabicaLEM1108
  85 >LbraziliensisMHOMBR75M2903
  82 >LbraziliensisMHOMBR75M2904
  83 >LdonovaniBHU1220
  84 >LdonovaniBPK282A1
  81 >LenriettiiLEM3045
  80 >LgerbilliLEM452
  83 >LinfantumJPCM5
  83 >LmajorFriedlin
  83 >LmajorLV39c5
  81 >LmajorSD75
  83 >LmexicanaMHOMGT2001U1103
  87 >LpanamensisMHOMCOL81L13
  73 >LpanamensisMHOMPA94PSC1
 103 >LpyrrhocorisH10
  93 >LseymouriATCC30220
  79 >LspMARLEM2494
  77 >LtarentolaeParrotTarII
  86 >LtropicaL590
  85 >LturanicaLEM423
  61 >PconfusumCUL13
  64 >TbruceiLister427
  70 >TbruceiTREU927
  61 >TbruceigambienseDAL972
  65 >TcongolenseIL3000
  55 >TcruziCLBrenerEsmeraldo-like
  56 >TcruziCLBrenerNon-Esmeraldo-like
  49 >TcruziDm28c
  73 >TcruziEsmeraldo
  67 >TcruziJRcl4
  61 >TcruziSylvioX10-1
  65 >TcruziSylvioX10-1-2012
 117 >TcruziTulacl2
  88 >TcruzicruziDm28c
  52 >TcruzimarinkelleiB7
  64 >TevansiSTIB805
  90 >TgrayiANR4
 156 >TtheileriEdinburgh
  78 >TvivaxY486
      44      88    1063
```

### Table 1: Numbers and Compositions of Genes by Defined Clades¶

Here we compute gene numbers and nucleotide compositions for Table 1 of the **unaligned data**.

#### Gene Numbers for Table 1¶

In [3]:

```
# HOMO
cat hg38-tRNAs-noSeC.fas | faswc 
# MAJOR
fasgrep -i "LMAJOR|LTROPICA|LAETHIOPICAL147|LGERBILLILEM452|LTURANICALEM423|LARABICALEM1108" final_CIF_geneset.fasta | faswc
# INFANTUM
fasgrep -i "LINFANTUMJPCM5|LDONOVANI" final_CIF_geneset.fasta | faswc
# MEXICANA
fasgrep -i "LMEXICANA|LAMAZONENSIS" final_CIF_geneset.fasta | faswc
# VIANNIA
fasgrep -i "LPANAMENSIS|LBRAZILIENSIS" final_CIF_geneset.fasta | faswc
# ENRIETTII
fasgrep -i "LENRIETTII|LSPMARLEM" final_CIF_geneset.fasta | faswc
# LEPTOCRITH
fasgrep -i "LSEYMOURIA|LPYRRHOCORI|CFASCICULATACFCL" final_CIF_geneset.fasta | faswc
# AMTRYP
fasgrep -i "TGRAYIANR4|TCRUZI" final_CIF_geneset.fasta | faswc
# AFRTYP
fasgrep -i "TBRUCEI|TCONGOLENSE|TEVANSI|TVIVAXY486" final_CIF_geneset.fasta | faswc
```

```
       431     32403 total
       664     49614 total
       250     18527 total
       148     10992 total
       327     24311 total
       160     11889 total
       300     22211 total
       773     57497 total
       402     29837 total
```

#### Gene Compositions for Table 1¶

In [27]:

```
# HOMO
cat hg38-tRNAs-noSeC.fas | fastr -s 'acgt' 'ACGT' | fascomp -nt | grep \#
# MAJOR
fasgrep -i "LMAJOR|LTROPICA|LAETHIOPICAL147|LGERBILLILEM452|LTURANICALEM423|LARABICALEM1108" final_CIF_geneset.fasta | fastr -s 'acgt' 'ACGT' | fascomp -nt | grep \# 
# INFANTUM
fasgrep -i "LINFANTUMJPCM5|LDONOVANI" final_CIF_geneset.fasta | fastr -s 'acgt' 'ACGT' | fascomp -nt | grep \# 
# MEXICANA
fasgrep -i "LMEXICANA|LAMAZONENSIS" final_CIF_geneset.fasta | fastr -s 'acgt' 'ACGT' | fascomp -nt | grep \# 
# VIANNIA
fasgrep -i "LPANAMENSIS|LBRAZILIENSIS" final_CIF_geneset.fasta | fastr -s 'acgt' 'ACGT' | fascomp -nt | grep \# 
# ENRIETTII
fasgrep -i "LENRIETTII|LSPMARLEM" final_CIF_geneset.fasta | fastr -s 'acgt' 'ACGT' | fascomp -nt | grep \# 
# LEPTOCRITH
fasgrep -i "LSEYMOURIA|LPYRRHOCORI|CFASCICULATACFCL" final_CIF_geneset.fasta | fastr -s 'acgt' 'ACGT' | fascomp -nt | grep \# 
# AMTRYP
fasgrep -i "TGRAYIANR4|TCRUZI" final_CIF_geneset.fasta | fastr -s 'acgt' 'ACGT' | fascomp -nt | grep \# 
# AFRTYP
fasgrep -i "TBRUCEI|TCONGOLENSE|TEVANSI|TVIVAXY486" final_CIF_geneset.fasta | fastr -s 'acgt' 'ACGT' | fascomp -nt | grep \#
```

```
A:0.189  C:0.257  G:0.325  T:0.230   # ALL DATA
A:0.187  C:0.261  G:0.319  T:0.234   # ALL DATA
A:0.188  C:0.261  G:0.319  T:0.233   # ALL DATA
A:0.189  C:0.259  G:0.318  T:0.234   # ALL DATA
A:0.188  C:0.261  G:0.318  T:0.234   # ALL DATA
A:0.188  C:0.259  G:0.319  T:0.234   # ALL DATA
A:0.189  C:0.260  G:0.318  T:0.233   # ALL DATA
A:0.187  C:0.263  G:0.320  T:0.230   # ALL DATA
A:0.186  C:0.261  G:0.322  T:0.231   # ALL DATA
```

## Partition into Alignments by Defined Clades¶

In the following we exclude 464 genes from genomes BAYALAIB08-376 EMONTEROGEIILV88 LTARENTOLAEPARROTTARII PCONFUSUMCUL13 TTHEILERIEDINBURGH

In [28]:

```
fasgrep -i "LMAJOR|LTROPICA|LAETHIOPICAL147|LGERBILLILEM452|LTURANICALEM423|LARABICALEM1108" covea.74sites.fas > covea.74sites.MAJOR.fas
fasgrep -i "LINFANTUMJPCM5|LDONOVANI" covea.74sites.fas                 > covea.74sites.INFANTUM.fas 
fasgrep -i "LMEXICANA|LAMAZONENSIS" covea.74sites.fas                   > covea.74sites.MEXICANA.fas 
fasgrep -i "LPANAMENSIS|LBRAZILIENSIS" covea.74sites.fas                > covea.74sites.VIANNIA.fas
fasgrep -i "LENRIETTII|LSPMARLEM" covea.74sites.fas                     > covea.74sites.ENRIETTII.fas
fasgrep -i "LSEYMOURIA|LPYRRHOCORI|CFASCICULATACFCL" covea.74sites.fas  > covea.74sites.LEPTOCRITH.fas 
fasgrep -i "TGRAYIANR4|TCRUZI" covea.74sites.fas                        > covea.74sites.AMTRYP.fas 
fasgrep -i "TBRUCEI|TCONGOLENSE|TEVANSI|TVIVAXY486" covea.74sites.fas   > covea.74sites.AFTRYP.fas 
fasgrep -i "HOMO" covea.74sites.fas                                     > covea.74sites.HOMO.fas 
faswc covea.74sites.*.fas # 3455 sequences
```

```
       402     29748 covea.74sites.AFTRYP.fas
       773     57202 covea.74sites.AMTRYP.fas
       160     11840 covea.74sites.ENRIETTII.fas
       431     31894 covea.74sites.HOMO.fas
       250     18500 covea.74sites.INFANTUM.fas
       300     22200 covea.74sites.LEPTOCRITH.fas
       664     49136 covea.74sites.MAJOR.fas
       148     10952 covea.74sites.MEXICANA.fas
       327     24198 covea.74sites.VIANNIA.fas
      3455    255670 total
```

### Numbers of Genes Included and Excluded and Genomes Excluded¶

In [38]:

```
fasgrep -i "LMAJOR|LTROPICA|LAETHIOPICAL147|LGERBILLILEM452|LTURANICALEM423|LARABICALEM1108|LINFANTUMJPCM5|LDONOVANI|LMEXICANA|LAMAZONENSIS|LPANAMENSIS|LBRAZILIENSIS|LENRIETTII|LSPMARLEM|LSEYMOURIA|LPYRRHOCORI|CFASCICULATACFCL|TGRAYIANR4|TCRUZI|TBRUCEI|TCONGOLENSE|TEVANSI|TVIVAXY486" final_CIF_geneset.fasta | faswc
fasgrep -iv "LMAJOR|LTROPICA|LAETHIOPICAL147|LGERBILLILEM452|LTURANICALEM423|LARABICALEM1108|LINFANTUMJPCM5|LDONOVANI|LMEXICANA|LAMAZONENSIS|LPANAMENSIS|LBRAZILIENSIS|LENRIETTII|LSPMARLEM|LSEYMOURIA|LPYRRHOCORI|CFASCICULATACFCL|TGRAYIANR4|TCRUZI|TBRUCEI|TCONGOLENSE|TEVANSI|TVIVAXY486" final_CIF_geneset.fasta | faswc
fasgrep -iv "LMAJOR|LTROPICA|LAETHIOPICAL147|LGERBILLILEM452|LTURANICALEM423|LARABICALEM1108|LINFANTUMJPCM5|LDONOVANI|LMEXICANA|LAMAZONENSIS|LPANAMENSIS|LBRAZILIENSIS|LENRIETTII|LSPMARLEM|LSEYMOURIA|LPYRRHOCORI|CFASCICULATACFCL|TGRAYIANR4|TCRUZI|TBRUCEI|TCONGOLENSE|TEVANSI|TVIVAXY486" final_CIF_geneset.fasta | grep \> | perl -pe 's/_.*//' | sort | uniq -c
```

```
      3024    224878 total
       464     34658 total
  68 >BayalaiB08-376
 102 >EmonterogeiiLV88
  77 >LtarentolaeParrotTarII
  61 >PconfusumCUL13
 156 >TtheileriEdinburgh
```

# Prepare Subdirectories for Organizing Inputs and Results¶

In [8]:

```
for x in {"KLD_Logo_Results","Function_Logo_Results","ID_Logo_Results","tsfm_input_alignments"}; do mkdir $x; cd $x; for y in {"MAJOR","INFANTUM","MEXICANA","VIANNIA","ENRIETTII","LEPTOCRITH","AMTRYP","AFTRYP","HOMO"}; do mkdir $y; done; cd ..; done
mkdir Bubble_Plots
```

```
mkdir: tsfm_input_alignments: File exists
mkdir: MAJOR: File exists
mkdir: INFANTUM: File exists
mkdir: MEXICANA: File exists
mkdir: VIANNIA: File exists
mkdir: ENRIETTII: File exists
mkdir: LEPTOCRITH: File exists
mkdir: AMTRYP: File exists
mkdir: AFTRYP: File exists
mkdir: HOMO: File exists
```

# Prepare tSFM Input¶

In [ ]:

```
perl -e 'foreach my $clade (qw/MAJOR INFANTUM MEXICANA VIANNIA ENRIETTII LEPTOCRITH AMTRYP AFTRYP/){
           foreach my $f (split //,"ACDEFGHIKLMNPQRSTVWXY"){
             system "fasgrep -i \"_(ARA)?$f\$\" covea.74sites.$clade.fas | fasconvert -o clustalw > tsfm_input_alignments/$clade/${clade}_$f.aln"}}'
perl -e '%M = (A=>"ALA",C=>"CYS",D=>"ASP",E=>"GLU",F=>"PHE",G=>"GLY",H=>"HIS",I=>"ILE",K=>"LYS",L=>"LEU",M=>"MET",N=>"ASN",P=>"PRO",Q=>"GLN",R=>"ARG",S=>"SER",T=>"THR",V=>"VAL",W=>"TRP",X=>"IMET",Y=>"TYR");
           foreach my $f (split //,"ACDEFGHIKLMNPQRSTVWXY"){
             system "fasgrep -i -- \"-$M{$f}-\" covea.74sites.HOMO.fas | fasconvert -o clustalw > tsfm_input_alignments/HOMO/HOMO_$f.aln"}'
```

In [5]:

```
ls -1 tsfm_input_alignments/*/*.aln | wc # 189 files = 21 functions by 9 clades
cat tsfm_input_alignments/*/*.aln | fasconvert -i clustalw | faswc # 3455 sequences
```

```
     189     189    8316
      3455    255670 total
```

# Generate Logos with tSFM¶

## Function Logos¶

### Computation of Function Logos¶

In [9]:

```
perl -e 'foreach my $clade (qw/HOMO MAJOR INFANTUM MEXICANA VIANNIA ENRIETTII LEPTOCRITH AMTRYP AFTRYP/){system "tsfm -x 1 -c tRNA_L_skel_Leish.sites74.struct.cove --logo tsfm_input_alignments/$clade/$clade; mv -- *.eps $clade*.txt Function_Logo_Results/$clade/";}'
```

```
Parsing base-pair coordinates
21 alignments parsed
Calculating Sample Size Correction for HOMO
 1 0.00000
Calculating information statistics for HOMO using NSB estimator
Writing text output for HOMO
Writing function logo postscript files for HOMO
Parsing base-pair coordinates
21 alignments parsed
Calculating Sample Size Correction for MAJOR
 1 0.00000
Calculating information statistics for MAJOR using NSB estimator
Writing text output for MAJOR
Writing function logo postscript files for MAJOR
Parsing base-pair coordinates
21 alignments parsed
Calculating Sample Size Correction for INFANTUM
 1 0.00000
Calculating information statistics for INFANTUM using NSB estimator
Writing text output for INFANTUM
Writing function logo postscript files for INFANTUM
Parsing base-pair coordinates
21 alignments parsed
Calculating Sample Size Correction for MEXICANA
 1 0.00000
Calculating information statistics for MEXICANA using NSB estimator
Writing text output for MEXICANA
Writing function logo postscript files for MEXICANA
Parsing base-pair coordinates
21 alignments parsed
Calculating Sample Size Correction for VIANNIA
 1 0.00000
Calculating information statistics for VIANNIA using NSB estimator
Writing text output for VIANNIA
Writing function logo postscript files for VIANNIA
Parsing base-pair coordinates
21 alignments parsed
Calculating Sample Size Correction for ENRIETTII
 1 0.00000
Calculating information statistics for ENRIETTII using NSB estimator
Writing text output for ENRIETTII
Writing function logo postscript files for ENRIETTII
Parsing base-pair coordinates
21 alignments parsed
Calculating Sample Size Correction for LEPTOCRITH
 1 0.00000
Calculating information statistics for LEPTOCRITH using NSB estimator
Writing text output for LEPTOCRITH
Writing function logo postscript files for LEPTOCRITH
Parsing base-pair coordinates
21 alignments parsed
Calculating Sample Size Correction for AMTRYP
 1 0.00000
Calculating information statistics for AMTRYP using NSB estimator
Writing text output for AMTRYP
Writing function logo postscript files for AMTRYP
Parsing base-pair coordinates
21 alignments parsed
Calculating Sample Size Correction for AFTRYP
 1 0.00000
Calculating information statistics for AFTRYP using NSB estimator
Writing text output for AFTRYP
Writing function logo postscript files for AFTRYP
```

### Fix Site Coordinate Labels, Bounding Boxes, Rendering in EPS logos for Single-Site Function Logos¶

In [10]:

```
perl fix_coordinate_labels_logofiles_TSFM.pl tRNA_L_skel_Leish.sites74.txt Function_Logo_Results/*/*.eps
perl reformat_logo_files.pl Function_Logo_Results/*/*.eps
```

```
1111111111111111111111111111111111111111111111111111111111111111111111111111111111111111111111111111111111111111111111111111111111111111111111111111111111111111111111111111111111111
```

### Label Single-Site Function Logos Taxonomically¶

In [ ]:

```
perl -e '%names = (HOMO => "Human",MAJOR => "Major Clade", INFANTUM => "Infantum Clade", MEXICANA => "Mexicana Clade", VIANNIA => "Viannia Clade", ENRIETTII => "Enriettii Clade", LEPTOCRITH => "Leptomonas/Crithidia Clade", "AMTRYP" => "American Trypanosoma", AFTRYP => "African Trypanosoma"); @n=qw/A C G U/; foreach my $clade (keys %names) {foreach my $n (@n) {system "convert -font Arial -pointsize 18 -annotate +80+60 \"$names{$clade}\"  -define profile:skip=ICC -colorspace sRGB -density 200 Function_Logo_Results/$clade/${n}_$clade.eps Function_Logo_Results/$clade/${n}_$clade.png";}}'
```

### Assemble Final Single-Site Function Logo Figures 5 for Supplementary Figures 22 through 29¶

In [ ]:

```
perl -e '%names = (A => "Adenine",C => "Cytosine", G => "Guanine", U => "Uracil"); foreach my $n (split //,"AUGC"){system "montage Function_Logo_Results/HOMO/${n}_HOMO.png Function_Logo_Results/MAJOR/${n}_MAJOR.png Function_Logo_Results/INFANTUM/${n}_INFANTUM.png Function_Logo_Results/MEXICANA/${n}_MEXICANA.png Function_Logo_Results/VIANNIA/${n}_VIANNIA.png        -font Arial -pointsize 32 -title \"Function Logos for $names{$n}\" -geometry +1+1 -tile 1x5 ${n}_function_1.png";}'
perl -e '%names = (A => "Adenine",C => "Cytosine", G => "Guanine", U => "Uracil"); foreach my $n (split //,"AUGC"){system "montage Function_Logo_Results/HOMO/${n}_HOMO.png Function_Logo_Results/ENRIETTII/${n}_ENRIETTII.png Function_Logo_Results/LEPTOCRITH/${n}_LEPTOCRITH.png Function_Logo_Results/AMTRYP/${n}_AMTRYP.png Function_Logo_Results/AFTRYP/${n}_AFTRYP.png  -font Arial -pointsize 32 -title \"Function Logos for $names{$n}\" -geometry +1+1 -tile 1x5 ${n}_function_2.png";}'
```

### Label Base-Pair Function Logos Structurally¶

In [ ]:

```
perl -e '@clades = qw/HOMO MAJOR INFANTUM MEXICANA VIANNIA ENRIETTII LEPTOCRITH AMTRYP AFTRYP/; @pairs = qw/UA UG UC UU CA CG CC CU GA GG GC GU AA AG AC AU/; %labels = (UA => "U:A Pair",UG => "U•G Pair", UC => "U-C Mispair", UU => "U-U Mispair", CA => "C-A Mispair", CG => "C:G Pair", CC => "C-C Mispair", CU => "C-U Mispair", GA => "G-A Mispair", GG => "G-G Mispair", GC => "G:C Pair", GU => "G•U Pair", AA => "A-A Mispair", AG => "A-G Mispair", AC => "A–C Mispair", AU => "A:U Pair"); foreach my $clade (@clades) { foreach my $bp (@pairs) {my $filenm = "Function_Logo_Results/$clade/${bp}_$clade.eps"; if (-e -f -r $filenm) { system "convert -font Arial -pointsize 18 -annotate +80+60 \"$labels{$bp}\"  -define profile:skip=ICC -colorspace sRGB -density 200 $filenm Function_Logo_Results/$clade/${bp}_$clade.png"; }}}'
```

### Assemble Final Base-Pair Function Logo Figures for Figures 6-8 and Supp Figs 30-37¶

In [ ]:

```
perl -e '%names = (HOMO => "Humans (n = 1)",MAJOR => "Major Clade (n = 8)", INFANTUM => "Infantum Clade (n = 3)", MEXICANA => "Mexicana Clade (n = 2)", VIANNIA => "Viannia Clade (n = 4)", ENRIETTII => "Enriettii Clade (n = 2)", LEPTOCRITH => "Leptomonas/Crithidia Clade  (n = 3)", AMTRYP => "American Trypanosoma (n = 11)", AFTRYP => "African Trypanosoma (n = 6)"); foreach my $clade (keys %names) { @files=();foreach my $n (qw/UA UG UC UU CA CG CC CU GA GG GC GU AA AG AC AU/){  $filenm = "Function_Logo_Results/$clade/${n}_$clade.png"; if (-e -f -r $filenm) {push @files,$filenm;}else{push @files,"null.png"}} warn (join "","$clade: number of files != 16: ",@files,"\n") unless (@files == 16); system "montage -geometry +1+1 -tile 4x4 @files -font Arial -pointsize 32 -title \"Base-Pair Function Logos for $names{$clade}\" ${clade}_base-pair_function.png";}'
```

## KLD and ID Logos¶

### Computation of KLD and ID Logos¶

In [17]:

```
inputpath="tsfm_input_alignments"
clades_list=$(ls -l "${inputpath}" | grep "^d" | awk -F" " '{print $9}')
for clade in $clades_list; do
if [ $clade != "HOMO" ]
then
  tsfm -c tRNA_L_skel_Leish.sites74.struct.txt -x 1 --idlogo --kldlogo --bt "$inputpath/$clade/$clade" "$inputpath/HOMO/HOMO"
  mv -- *_results.txt
  mv -- *ID*.eps "ID_Logo_Results/$clade/"
  mv -- *KLD*.eps "KLD_Logo_Results/$clade/"
  mv *_Table.txt "Bubble_Plots"
  rm *_results.txt
fi
done
```

```
Parsing base-pair coordinates
Parsing base-pair coordinates
21 alignments parsed
21 alignments parsed
Calculating Sample Size Correction for AFTRYP
 1 0.00000
Calculating Sample Size Correction for HOMO
 1 0.00000
Calculating information statistics for AFTRYP using NSB estimator
Calculating information statistics for HOMO using NSB estimator
Writing text output for AFTRYP
Writing text output for HOMO
Parsing base-pair coordinates
Parsing base-pair coordinates
21 alignments parsed
21 alignments parsed
Calculating Sample Size Correction for AMTRYP
 1 0.00000
Calculating Sample Size Correction for HOMO
 1 0.00000
Calculating information statistics for AMTRYP using NSB estimator
Calculating information statistics for HOMO using NSB estimator
Writing text output for AMTRYP
Writing text output for HOMO
Parsing base-pair coordinates
Parsing base-pair coordinates
21 alignments parsed
21 alignments parsed
Calculating Sample Size Correction for ENRIETTII
 1 0.00000
Calculating Sample Size Correction for HOMO
 1 0.00000
Calculating information statistics for ENRIETTII using NSB estimator
Calculating information statistics for HOMO using NSB estimator
Writing text output for ENRIETTII
Writing text output for HOMO
Parsing base-pair coordinates
Parsing base-pair coordinates
21 alignments parsed
21 alignments parsed
Calculating Sample Size Correction for INFANTUM
 1 0.00000
Calculating Sample Size Correction for HOMO
 1 0.00000
Calculating information statistics for INFANTUM using NSB estimator
Calculating information statistics for HOMO using NSB estimator
Writing text output for INFANTUM
Writing text output for HOMO
Parsing base-pair coordinates
Parsing base-pair coordinates
21 alignments parsed
21 alignments parsed
Calculating Sample Size Correction for LEPTOCRITH
 1 0.00000
Calculating Sample Size Correction for HOMO
 1 0.00000
Calculating information statistics for LEPTOCRITH using NSB estimator
Calculating information statistics for HOMO using NSB estimator
Writing text output for LEPTOCRITH
Writing text output for HOMO
Parsing base-pair coordinates
Parsing base-pair coordinates
21 alignments parsed
21 alignments parsed
Calculating Sample Size Correction for MAJOR
 1 0.00000
Calculating Sample Size Correction for HOMO
 1 0.00000
Calculating information statistics for MAJOR using NSB estimator
Calculating information statistics for HOMO using NSB estimator
Writing text output for MAJOR
Writing text output for HOMO
Parsing base-pair coordinates
Parsing base-pair coordinates
21 alignments parsed
21 alignments parsed
Calculating Sample Size Correction for MEXICANA
 1 0.00000
Calculating Sample Size Correction for HOMO
 1 0.00000
Calculating information statistics for MEXICANA using NSB estimator
Calculating information statistics for HOMO using NSB estimator
Writing text output for MEXICANA
Writing text output for HOMO
Parsing base-pair coordinates
Parsing base-pair coordinates
21 alignments parsed
21 alignments parsed
Calculating Sample Size Correction for VIANNIA
 1 0.00000
Calculating Sample Size Correction for HOMO
 1 0.00000
Calculating information statistics for VIANNIA using NSB estimator
Calculating information statistics for HOMO using NSB estimator
Writing text output for VIANNIA
Writing text output for HOMO
```

# Generate Bubble-Plots¶

## Map the text output of tsfm (Tables) in folder Bubble\_Plots to Sprinzl Coordinates¶

This also updates the text files by adding three columns to the tables.

In [19]:

```
R --vanilla < Map2Sprinzl.R
```

```
R version 3.5.1 (2018-07-02) -- "Feather Spray"
Copyright (C) 2018 The R Foundation for Statistical Computing
Platform: x86_64-apple-darwin15.6.0 (64-bit)

R is free software and comes with ABSOLUTELY NO WARRANTY.
You are welcome to redistribute it under certain conditions.
Type 'license()' or 'licence()' for distribution details.

  Natural language support but running in an English locale

R is a collaborative project with many contributors.
Type 'contributors()' for more information and
'citation()' on how to cite R or R packages in publications.

Type 'demo()' for some demos, 'help()' for on-line help, or
'help.start()' for an HTML browser interface to help.
Type 'q()' to quit R.

> # This script will map the text output of tsfm in folder Bubble_Plots to sprinzl coordinates and update the text files.
> # install package gdata using the command install.packages("gdata")
> 
> library(gdata)
gdata: read.xls support for 'XLS' (Excel 97-2004) files ENABLED.

gdata: read.xls support for 'XLSX' (Excel 2007+) files ENABLED.

Attaching package: ‘gdata’

The following object is masked from ‘package:stats’:

    nobs

The following object is masked from ‘package:utils’:

    object.size

The following object is masked from ‘package:base’:

    startsWith

> Outputpath <- "./Bubble_Plots/"
> clades_dir <- "./tsfm_input_alignments/"
> skelfile_path <- "tRNA_L_skel_Leish.sites74.txt"
> match_bubble_coords <- function(df, tRNA_L_skel_df) {
+   tRNA_L_skel_df$sprinzl <- as.character(tRNA_L_skel_df$sprinzl)
+   tRNA_L_skel_df$sprinzl2 <- as.character(tRNA_L_skel_df$sprinzl2)
+   tRNA_L_skel_df <- tRNA_L_skel_df[!is.na(tRNA_L_skel_df$sprinzl),]
+   for (i in 1:nrow(df)) {
+     samecoord = tRNA_L_skel_df$sprinzl == df$coord[i]
+     df$x[i] = tRNA_L_skel_df[samecoord,]$x
+     df$y[i] = tRNA_L_skel_df[samecoord,]$y
+     df$sprinzl[i] = tRNA_L_skel_df[samecoord,]$sprinzl2
+   }
+   df
+ }
> tRNA_L_skel_df <- read.table(skelfile_path, header = FALSE,sep = ",")
> names(tRNA_L_skel_df) <- c("sprinzl", "x", "y", "sprinzl2")
> clusterdir <- list.dirs(path = clades_dir, recursive = FALSE)
> for (i in 1:length(clusterdir)) {
+   splitedpath <- unlist(strsplit(clusterdir[i], split = "/"))
+   clade_name <- splitedpath[length(splitedpath)]
+   table_name <- paste(clade_name, "_Table.txt", sep = "")
+   df <-
+     read.table(paste(Outputpath, table_name, sep = ""), header = TRUE)
+   df <- match_bubble_coords(df, tRNA_L_skel_df)
+   # write tables in fix length format
+   n <-
+     data.frame(
+       "aa"      ,
+       "coord" ,
+       "state"  ,
+       "fbits" ,
+       "fht"   ,
+       "gainbits",
+       "gainfht" ,
+       "lossbits" ,
+       "lossfht" ,
+       "convbits" ,
+       "convfht" ,
+       "x"  ,
+       "y"     ,
+       "sprinzl"
+     )
+   names(n) <- names(df)
+   for (i in 1:ncol(df)) {
+     df[, i] <- as.character(df[, i])
+   }
+   write.fwf(
+     rbind(n, df),
+     colnames = FALSE,
+     width = rep(10, 14),
+     file = paste(Outputpath, table_name, sep = "")
+   )
+ }
> 
>
```

## Generate Bubble Plots¶

In [20]:

```
R --vanilla < bubble.R
```

```
R version 3.5.1 (2018-07-02) -- "Feather Spray"
Copyright (C) 2018 The R Foundation for Statistical Computing
Platform: x86_64-apple-darwin15.6.0 (64-bit)

R is free software and comes with ABSOLUTELY NO WARRANTY.
You are welcome to redistribute it under certain conditions.
Type 'license()' or 'licence()' for distribution details.

  Natural language support but running in an English locale

R is a collaborative project with many contributors.
Type 'contributors()' for more information and
'citation()' on how to cite R or R packages in publications.

Type 'demo()' for some demos, 'help()' for on-line help, or
'help.start()' for an HTML browser interface to help.
Type 'q()' to quit R.

> # make sure your working directory is set to the folder "test".
> # Example: setwd("<PATH TO SOM_KellyEtl_code_and_data/test>")
> 
> MAJOR.74       <- read.table("Bubble_Plots/MAJOR_Table.txt",header=T);
> INFANTUM.74    <- read.table("Bubble_Plots/INFANTUM_Table.txt",header=T);
> MEXICANA.74    <- read.table("Bubble_Plots/MEXICANA_Table.txt",header=T);
> VIANNIA.74     <- read.table("Bubble_Plots/VIANNIA_Table.txt",header=T);
> ENRIETTII.74   <- read.table("Bubble_Plots/ENRIETTII_Table.txt",header=T);
> LEPTOCRITH.74  <- read.table("Bubble_Plots/LEPTOCRITH_Table.txt",header=T);
> AFTRYP.74      <- read.table("Bubble_Plots/AFTRYP_Table.txt",header=T);
> AMTRYP.74      <- read.table("Bubble_Plots/AMTRYP_Table.txt",header=T);
> 
> classes  <- c('L','I','V','R','C','M','E','Q','Y','W','S','T','P','H','G','D','N','K','F','A', 'X');
> ldf.74   <- list(MAJOR.74,INFANTUM.74,MEXICANA.74,VIANNIA.74,AFTRYP.74,AMTRYP.74,LEPTOCRITH.74,ENRIETTII.74);
> df.names <- c("Major (n = 8)","Infantum (n = 3)","Mexicana (n = 2)","Viannia (n = 4)","Af. Tryp. (n = 5)","Am. Tryp. (n = 11)","Lepto/Crith (n = 3)","Enriettii (n = 2)");
> 
> gains <- vector()
> convs <- vector()
> for (class in classes) {
+   i <- 1
+   for (df in ldf.74) {
+     gainvals <- (df$gainbits * df$gainfht);
+     convvals <- (df$convbits * df$convfht);
+     gainvals <- log(gainvals[gainvals>0])
+     convvals <- log(convvals[convvals>0])
+     gains <- append(gains, values=gainvals);
+     convs <- append(convs, values=convvals);
+   }
+ }
> bitdatagains <- data.frame(bits = gains,type=rep("Gain",length(gains)))
> bitdataconvs <- data.frame(bits = convs,type=rep("Conversion",length(convs)))
> bitdata <- rbind(bitdatagains,bitdataconvs)
> library(ggplot2)
Warning message:
package ‘ggplot2’ was built under R version 3.5.2 
> ggplot(bitdata,
+        aes(
+          x = bits,
+          fill = type
+        )) + theme_bw() + geom_density(alpha=0.3) + xlab("log Gain or Conversion Bits") + ggtitle("log-letter-height distributions for Gains and Conversions") +  theme(legend.title=element_blank())
> 
> library("heR.Misc"); ## THIS IS REQUIRED FOR THE BUBBLEPLOT FUNCTION AND MUST BE DOWNLOADED FROM
Loading required package: lattice

Attaching package: ‘heR.Misc’

The following object is masked from ‘package:lattice’:

    panel.superpose.2

The following object is masked from ‘package:ggplot2’:

    annotate

> ## http://exposurescience.org/her.html
> 
> 
> # THESE ARE THE COORDINATES FOR THE TRNA STRUCTURE BACKBONE IN THE FIGURES
> 
> line.x <- c(6.875,6.500,6.125,5.750,5.375,5.000,4.625,4.625,5.000,5.000,2.375
+             ,2.750,2.375,2.750,2.500,2.875,3.250,2.875,2.500,2.125,1.750,1.375,1.000
+             ,0.625,0.250,0.625,1.000,1.500,1.125,1.500,1.125,1.500,1.125,1.500,1.125
+             ,1.500,1.125,0.625,1.000,1.375,1.750,2.125,2.500,2.875,2.375,2.750,2.375
+             ,2.750,2.375,4.250,4.250,3.875,4.250,3.875,4.250,4.250,3.875,3.500,3.125
+             ,2.750,2.250,1.875,1.500,1.125,1.500,1.875,2.250,2.750,3.125,3.500,3.875
+             ,4.250,4.625,5.000,5.375,5.750,6.125,6.500,6.875,7.250,7.625,8.000,8.375);
> 
> line.y <- c(8.875,8.500,8.875,8.500,8.875,8.500,8.875,7.375,7.000
+             ,3.500,3.500,3.875,4.250,4.625,5.125,5.500,5.875,6.250,6.625
+             ,7.000,7.375,7.000,6.625,6.250,5.875,5.500,5.125,4.625,4.250
+             ,3.875,3.500,3.125,2.750,2.375,2.000,1.625,1.250,0.750,0.375
+             ,0.000,-0.375,0.000,0.375,0.750,1.250,1.625,2.000,2.375,2.750
+             ,2.750,3.875,4.250,4.625,5.000,5.375,8.500,8.875,8.500,8.875
+             ,8.500,8.000,8.375,8.750,9.125,9.500,9.875,10.250,9.750,10.125
+             ,9.750,10.125,9.750,10.125,9.750,10.125,9.750,10.125,9.750,10.125
+             ,9.750,10.125,9.750,10.125);
> 
> 
> 
> ## THESE ARE FOR THE SPRINZL COORD LABELS
> coord.labels <- c("1","5","10","14","18","21","25","30","35","40","45","50","55","60","65","70");
> coord.labels.x <- c(6.875,5.375,2.375,2.61,1.750,1.00,1.125,1.500,1.750,2.750,3.65,3.870,1.875,2.250,4.250,6.125);
> coord.labels.y <- c(8.875,8.875,3.500,5.125,7.375,5.125,3.500,1.625,-0.375,1.625,4.680,8.875,8.375,10.250,9.750,10.125);
> 
> xbump <- 0.5;
> ybump <- 0.5;
> 
> up <- c(13,14,15,16,17);
> up.coord.labels <- coord.labels[up];
> up.coord.labels.x <- coord.labels.x[up];
> up.coord.labels.y <- coord.labels.y[up] + ybump;
> 
> dn <- c(1,2,5,9,11,12);
> dn.coord.labels <- coord.labels[dn];
> dn.coord.labels.x <- coord.labels.x[dn];
> dn.coord.labels.y <- coord.labels.y[dn] - ybump;
> 
> lt <- c(3,4,7);
> lt.coord.labels <- coord.labels[lt];
> lt.coord.labels.x <- coord.labels.x[lt] - xbump;
> lt.coord.labels.y <- coord.labels.y[lt];
> 
> rt <- c(6,8,10);
> rt.coord.labels <- coord.labels[rt];
> rt.coord.labels.x <- coord.labels.x[rt] + xbump;
> rt.coord.labels.y <- coord.labels.y[rt];
> 
> 
> alpha=0.5;
> fact=0.5;
> area=TRUE;
> legend=FALSE;  
> 
> map2rgb <- function (c) { rgb(t(col2rgb(c))/255,alpha=alpha);}
> colormap <- function (g,c) { 
+   y <- rep(0,length(g));
+   
+   y[g <  0.48             & c < 0.48]               <- "#FFFFFFC3" 
+   y[g >= 0.48 & g < 1.8   & c < 0.48]               <- "#B87082C3" 
+   y[g >= 1.8             & c < 0.48]               <- "#A2021DC3" 
+   y[g <  0.48             & c >= 0.48 & c < 1.8]    <- "#8190AEC3" 
+   y[g >= 0.48 & g < 1.8   & c >= 0.48 & c < 1.8]    <- "#765C8CC3" 
+   y[g >= 1.8             & c >= 0.48 & c < 1.8]    <- "#B700B7C3" 
+   y[g <  0.48             & c >= 1.8]              <- "#083EAEC3" 
+   y[g >= 0.48 & g < 1.8   & c >= 1.8]              <- "#190081C3" 
+   y[g >= 1.8             & c >= 1.8]              <- "#6C008CC3" 
+   y;
+   
+ }
> 
> 
> widthmap <- function (g,c) {
+   y <- rep(1,length(g));
+   y[g < 0.48 & c < 0.44] <- 1;
+   y[g >= 0.48 | c >= 0.44] <- 2;
+   y[g >= 0.95 | c >= 0.70] <- 3;	
+   y;
+ }
> 
> 
> for (class in classes) {
+   i <- 1
+   filenm <- paste("Bubble_Plots/bubble_",class,".pdf",sep="");
+   pdf(file=filenm,version="1.4",height=3.5);
+   par(mfrow=c(2,4), las=1);
+   for (df in ldf.74) {
+     gains <- (df$gainbits * df$gainfht);
+     convs <- (df$convbits * df$convfht);
+     colors <- colormap(gains,convs);
+     widths <- widthmap(gains,convs);
+     op <- par(mar = rep(0.75, 4))
+     bubbleplot(
+       df$x[df$aa == class],
+       df$y[df$aa == class],
+       (df$fbits[df$aa == class] * df$fht[df$aa == class]),
+       fact=fact, #0.265165 = sqrt(2*0.375^2)/2
+       area=area, 
+       fg = rgb(t(col2rgb("black")/255)),
+       bg = colors[df$aa == class],
+       box=FALSE,
+       axes=FALSE,
+       lwd=0.5,
+       xlim = c(0,8),
+       ylim = c(-0.8,10.8),
+       main = paste(df.names[i],class)
+     );
+     par(op);
+     lines(line.x,line.y);
+     text(labels=up.coord.labels,x=up.coord.labels.x,y=up.coord.labels.y,cex=0.75);
+     text(labels=dn.coord.labels,x=dn.coord.labels.x,y=dn.coord.labels.y,cex=0.75);
+     text(labels=lt.coord.labels,x=lt.coord.labels.x,y=lt.coord.labels.y,cex=0.75);
+     text(labels=rt.coord.labels,x=rt.coord.labels.x,y=rt.coord.labels.y,cex=0.75);
+     prime.x <- c(line.x[1],line.x[83]);
+     prime.y <- c(line.y[1],line.y[83]);
+     text(labels=c("5'","3'"),x=prime.x,y=prime.y,adj=c(-0.9,0),cex=0.75);	
+     if (df.names[i] == "Enriettii (n = 2)") {
+       legend.x <- rep(df$x[df$aa == "X" &  df$state == "A" & 
+                              (df$sprinzl == "69" |  df$sprinzl == "71" |
+                                 df$sprinzl == "73" )] + c(0.2,0.3,0.4),3);  
+       legend.y <- c(rep(df$y[df$aa == "X" &  df$state == "A" & df$sprinzl == "35"]+0.40,3),
+                     rep(df$y[df$aa == "X" &  df$state == "A" & df$sprinzl == "31"],3), 
+                     rep(df$y[df$aa == "X" &  df$state == "A" & df$sprinzl == "27"]-0.30,3));
+       legend.z <- rep(2.2,9);
+       legend.c <- colormap(c(0,0,0,0.5,0.5,0.5,3,3,3),c(0,0.5,3,0,0.5,3,0,0.5,3));
+       bubbleplot(legend.x, legend.y, legend.z,fact=fact,area=area,bg=legend.c,
+                  add=TRUE,
+                  box=FALSE,axes=FALSE,lwd=1);
+     } 
+     i <- i + 1
+   }
+   dev.off()
+ }
Radius scale factor is 0.5 
Radius scale factor is 0.5 
Radius scale factor is 0.5 
Radius scale factor is 0.5 
Radius scale factor is 0.5 
Radius scale factor is 0.5 
Radius scale factor is 0.5 
Radius scale factor is 0.5 
Radius scale factor is 0.5 
Radius scale factor is 0.5 
Radius scale factor is 0.5 
Radius scale factor is 0.5 
Radius scale factor is 0.5 
Radius scale factor is 0.5 
Radius scale factor is 0.5 
Radius scale factor is 0.5 
Radius scale factor is 0.5 
Radius scale factor is 0.5 
Radius scale factor is 0.5 
Radius scale factor is 0.5 
Radius scale factor is 0.5 
Radius scale factor is 0.5 
Radius scale factor is 0.5 
Radius scale factor is 0.5 
Radius scale factor is 0.5 
Radius scale factor is 0.5 
Radius scale factor is 0.5 
Radius scale factor is 0.5 
Radius scale factor is 0.5 
Radius scale factor is 0.5 
Radius scale factor is 0.5 
Radius scale factor is 0.5 
Radius scale factor is 0.5 
Radius scale factor is 0.5 
Radius scale factor is 0.5 
Radius scale factor is 0.5 
Radius scale factor is 0.5 
Radius scale factor is 0.5 
Radius scale factor is 0.5 
Radius scale factor is 0.5 
Radius scale factor is 0.5 
Radius scale factor is 0.5 
Radius scale factor is 0.5 
Radius scale factor is 0.5 
Radius scale factor is 0.5 
Radius scale factor is 0.5 
Radius scale factor is 0.5 
Radius scale factor is 0.5 
Radius scale factor is 0.5 
Radius scale factor is 0.5 
Radius scale factor is 0.5 
Radius scale factor is 0.5 
Radius scale factor is 0.5 
Radius scale factor is 0.5 
Radius scale factor is 0.5 
Radius scale factor is 0.5 
Radius scale factor is 0.5 
Radius scale factor is 0.5 
Radius scale factor is 0.5 
Radius scale factor is 0.5 
Radius scale factor is 0.5 
Radius scale factor is 0.5 
Radius scale factor is 0.5 
Radius scale factor is 0.5 
Radius scale factor is 0.5 
Radius scale factor is 0.5 
Radius scale factor is 0.5 
Radius scale factor is 0.5 
Radius scale factor is 0.5 
Radius scale factor is 0.5 
Radius scale factor is 0.5 
Radius scale factor is 0.5 
Radius scale factor is 0.5 
Radius scale factor is 0.5 
Radius scale factor is 0.5 
Radius scale factor is 0.5 
Radius scale factor is 0.5 
Radius scale factor is 0.5 
Radius scale factor is 0.5 
Radius scale factor is 0.5 
Radius scale factor is 0.5 
Radius scale factor is 0.5 
Radius scale factor is 0.5 
Radius scale factor is 0.5 
Radius scale factor is 0.5 
Radius scale factor is 0.5 
Radius scale factor is 0.5 
Radius scale factor is 0.5 
Radius scale factor is 0.5 
Radius scale factor is 0.5 
Radius scale factor is 0.5 
Radius scale factor is 0.5 
Radius scale factor is 0.5 
Radius scale factor is 0.5 
Radius scale factor is 0.5 
Radius scale factor is 0.5 
Radius scale factor is 0.5 
Radius scale factor is 0.5 
Radius scale factor is 0.5 
Radius scale factor is 0.5 
Radius scale factor is 0.5 
Radius scale factor is 0.5 
Radius scale factor is 0.5 
Radius scale factor is 0.5 
Radius scale factor is 0.5 
Radius scale factor is 0.5 
Radius scale factor is 0.5 
Radius scale factor is 0.5 
Radius scale factor is 0.5 
Radius scale factor is 0.5 
Radius scale factor is 0.5 
Radius scale factor is 0.5 
Radius scale factor is 0.5 
Radius scale factor is 0.5 
Radius scale factor is 0.5 
Radius scale factor is 0.5 
Radius scale factor is 0.5 
Radius scale factor is 0.5 
Radius scale factor is 0.5 
Radius scale factor is 0.5 
Radius scale factor is 0.5 
Radius scale factor is 0.5 
Radius scale factor is 0.5 
Radius scale factor is 0.5 
Radius scale factor is 0.5 
Radius scale factor is 0.5 
Radius scale factor is 0.5 
Radius scale factor is 0.5 
Radius scale factor is 0.5 
Radius scale factor is 0.5 
Radius scale factor is 0.5 
Radius scale factor is 0.5 
Radius scale factor is 0.5 
Radius scale factor is 0.5 
Radius scale factor is 0.5 
Radius scale factor is 0.5 
Radius scale factor is 0.5 
Radius scale factor is 0.5 
Radius scale factor is 0.5 
Radius scale factor is 0.5 
Radius scale factor is 0.5 
Radius scale factor is 0.5 
Radius scale factor is 0.5 
Radius scale factor is 0.5 
Radius scale factor is 0.5 
Radius scale factor is 0.5 
Radius scale factor is 0.5 
Radius scale factor is 0.5 
Radius scale factor is 0.5 
Radius scale factor is 0.5 
Radius scale factor is 0.5 
Radius scale factor is 0.5 
Radius scale factor is 0.5 
Radius scale factor is 0.5 
Radius scale factor is 0.5 
Radius scale factor is 0.5 
Radius scale factor is 0.5 
Radius scale factor is 0.5 
Radius scale factor is 0.5 
Radius scale factor is 0.5 
Radius scale factor is 0.5 
Radius scale factor is 0.5 
Radius scale factor is 0.5 
Radius scale factor is 0.5 
Radius scale factor is 0.5 
Radius scale factor is 0.5 
Radius scale factor is 0.5 
Radius scale factor is 0.5 
Radius scale factor is 0.5 
Radius scale factor is 0.5 
Radius scale factor is 0.5 
Radius scale factor is 0.5 
Radius scale factor is 0.5 
Radius scale factor is 0.5 
Radius scale factor is 0.5 
Radius scale factor is 0.5 
Radius scale factor is 0.5 
Radius scale factor is 0.5 
Radius scale factor is 0.5 
Radius scale factor is 0.5 
Radius scale factor is 0.5 
Radius scale factor is 0.5 
Radius scale factor is 0.5 
Radius scale factor is 0.5 
Radius scale factor is 0.5 
Radius scale factor is 0.5 
Radius scale factor is 0.5 
Radius scale factor is 0.5 
Radius scale factor is 0.5 
>
```

In [ ]:

```

```
