# Supplementary material for: Targeting tRNA-synthetase interactions towards novel therapeutic discovery against eukaryotic pathogens
Source: PLoS Negl Trop Dis. 2020 Feb 27;14(2):e0007983. doi: 10.1371/journal.pntd.0007983 (PMC7046186; doi:10.1371/journal.pntd.0007983)
Supplement: S1 Text — (PDF) [file pntd.0007983.s053.pdf]

Supplementary Text 1 — Supplementary methods for bacterial fermentation, natural product extraction, and active compound identification using HPLC-UV-MS and UPLC-ESI-qTOF-MS.

<u>Bacterial Fermentation and Natural Product Extraction</u>

Frozen stocks of the associated producing organism RL12-182-HVF-D was streaked onto fresh Marine Broth agar plates (Difco, USA) and incubated at room temperature (~ 25 □C) until discrete colonies became visible. Individual colonies were inoculated into 7 mL (small-scale) of modified saline SYP (mSYP) media (10 g starch, 4 g peptone, 2 g yeast extract and 31.2 g instant ocean in 1 L of distilled water) or GNZ media (20 g starch, 10 g glucose, 5 g NZ-amine, 1 g $CaCO_3$, 5 g yeast extract in 1 L of distilled water). Bacterial fermentation was stepped up in stages by inoculating 3 mL of the 7 mL liquid mSYP or GNZ culture into 60 mL (medium-scale) of mSYP or GNZ, respectively. This was followed by inoculating 30 mL of the medium scale culture into 1 L (large-scale) in respective media with 20 g of pre-washed XAD-7 resin (CH_2Cl_2, MeOH and water). Small-scale cultures were incubated for four days, medium-scale cultures for four days and large-scale cultures for seven days, at ~ 25 □C and shaken at 200 RPM.

Large-scale cultures were extracted by first filtering the cellular/resin slurry under vacuum through two layers of Whatman filter paper. The cells, resin and filter paper were extracted twice with 500 mL of 1:1 CH_2Cl_2:MeOH and the suspension stirred for 1 hour. Combined organic extracts were filtered and concentrated to dryness in vacuo. Dried crude extracts for each media (mSYP and GNZ) were fractionated individually by manual solid phase extraction chromatography using Sep-Pak (SP) columns (5 g C18 cartridge, Supelco, USA). Chromatography proceeded using a stepwise MeOH/H_2¬O gradient: 40 mL of 10% MeOH (wash), 20% (fraction A), 40% (fraction B), 60% (fraction C), 80% (fraction D), 100% (fraction E) then 100% EtOAc wash (fraction F). SP fractions A – F were concentrated to dryness in vacuo and prepared for HPLC-UV separation.

<u>HPLC-UV-MS and UPLC-ESI-qTOF-MS Analyses</u>

All high-performance liquid chromatography analyses were performed on an Agilent 1200 series HPLC system equipped with both an Agilent photodiode array (PDA) detector and an Agilent 6130 single quadrupole mass spectrometer to acquire UV and MS data respectively. Samples were injected onto a C18 reverse-phase column (Synergi 10□ Fusion RP Column, Phenomenex, USA) using a H_2O:MeOH (0.02% formic acid) elution profile: 0 – 3 mins, 5% MeOH; 3 – 25 min, linear gradient 5% to 100% MeOH; 25 – 30 min, isocratic at 100% MeOH; 30 – 35 min, isocratic at 5% MeOH, using a flow rate of 2 mL/min and positive ESI mode.

Accurate mass MS/MS data were acquired on an Acquity i-Class UPLC system (Waters Corporation) with SYNAPT G2-Si qTOF mass spectrometer (Waters Corporation) run in HRMS positive ESI mode. The instrument was operated using a 20 □g/mL leucine enkephalin lockspray infusion injected every 10 seconds to control mass accuracy. Samples are injected onto a C18 reverse-phase column (HSS C18, 100 mm x 2.1 mm, 1.7□m, Waters Corporation) using a H_2O (0.1% formic acid):ACN (0.1% formic acid) elution profile: 0 – 0.3 min, 5% ACN; 0.3 – 4.7 min, linear gradient 5% to 90% ACN; 4.7 – 5.5 min, linear gradient 90 to 98% ACN; 5.5 – 5.8 min, isocratic at 98% ACN; 5.81 – 7.5 min, isocratic at 5% ACN, using a flow rate of 0.5 mL/min.
